# A Fast-Binding, Functionally Reversible, COX-2 Radiotracer for CNS PET Imaging

**DOI:** 10.1021/acscentsci.3c01564

**Published:** 2024-04-25

**Authors:** Michael S. Placzek, Daniel K. Wilton, Michel Weïwer, Mariah A. Manter, Sarah E. Reid, Christopher J. Meyer, Arthur J. Campbell, Besnik Bajrami, Antoine Bigot, Sarah Bricault, Agathe Fayet, Arnaud Frouin, Frederick Gergits, Mehak Gupta, Wei Jiang, Michelle Melanson, Chiara D. Romano, Misha M. Riley, Jessica M. Wang, Hsiao-Ying Wey, Florence F. Wagner, Beth Stevens, Jacob M. Hooker

**Affiliations:** † Athinoula A. Martinos Center for Biomedical Imaging, Massachusetts General Hospital and Harvard Medical School, Charlestown, Massachusetts 02129, United States; ‡ Department of Neurology and F.M. Kirby Neurobiology Center, Boston Children’s Hospital, Harvard Medical School, Boston, Massachusetts 02115, United States; § Center for the Development of Therapeutics, Broad Institute of MIT and Harvard, 75 Ames Street, Cambridge, Massachusetts 02142, United States; ⊥ Lurie Center for Autism, 1 Maguire Road, Lexington, Massachusetts 02421, United States; ¶ Massachusetts General Hospital, 55 Fruit St., Boston, Massachusetts 02114, United States; € Stanley Center for Psychiatric Research, Broad Institute of MIT and Harvard, 75 Ames Street, Cambridge, Massachusetts 02142, United; ★ Howard Hughes Medical Institute, Boston Children’s Hospital, Harvard Medical School, Boston, Massachusetts 02115, United States

## Abstract

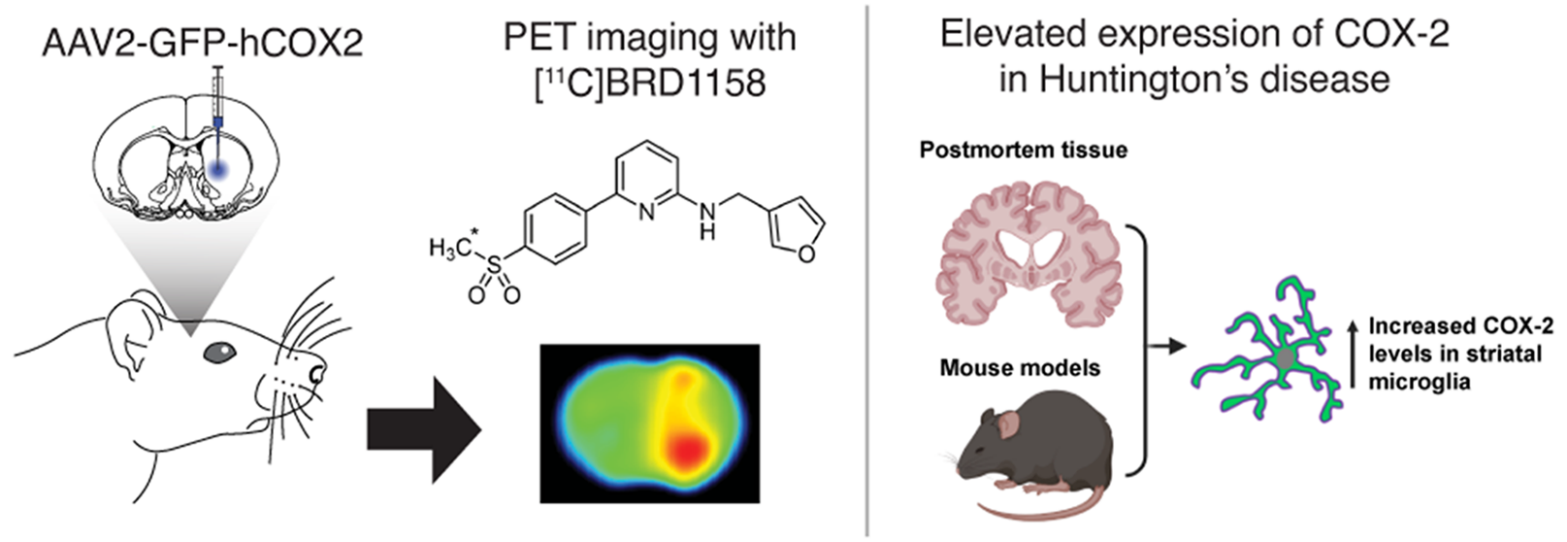

Cyclooxygenase-2 (COX-2) is an enzyme that plays a pivotal role in peripheral inflammation and pain via the prostaglandin pathway. In the central nervous system (CNS), COX-2 is implicated in neurodegenerative and psychiatric disorders as a potential therapeutic target and biomarker. However, clinical studies with COX-2 have yielded inconsistent results, partly due to limited mechanistic understanding of how COX-2 activity relates to CNS pathology. Therefore, developing COX-2 positron emission tomography (PET) radiotracers for human neuroimaging is of interest. This study introduces [^11^C]BRD1158, which is a potent and uniquely fast-binding, selective COX-2 PET radiotracer. [^11^C]BRD1158 was developed by prioritizing potency at COX-2, isoform selectivity over COX-1, fast binding kinetics, and free fraction in the brain. Evaluated through in vivo PET neuroimaging in rodent models with human COX-2 overexpression, [^11^C]BRD1158 demonstrated high brain uptake, fast target-engagement, functional reversibility, and excellent specific binding, which is advantageous for human imaging applications. Lastly, post-mortem samples from Huntington’s disease (HD) patients and preclinical HD mouse models showed that COX-2 levels were elevated specifically in disease-affected brain regions, primarily from increased expression in microglia. These findings indicate that COX-2 holds promise as a novel clinical marker of HD onset and progression, one of many potential applications of [^11^C]BRD1158 human PET.

## Abstract 2

A COX-2 specific radiotracer for positron emission tomography imaging, [^11^C]BRD1158, was developed and evaluated in rodent models. [^11^C]BRD1158 demonstrated rapid binding and functional reversibility and shows promise for human translation. Additionally, evidence for the involvement of COX-2 in Huntington’s disease is presented, highlighting the relevance of [^11^C]BRD1158 to clinical and research applications in neurodegeneration.

## Introduction

Cyclooxygenase-2 (COX-2) is a pivotal enzyme in the prostaglandin biosynthesis pathway and is well-known as a therapeutic target of nonsteroidal anti-inflammatory drugs (NSAIDs).^1,2^ COX-2 is upregulated locally when tissues are injured in the periphery and in the spinal cord.^3^ The upregulation of COX-2 leads to increased synthesis of prostaglandins^4^ that initiate the inflammatory response, including vasodilation, increased microvascular permeability, and sensitization of nerves.^5^ The inhibition of COX-2 prevents this cascade, reducing pain and swelling.^2^

COX-2 activity in the brain is implicated in many neurodegenerative and psychiatric disorders that comprise a significant portion of the main contributors to global disease burden.^6−9^ However, the specific role of COX-2 in the central nervous system (CNS) remains unclear, even amidst strong associations of increased COX-2 expression with the pathology of neurodegenerative diseases, including Alzheimer’s disease (AD)^10,11^ and multiple sclerosis (MS).^12^ Both COX-2 and COX-2 metabolites are elevated in the post-mortem brain tissue of patients diagnosed with these neurodegenerative diseases. In addition, investigations using transgenic mice and COX-2 selective inhibitors have demonstrated that suppression of COX-2 activity in both AD and MS mouse models attenuates neuropathological hallmarks of these diseases such as Aβ plaque deposition, tau phosphorylation, and myelin degeneration, while also improving performance mice in motor and cognitive tasks.^13−18^

Given the association of COX-2 with peripheral inflammation, its role in the brain has often been linked to a neuroinflammatory or neuroimmune function—and, thus, has been linked to microglial phenotype changes. Several studies have found COX-2 to be differentially regulated in macrophages or microglia under conditions of pro-inflammatory brain states and neurodegeneration.^6^ For example, gene expression datasets from lipopolysaccharide (LPS)-injected mice and an AD mouse model showed increased COX-2 expression in microglia, compared to their respective controls (GEO: GSE67858, GSE75246, GSE74615).^19,20^ In addition, COX-2 was identified as part of the transcriptional signature of a neurodegenerative specific microglial phenotype (MGnD) that was characterized through studies of common microglial gene changes in models of amyotrophic lateral sclerosis (ALS), AD, and MS.^21^ A separate study also identified COX-2 as part of a gene module that was upregulated in mice who underwent LPS treatment and in mouse models of AD.^22^ Although the role of COX-2 expression in microglia is not entirely clear, microglial COX-2/prostaglandin E2 (PGE_2_) activity appears to hamper beneficial microglial functions and drive aspects of pathology in rodent models of AD.^23,24^ In addition, transcriptomic analysis of human cortical tissue shows changes in COX-2 expression in microglia across the human lifespan (GEO: GSE99074).^19,20^ Taken together, these findings make microglial COX-2 an intriguing target for studies of healthy brain aging and neurodegenerative diseases. Research in aging mice found that (i) prostaglandin signaling impacts the metabolic state of microglia in aging mice, and (ii) ablation of the EP2 receptor of PGE_2_ not only restores metabolic phenotypes in these cells, but also reduces the expression of inflammatory cytokines, increases synaptic protein levels, and restores cognitive ability.^25^ Adding to the complexity, research indicates that COX-2 is expressed constitutively in neurons in the healthy brain where it has been suggested to play a role in some synaptic functions;^26−28^ additionally, neuronal COX-2 can also be elevated in some instances of inflammation and neurodegeneration.^29,30^ Although the connection of COX-2 with inflammation and neurodegeneration is well-established, the impacts of COX-2 activity during inflammatory processes and of changing COX-2 expression during disease progression are still very unclear.^6,31^

COX inhibition (COXib), both of COX-1 and COX-2, has been associated with and evaluated clinically for treating various neurological and neurodegenerative diseases^32−34^ and psychiatric disorders, including schizophrenia and depression.^7,35−41^ Despite significant research efforts into COXib, the evidence of efficacy for COX inhibition in neurologic and psychiatric trials is still quite mixed,^42−45^ and the mechanisms underlying the outcomes of COX activity and COX inhibition remain opaque. This is a complex landscape, and research approaches are currently limited to preclinical models and post-mortem tissue analyses. Given potential species differences in COX-2 brain function and pathology^30^ and the exceptional complexity of COX-2’s involvement in underlying pathological mechanisms, there is great need for tools that will enable in vivo research of COX-2 in the human brain.

To address this need, COX-2 positron emission tomography (PET) radiotracers for human neuroimaging have been pursued for several years.^30,46,47^ Imaging with PET allows researchers and clinicians to measure how critical targets, such as proteins or enzymes, change in the living brain at disease onset and throughout disease progression. More importantly, PET can measure how a target changes following therapeutic intervention, a critical step in assessing treatment efficacy. Recently, the PET radiotracer [^11^C]MC1, which is potent and selective for COX-2, was shown to be sufficient for measuring low-density basal expression of COX-2 in the human brain.^48^ However, the overall specific binding of the tracer is low (10%–20% of the total uptake). MC1, and other existing COX-2 inhibitors, such as celecoxib, have slow enzyme on-rates (*k*_on_), which can prove troublesome for achieving high binding signals in low expressing targets and act in practical terms as irreversible inhibitors (extremely low off rate, *k*_off_). Ideal CNS PET radiotracers engage the target quickly and demonstrate functionally reversible binding, allowing for appropriate scan length and proper kinetic modeling of PET data. Given that no fast-binding COX-2-targeting CNS PET radiotracers exist, we sought to develop fast-binding COX-2 inhibitors and then incorporate the appropriate radiolabeling handles for imaging, in this case, Carbon-11.

Here, we report the development of a COX-2 PET radiotracer that builds on the robust development process of [^11^C]MC1 but that we designed with a fundamentally different structure–activity approach. Selectivity for COX-2 over COX-1 has historically leveraged the differential association/dissociation kinetics related to a COX-2 conformational shift.^49^ In this instance, we developed and then applied a kinetically biased assay to identify COX-2 inhibitors that have faster association (less time-dependent inhibition) to determine the in vivo relationship between enzyme kinetics and PET radiotracer pharmacokinetics. Past research has reported interspecies variation in COX-2 inhibitors;^50^ therefore, after identifying and optimizing promising candidate tracers, we focused on animal models that expressed human COX-2. First, we drove expression of human COX-2 in rat brains using an adenoviral vector (AAV), thus allowing us to assess kinetic differences in vivo under high COX-2 expression, where enzyme kinetic differences would be most detectable. We then validated the tracer, [^11^C]BRD1158, in a second model of COX-2 overexpression, a human Thy-1-COX-2 transgenic mouse line.

In this study, we show that our novel COX-2 tracer [^11^C]BRD1158 displays high potency and selectivity toward its target. In vivo testing revealed appropriate target engagement and demonstrated that [^11^C]BRD1158 has ideal radiotracer properties related to its reversible fast binding kinetics.

Additionally, using mouse models of Huntington’s disease (HD) and post-mortem human brain tissue from donors with HD, we show that COX-2 protein levels increase in disease-affected brain regions and that increases are likely to be driven by increased expression in microglia specifically, suggesting that our tracer has potential utility as a clinical indicator for this disease indication.

## Results and Discussion

### Ligand Selection and Radiotracer Development

To select the best starting point for COX-2 radiotracer development, we extensively profiled a large set of known COX-2 selective inhibitors. Our primary focus was to identify a ligand with high binding affinity, COX-2 selectivity over COX-1, fast on-rate binding kinetics and improved free fraction in plasma to maximize the signal-to-noise ratio in PET imaging experiments. To favor compounds with high potency and fast on-rate kinetics, we utilized a liquid chromatography–mass spectrometry (LCMS)-based biochemical assay measuring the conversion of arachidonic acid to PGE2, optimized from Cao et al.,^50^ but with a short enzyme–inhibitor incubation time (2 min). To anticipate fast washout pharmacokinetics in vivo, we utilized simple plasma and brain protein binding assays to maximize the unbound fraction. After evaluating each scaffold for their COX-2 activity, Celecoxib, MC1, and Pyricoxib were the frontrunner candidates. Of these, MC1 possessed the greatest balance between potency, solubility, and fraction unbound (both in plasma and brain). Thus, MC1 emerged as the best starting point for medicinal chemistry efforts to develop a brain-permeable COX-2 radiotracer (Figure S1 in the Supporting Information).

We initially focused on the methyl sulfone-containing aromatic ring for optimization. As shown by other studies, the methyl sulfone imparts selectivity for COX-2 over COX-1^49,51^ and was left unchanged. Different aromatic rings (pyridyl isomers) improved solubility but decreased potency, while substitution saw a significant decrease in potency, so the simple phenyl methyl sulfone was maintained (data not shown). We turned next to explore the central pyrimidine ring of the molecule, envisioning that this ring would significantly impact both the solubility and potency of our compounds. Removal of the methoxy substituent had no significant impact (not shown); however, this coupled with converting the 2-amino pyrimidine to the 4-aminopyrimidine saw a significant improvement in solubility (15× from **1** to **2**) and *F*_up_ (∼2.5× from **1** to **2**; see Figure S2). The pyrazine ring **3** saw an almost 2-fold increase in *F*_up_, albeit at the cost of a decrease in solubility. Wondering whether the nitrogen between the phenyl ring and amine was essential, we constructed the benzene derivative **4**, which showed a marked improvement in potency (19 nM for **4**; see Figure S2). Converting to a pyridyl scaffold maintained this improvement, while boosting the lipophilic ligand efficiency (LLE). Ultimately, this 2,6-substituted pyridyl moiety was chosen (**5**, Figure S2) due to its balance of potency, improved LLE, and fraction unbound. With the core optimized, we turned to the molecule’s linker and distal aromatic ring.

**Figure 1:**
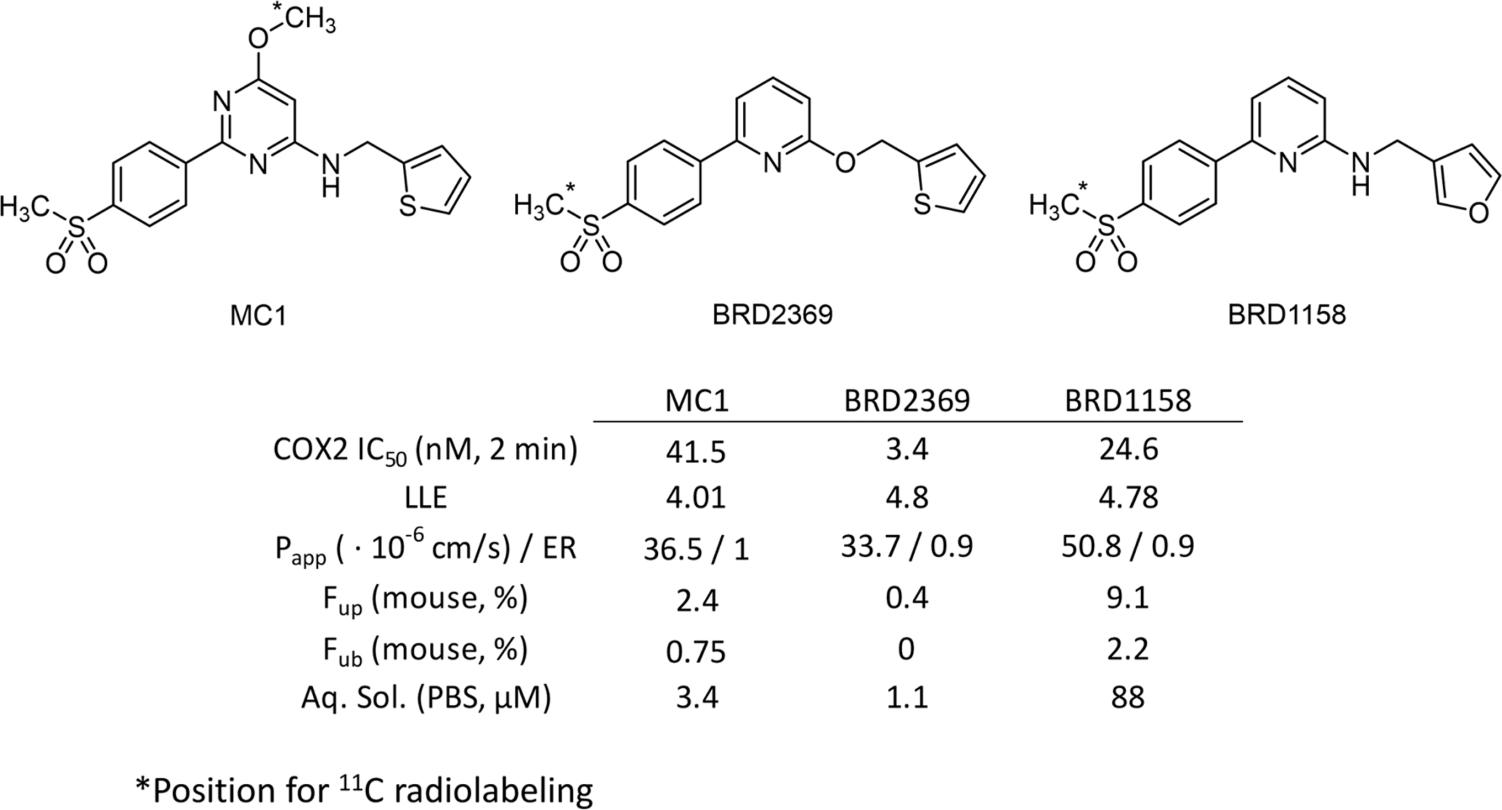
Selected COX-2 PET radiotracers with their in vitro potency and pertinent absorption, distribution, metabolism, and excretion (ADME) properties.

An obvious step in developing our structure–activity relationships (SAR) profile was to determine if the acidic N–H was essential to COX-2 activity. Substitution of the aryl amine to the aryl ether resulted in the most potent compound identified in the series, BRD2369 (see Figure 1, as well as Figure S3 in the Supporting Information). However, this came at a cost, in reduced fraction unbound and poor solubility. Other modifications such as tertiary amines and amides showed a significant to complete loss in potency (**6**–**8**; see Figure S3). We also explored carbon linkers and substitution of the benzylic position. However, these modifications came with a considerable decrease in potency (data not shown). After identifying the aryl amine and ether as optimal, we set out to explore SAR on the thiophene ring.

With thiophene rings being highly hydrophobic, we were eager to continue our expansion of the SAR to this portion of the molecule to identify more polar replacements, particularly to further increase the fraction unbound. We tested a variety of unsaturated and saturated heterocycles, and the large majority were significantly less potent than BRD2369 (data not shown). However, we did identify the 2- and 3-furyl rings as optimal to maintain high potency while significantly increasing the fraction unbound and LLE (**10** and **11**; see Figure S4 in the Supporting Information). Ultimately, combining the amine linker with a 3-furyl ring in BRD1158 (see Figure 1, as well as Figure S4) led to a potent COX-2 inhibitor with high fraction unbound in plasma and brain while maintaining desirable permeability with no P-gp efflux and high aqueous solubility.

Previous COX-2 discovery relied on whole blood assays to measure COX-2 inhibition. Focusing on fast-binding inhibitors, we prioritized SAR derived from a rapid inhibition LCMS-based enzymatic assay. Using short incubation times (2 min), we identified several fast-binding, potent COX-2 inhibitors. Most notably, BRD1158 and BRD2369 displayed remarkable potency (COX-2 IC_50_: 25 nM and 3 nM) compared to previously discovered MC1 (42 nM), celecoxib (54 nM), or rofecoxib (535 nM). Importantly, improvements in physicochemical properties (e.g., aqueous solubility, LLE, passive permeability) led to a 4-fold improvement in plasma protein binding (PPB) for BRD1158 (*F*_up_ = 0.091) compared to MC1 (*F*_up_ = 0.024).

### Rodent COX-2 PET Neuroimaging with Tracers [^11^C]BRD1158, [^11^C]BRD2369, and [^11^C]MC1

[^11^C]BRD1158 was initially assessed with in vivo PET neuroimaging of naïve rats, which confirmed rapid and robust brain uptake (Figure S5 in the Supporting Information). These initial scans demonstrated that endogenous COX-2 expression in naïve rats is below a detectable level for measuring specific binding, necessitating an overexpression model to evaluate the properties of [^11^C]BRD1158 compared to [^11^C]MC1. Additionally, while human and rodent COX-2 show more than 60% homology,^52^ variations at active site pockets can significantly impact ligand binding^49^ and are thus crucial to consider in radiotracer development, so our subsequent rodent models expressed the human COX-2 ortholog.

**Figure 2:**
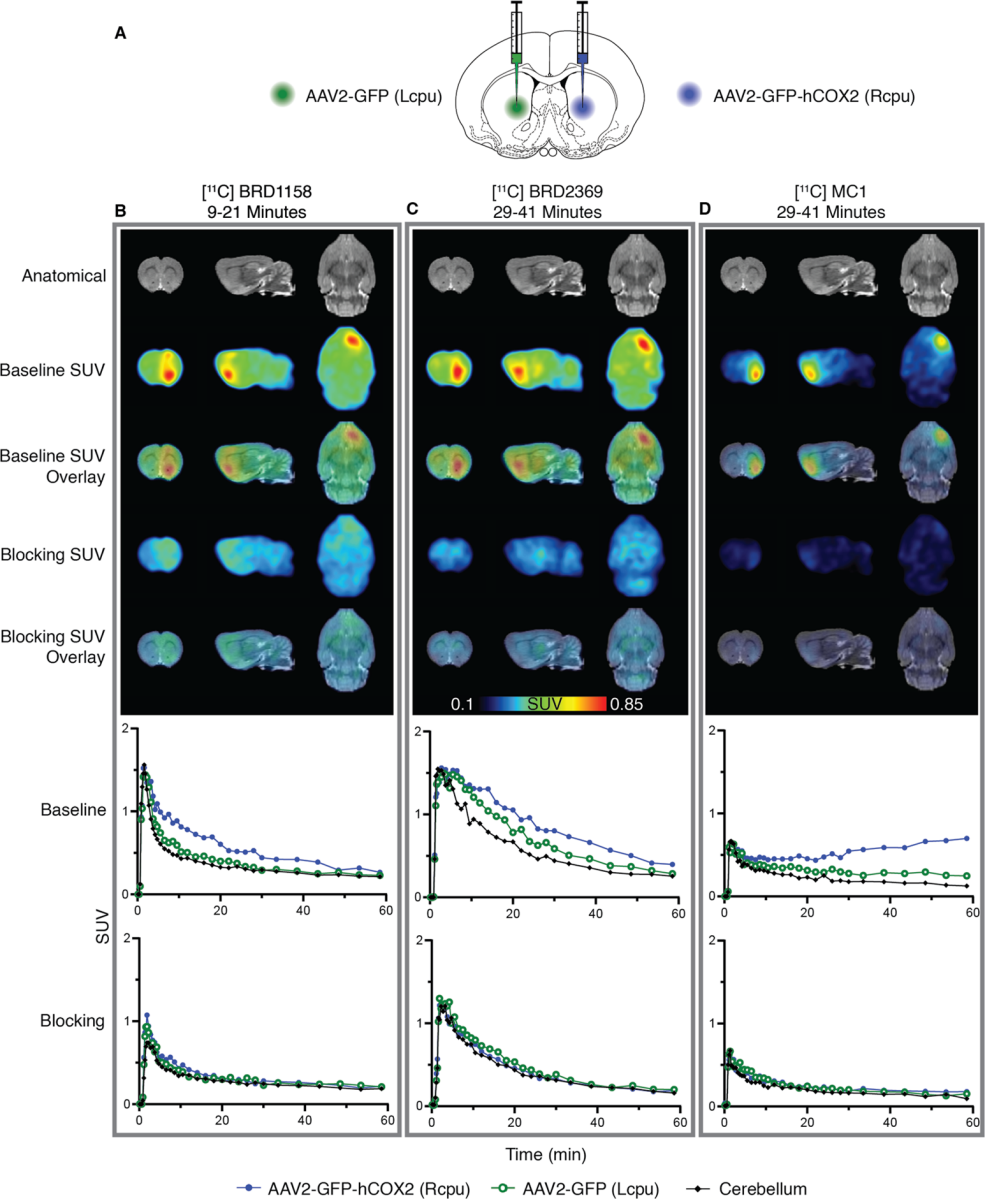
Brain PET intrasubject comparison of localized COX-2 overexpression (intrastriatal AAV2-GFP-hCOX2) with three different COX-2 radiotracers in one rat (animal ID: SD2006071) across three imaging sessions. [^11^C]BRD1158 is an effective COX-2 PET radiotracer that demonstrates uniquely fast onset in a rodent COX-2 overexpression model. (A) Schematic of injection paradigm in rats showing ICV injection of AAV2-GFP-hCOX2 (6.53 × 10^12^gc/mL) to right caudate (in blue), to induce overexpression of COX-2 and AAV2-GFP to left caudate (in green), as control. Animals were also given IP mannitol (10 mL/kg dose) to enhance transgene expression and increase vector spread. (B–D) Time average SUV images (above) and regional time activity curves (below) in one rat using tracer [^11^C]BRD1158 at 57 days post-AAV injection (left, B), [^11^C]BRD2369 at 61 days post-AAV injection (middle, C), and [^11^C]MC1 at 75 days post-AAV injection (right, D), at baseline and celecoxib (1 mg/kg) blocked conditions. Time averages shown are from the optimal portion of the scan; 9–21 min for [11C]BRD1158, and 29–41 min for [^11^C]BRD2369 and [^11^C]MC1. Representative MRs are shown. (See Figure S8 for a technical replicate in a second rat.)

Localized overexpression of human COX-2 was induced in rats with intrastriatal injections of AAV2-GFP-hCOX2 to the right caudate and AAV2-GFP (control virus) to the left caudate (Figure 2A).^53^ Animals also received a systemic injection of mannitol to enhance vector spread and transgene expression.^54^ To directly compare the kinetic profiles of the three COX-2 tracers, intrasubject imaging was performed in several rats. Each animal was scanned under baseline and competition (+celecoxib) conditions, testing each tracer within each animal (initial data in Figure 2). This design allowed us to keep the expression level as a constant in all comparisons. The full experimental design was then replicated (Figure S8 in the Supporting Information). An additional experiment lacking the control virus to the left caudate was performed to facilitate assessment of tracer uptake that could be mediated by the instrastiatal injection through blood brain barrier disruption (Figure S9 in the Supporting Information). We hypothesized that [^11^C]BRD1158 would demonstrate more favorable kinetics, including fast binding, relative to the other tracers.

In vivo PET studies in the COX-2 overexpression rat model showed that [^11^C]BRD1158 (Figure 2B) is a potent tracer with preferable kinetics, compared to [^11^C]BRD2369 (Figure 2C) and [^11^C]MC1 (Figure 2D). [^11^C]BRD1158 is fast to engage with the target and fast to washout with clear separation in the right caudate PET signal from controls (left caudate and cerebellum) seen as early as 10 min (Table S1 in the Supporting Information), compared to 30 min for the other tracers. In contrast, accumulation of [^11^C]MC1 at later time points, as seen in Figure 2D, Figures S8D and S9, indicates functionally irreversible binding or the accumulation of a radioactive metabolite.

**Figure 3:**
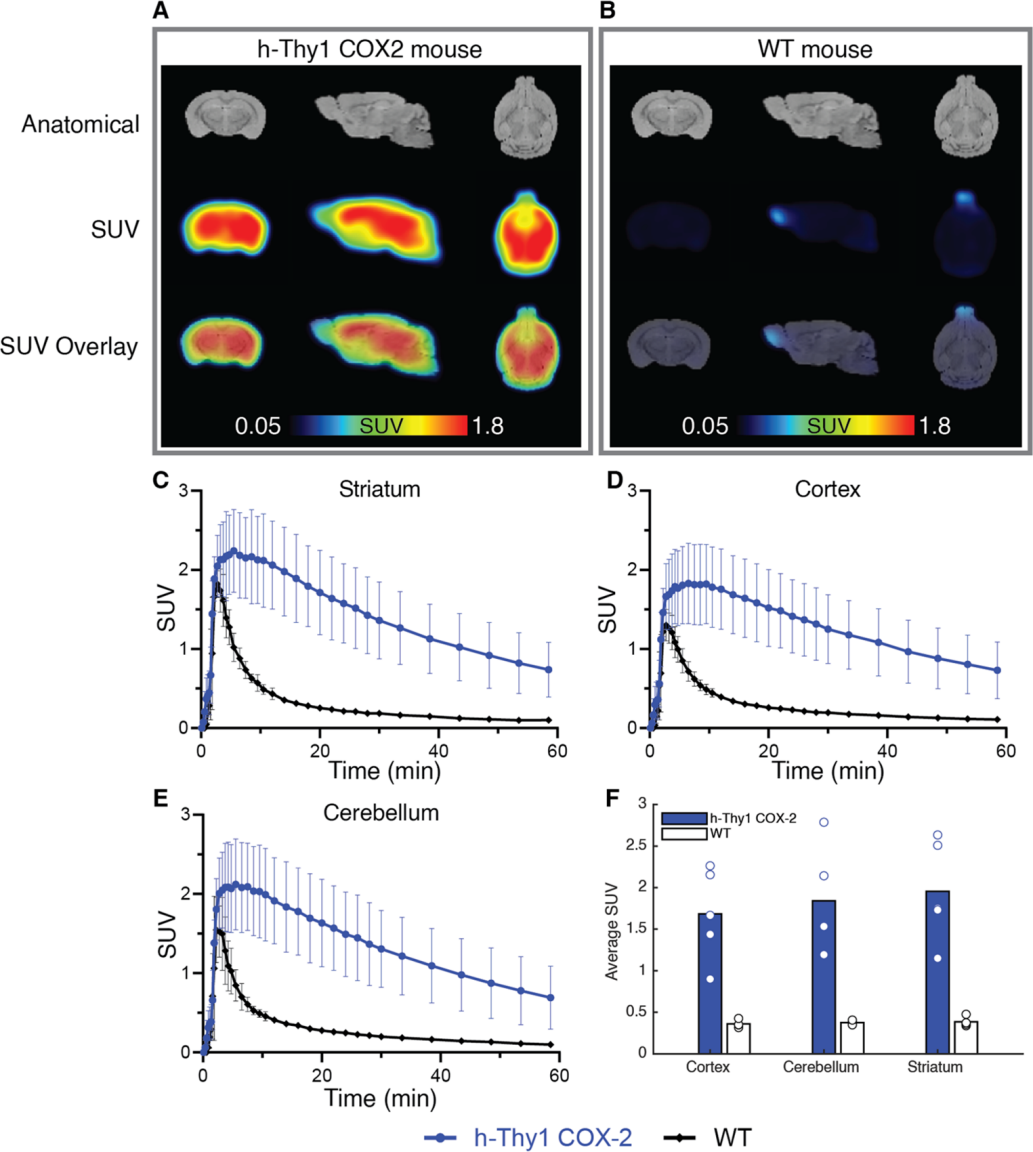
[^11^C]BRD1158 shows efficacy in PET imaging in a h-Thy1 COX-2 transgenic mouse line. Time average SUV images with (A) [^11^C]BRD1158 in one hCOX-2 mouse, compared to (B) one wild-type mouse. hCOX-2 versus wild-type mouse time activity curves from the (C) striatum, (D) cortex, and (E) cerebellum. (F) Cohort average SUVs for hCOX-2 (*n* = 5) versus wild-type (*n* = 4) mice in the cortex, cerebellum, and striatum. Representative MRs are shown.

We performed additional validation of [^11^C]BRD1158 through in vivo PET imaging of an hCOX-2 transgenic mouse line (Figure 3), which overexpressed human COX-2 primarily in neurons (Strain No. 010703, Jackson Laboratories). The hCOX-2 mice offered a COX-2 overexpression model that did not necessitate intracranial injections. While we did perform contralateral sham (control virus) injections in the AAV rats to assess potential injection confounds, as COX-2 is part of the injury and inflammation response, it was important to validate in a model naïve to brain insult to mitigate how aspects of the AAV rat experimental design could have misled our interpretations of tracer specific binding. We found that [^11^C]BRD1158 was also effective for imaging in the hCOX-2 transgenic mice and that [^11^C]BRD1158 continued to demonstrate early target-engagement, achieving significant signal differentiation from wild-type controls within 10–20 min (Figure 3 and Table 1).

**Table 1:** SUV Average (9–21 min) for h-Thy1 COX-2 versus Wild-Type Mice Using Tracer [^11^C]BRD1158

ROI	h-Thy1 COX-2 (*n* = 5)	WT (*n* = 4)	*P* value
cortex	1.68 ± 0.50	0.359 ± 0.04	0.0058
cerebellum	1.84 ± 0.56	0.376 ± 0.02	0.0065
striatum	1.95 ± 0.55	0.385 ± 0.05	0.0044

Taken together, the data from rodent imaging demonstrate that [^11^C]BRD1158 has features appropriate to advance for human PET neuroimaging that are differentiated from [^11^C]MC1, namely, the early fast target-engagement and functional reversibility.

### Microglial COX-2 Upregulation in Huntington’s Disease

In preparation for human imaging studies, we also used rodent models and post-mortem tissue in Huntington’s Disease to explore a potential clinical use for COX-2 imaging in neurodegeneration as we move toward human translation. Although many PET radioligands have been developed over the years to act as staging and progression markers for neurodegenerative diseases, including HD, no PET ligands have been able to reliably predict disease conversion or aid in the stratification of patients for clinical trials in HD. We believe PET imaging with [^11^C]BRD1158 has the potential to be an objective clinical marker that can assess, and lead to the discovery of, effective disease-modifying therapeutics for HD patients.

**Figure 4:**
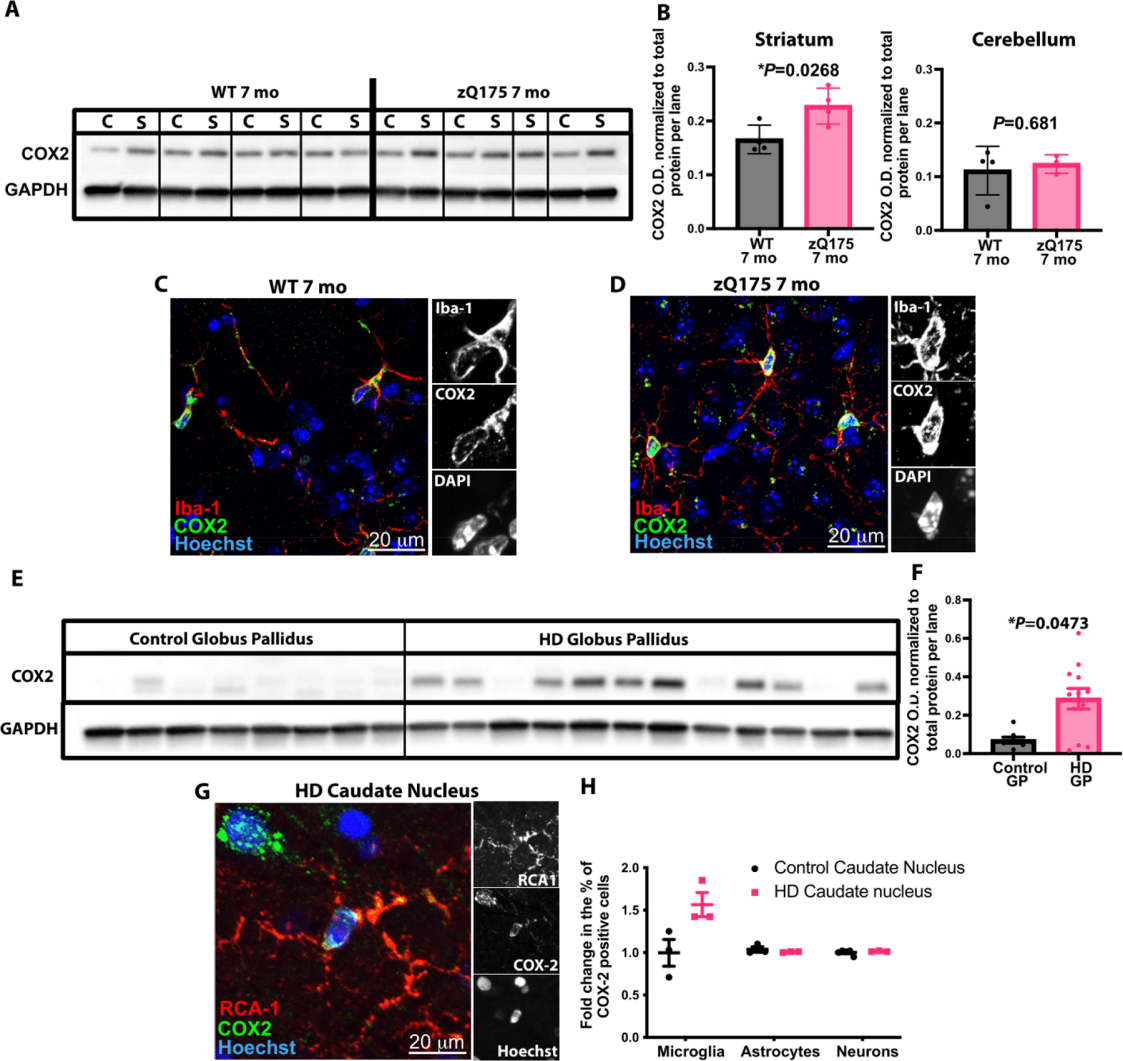
Increased COX-2 levels in Huntington’s Disease are partly driven by elevated expression in microglia. (A) Representative Immunoblot showing staining for COX-2 and GAPDH in striatal and cerebellar extracts from 7 mo zQ175 mice and WT littermates. (B) Quantification of COX-2 levels in 7 mo zQ175 mice and WT littermates. Band intensity is normalized to total protein per lane (measured using BioRad Stain-Free gel (see Figure S10C); *n* = 4 zQ175 mice and 4 WT littermate controls. Unpaired *t*-test: (C) for striatum, (*) *P* = 0.0268 and (D) for cerebellum, *P* = 0.6810. Representative IHC images showing staining for COX-2 and Iba-1 in the dorsolateral striatum of a 7 mo zQ175 mice and a WT littermate. Scale bar = 20 μm. Insets show single channel images of the soma of a cell. (E) Representative immunoblot showing staining for COX-2 and GAPDH in globus pallidus extracts from HD patients and age-matched controls (see Figure S10D and S10E in the Supporting Information). (F) Quantification of COX-2 levels in tissue from HD patients and controls. Band intensity is normalized to total protein per lane (measured using BioRad Stain-Free gel (Figure S11); *n* = 12 samples from HD patients and *n* = 8 samples from age-matched control individuals. Unpaired *t*-test, (*) *P* = 0.0473. (G) Representative IHC image of the caudate nucleus of a HD patient (Vonsattel grade 2), showing COX-2 staining in RCA1 positive microglia. Insets show single channel images; scale bar = 20 μm. (H) Bar chart showing quantification of the percentage of microglia (RCA1 + ve), astrocytes (sox9 + ve) and neurons (NeuN + ve) in the caudate nucleus of Vonsattel grade 2 HD tissue, as well as tissue from the same region of age-matched controls that stained + ve for COX-2; *n* = 3 samples from HD patients and *n* = 3 samples from age-matched controls. Two-way analysis of variance (ANOVA), with clinical designation as a source of variation: (*) *P* = 0.0127; with cell type as a source of variation, (**) *P* = 0.0062; with the interaction of clinical designation and cell type as a source of variation, (**) *P* = 0.0036. Sidak’s posthoc test for multiple comparisons, for microglia, (***) *P* = 0.0007, for astrocytes *P* = 0.9918 and for neurons *P* = 0.9990.

To determine whether COX-2 levels might be elevated in neurodegenerative contexts, we decided to interrogate the zQ175 mouse model of Huntington’s disease. This mouse is thought to capture some of the earliest pathological events occurring in the presymptomatic phase of HD with synaptic dysfunction and the selective loss of corticostriatal synapses beginning at three months of age—a time point that also coincides with the appearance of visual discrimination learning deficits.^55^ Both phenotypes have also recently been observed in presymptomatic HD patients.^56−58^ We initially performed immunoblotting using extracts from this mouse from a disease-affected brain region (striatum) and a region that is relatively spared (cerebellum). These results showed that COX-2 was selectively increased in striatal extracts from the zQ175 mice, relative to wild-type (WT) littermates, but a comparable increase was not observed in extracts from the less disease-affected cerebellum (see Figures 4A and 4B, as well as Figures S10 A, B, C).

Interestingly, we found that COX-2 colocalizes largely with Iba-1 positive microglia in the mouse striatum, suggesting that the increase we observed may be driven primarily by elevated expression in this cell type (Figures 4C and 4D). To test this and to see if the elevation of COX-2 was also observed in human tissue, we performed immunoblot analysis on globus pallidus extracts dissected from HD post-mortem tissue and tissue from age-matched controls. These data showed that COX-2 levels were significantly elevated in the HD tissue, relative to levels seen in extracts from the same region obtained from control age-matched individuals (see Figures 4E and 4F, as well as Figures S10D–F). When we subsequently used IHC and cell-type-specific markers to identify which cells were contributing to the increased expression of COX-2, we saw that, in the HD tissue, there was an increase in the proportion of microglia (delineated by RCA1 staining) that stained positive for COX-2 with no change in the percentage of COX-2 positive astrocytes or neurons in this brain region (see Figures 4G and 4H, as well as Figures S11A and S11B in the Supporting Information).

Taken together, these results show that (i) COX-2 is elevated in disease-relevant regions of a mouse model of presymptomatic HD and (ii) this increase in COX-2 levels can also be observed in post-mortem tissues from HD patients, where it appears to be mainly due to elevated expression in microglia. To develop effective therapeutics for HD, we must understand the biological changes in the living brain that occur at the earliest evidence of disease conversion. Our data indicate that COX-2 holds promise as a novel clinical marker of HD and it will be important to determine if, in conjunction with other biomarkers, it could be used to help predict disease onset and progression.

Translation of [^11^C]BRD1158, which is a highly specific, brain penetrant COX-2 PET radiotracer, and parallel studies characterizing the mechanism through which COX-2 influences synaptic pathology will (i) enable the study of COX-2 expression changes and distribution in the living brain to monitor and characterize key pathophysiological events in HD and potentially any microglial dynamics that might be important in synaptic elimination mechanisms;^55^ (ii) allow for patient stratification in novel brain-permeable COX-2 inhibitor clinical trials; (iii) provide a therapeutic imaging biomarker for monitoring response to new treatment strategies; and (iv) generate a translatable tool to evaluate the role of COX-2 in HD and potentially other neurodegenerative diseases in the living human brain.

## Conclusion

In this study, we have characterized the development of a novel COX-2 CNS PET radiotracer, [^11^C]BRD1158, which has the potential to be a valuable asset in neurodegenerative research, due to its unique properties. With [^11^C]BRD1158, we achieved a high-potency, functionally reversible radiotracer with fast kinetic binding, which proved well-suited for future translation into clinical imaging through evaluations in rodent models overexpressing human COX-2. Data from animal models and post-mortem tissue in Huntington’s disease (HD) suggest that this condition could serve as a strategic starting point for the translation of [^11^C]BRD1158 into human imaging. Our studies are not without notable limitations, including uncertainty on the required sensitivity for detecting COX-2 signal in the healthy human brain and across stages of disease conversion and progression, as well as the clinical implication of changes in COX-2 levels across disease states. However, we believe these lingering questions are best answered in a human imaging context. We are excited to move forward with the translation of [^11^C]BRD1158 and explore its potential to open new avenues for understanding and treating HD and other neurodegenerative and psychiatric disorders.

## Supporting Information Available

Safety statement, general chemistry methods, chemical synthesis, analytical data, radiochemical data, radiolabeling, plasmid map design of AAV-GFP-hCOX2 construct, details on animal models and imaging experiments, extended data from rodent experiments, immunoblotting and immunohistochemistry (PDF)
