# Supplementary material for: A Fast-Binding, Functionally Reversible, COX-2 Radiotracer for CNS PET Imaging
Source: ACS Cent Sci. 2024 Apr 25;10(5):1105–14. doi: 10.1021/acscentsci.3c01564 (PMC11117721; doi:10.1021/acscentsci.3c01564)
Supplement: Supplementary file 1 — oc3c01564_si_001.pdf [file oc3c01564_si_001.pdf]

Supporting Information for:

A Fast-binding, Functionally Reversible, COX-2 Radiotracer for CNS PET Imaging

Michael S. Placzek, Daniel K. Wilton, Michel Weïwer, Mariah A. Manter, Sarah E. Reid, Christopher J. Meyer, Arthur J. Campbell, Besnik Bajrami, Antoine Bigot, Sarah Bricault, Agathe Fayet, Arnaud Frouin, Frederick Gergits, Mehak Gupta, Wei Jiang, Michelle Melanson, Chiara D. Romano, Misha M. Riley, Jessica M. Wang, Hsiao-Ying Wey, Florence F. Wagner, Beth Stevens, Jacob M. Hooker

Table of Contents

SUPPLEMENTAL FIGURES……1-10

SI Figure 1……1
SI Figure 2……2
SI Figure 3……3
SI Figure 4……4
SI Figure 5……5
SI Figure 6……6
SI Figure 7……7
SI Figure 8……8
SI Figure 9……9
SI Table 1……10
SI Figure 10……11
SI Figure 11……12

EXPERIMENTAL METHODS……13-45

Safety Statement……13
General Chemistry Methods……13
Synthesis of COX-2 Analogues **1, 2, 4, 6** and **7**……13-18
Analytical Data……19-32
Synthesis of precursors and [^{11}C] radiolabeled **MC1**, **BRD2369**, and **BRD1158**……33-41
Animals……42
Animal Preparation……42
AAV Rat Experimental Groups, Drug Formulation and Surgical Procedures……42-43
Rodent PET/MRI Acquisition……43
Rodent Image Analysis……43-44
Immunoblotting……44
Immunohistochemistry on rodent tissue sections……44-45
Immunohistochemistry using postmortem human tissue sections……45

SUPPORTING INFORMATION REFERENCES……46

SUPPLEMENTAL FIGURES

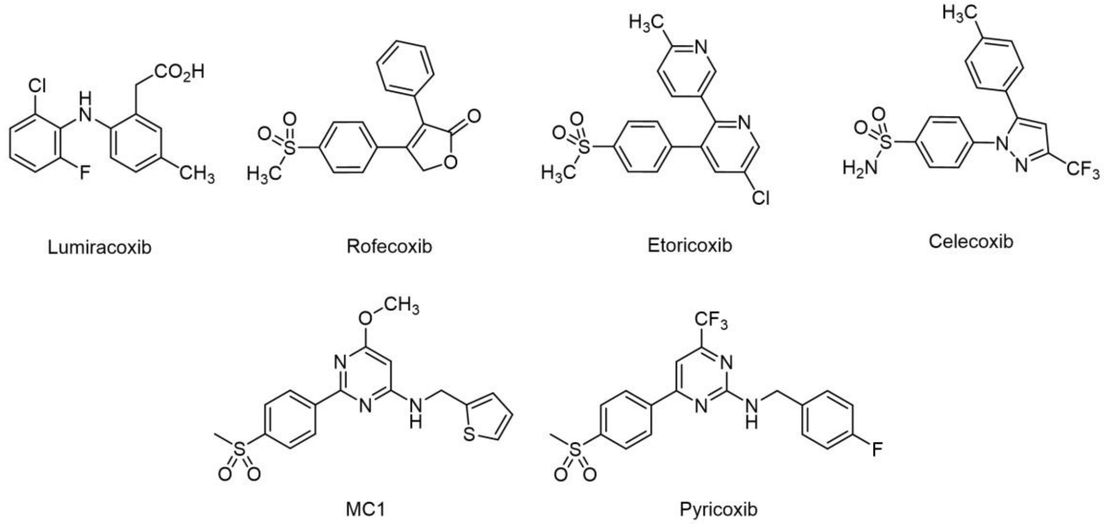

	Luminarcoxib	Roficoxib	Etoricoxib	Celecoxib	MC1	Pyricoxib
COX2 IC_{50} (nM, 2 min)	199	535	2,870	54.3	41.5	24.8
COX2 IC_{50} (nM, 10 min)	2,590	74.3	333	9.49	6.08	3.08
LLE	3.13	3.26	2.34	3.51	4.01	3.69
P_{app} (· 10^{-6} cm/s) / ER	72.2 / 0.9	16.8 / 1.3	14.3 / 3.1	ND	36.5 / 1	ND
F_{up} (mouse, %)	0.57	14.75	15.59	0.31	2.4	0.239
F_{ub} (mouse, %)	2.8	10.8	7.9	0.2	0.75	0
Aq. Sol. (PBS, µM)	500	25	159	2.5	3.4	0.65

SI Figure 1. Extensive profiling of known COX-2 inhibitors identified MC1 as the preferred candidate for PET tracer optimization.

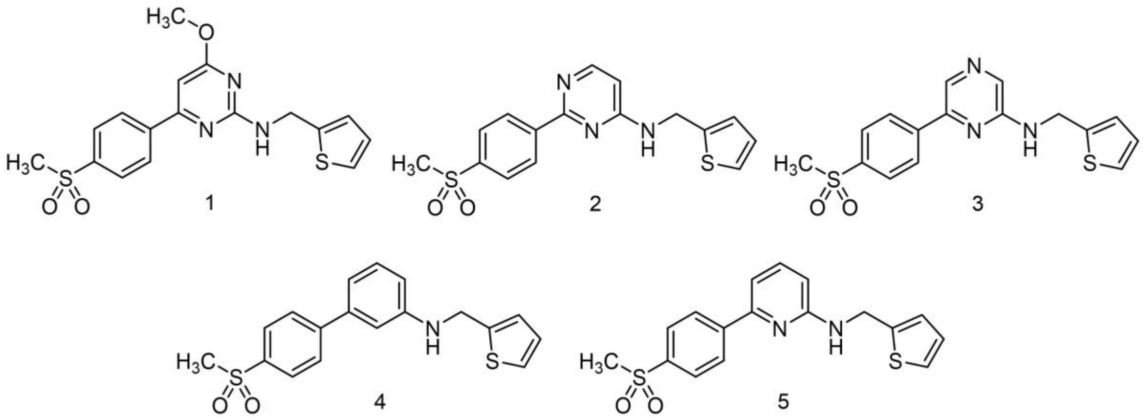

	1	2	3	4	5
COX2 IC_{50} (nM, 2 min)	62	63.5	61.6	19.5	12
LLE	3.9	4.2	5.2	3.5	4.5
P_{app} ($\cdot$ 10^{-6} cm/s) / ER	ND	ND	49.7 / 1.6	ND	ND
F_{up} (mouse, %)	1.5	3.8	7	ND	1.6
F_{ub} (mouse, %)	ND	ND	2	ND	ND
Aq. Sol. (PBS, µM)	2.1	33	11	ND	2.1

SI Figure 2. Systematic SAR of the central ring identified the 2-pyridyl ring as the best scaffold to pursue.

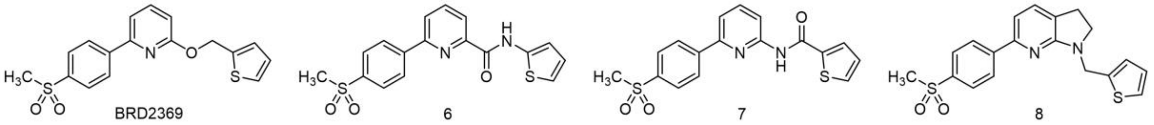

	BRD2369	6	7	8
COX2 IC_{50} (nM, 2 min)	3.4	> 5,000	> 5,000	124
LLE	4.8	2.3	2.08	2.9
P_{app} ($\cdot$ 10^{-6} cm/s) / ER	33.7 / 0.9	ND / ND	ND / ND	ND / ND
F_{up} (mouse, %)	0.4	ND	ND	ND
F_{ub} (mouse, %)	0	ND	ND	ND
Aq. Sol. (PBS, µM)	1.1	ND	ND	ND

SI Figure 3. Summary of the SAR around the linker portion identified aryl ethers as the most potent COX-2 inhibitors.

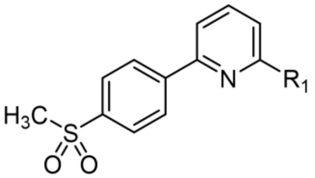

	9	10	11	12	BRD1158
R_1	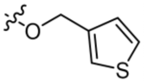	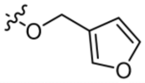	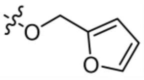	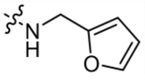	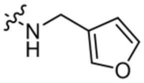
COX2 IC_{50} (nM, 2 min)	8.9	8.7	12.5	17.5	24.6
LLE	4.3	4.9	4.7	5	4.78
P_{app} (· 10^{-6} cm/s) / ER	ND / ND	ND / ND	ND / ND	68.5 / 1	50.8 / 0.9
F_{up} (mouse, %)	ND	1.1	ND	11.8	9.1
F_{ub} (mouse, %)	ND	ND	ND	1.9	2.2
Aq. Sol. (PBS, μM)	3.7	10	ND	27	88

SI Figure 4. Optimization of the distal aryl ring identified the 3-furyl-methylamine as the best balance between potency and ADME properties.

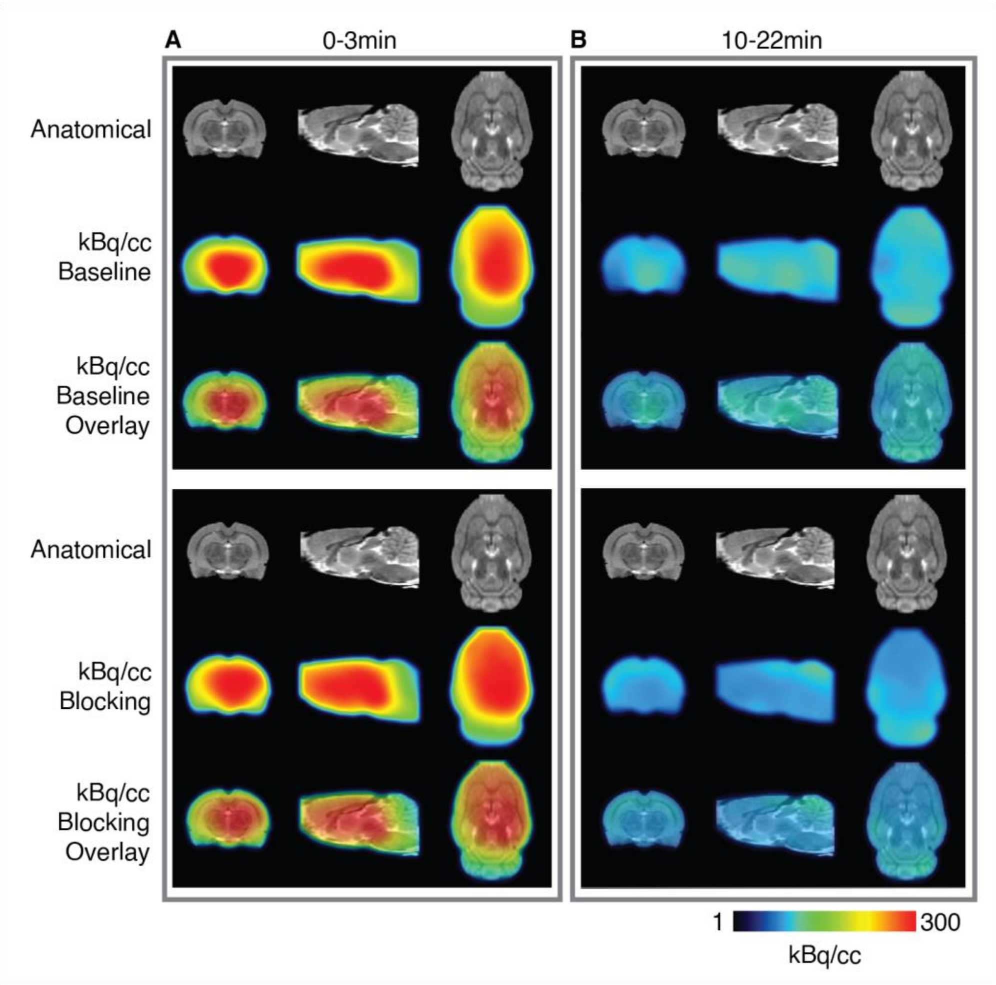

SI Figure 5. One naïve rat underwent MR and PET imaging using tracer [^{11}C]BRD1158. Time average PET activity images demonstrate that (**A**) [^{11}C]BRD1158 has good brain uptake and (**B**) [^{11}C]BRD1158 shows low endogenous COX-2 levels in naive rats. Representative MRs are shown.

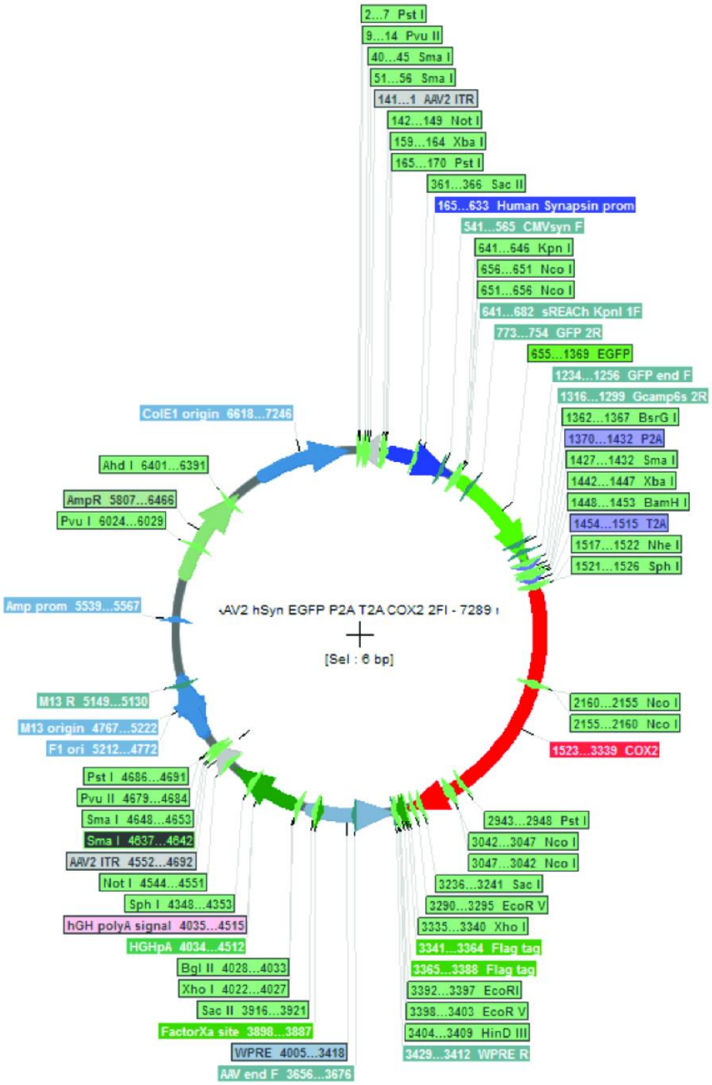

SI Figure 6. Plasmid map showing the sequence of the AAV2-GFP-hCOX2 construct. To generate this a human COX2-2 flag expression construct was obtained from Addgene (102489) and the gene sequence excised and subcloned into a pAAV2 backbone containing a human synapsin promoter. The gene was inserted alongside a GFP expression cassette with the two genes separated by a P2A linker sequence. The plasmid was subsequently packaged into an AAV.

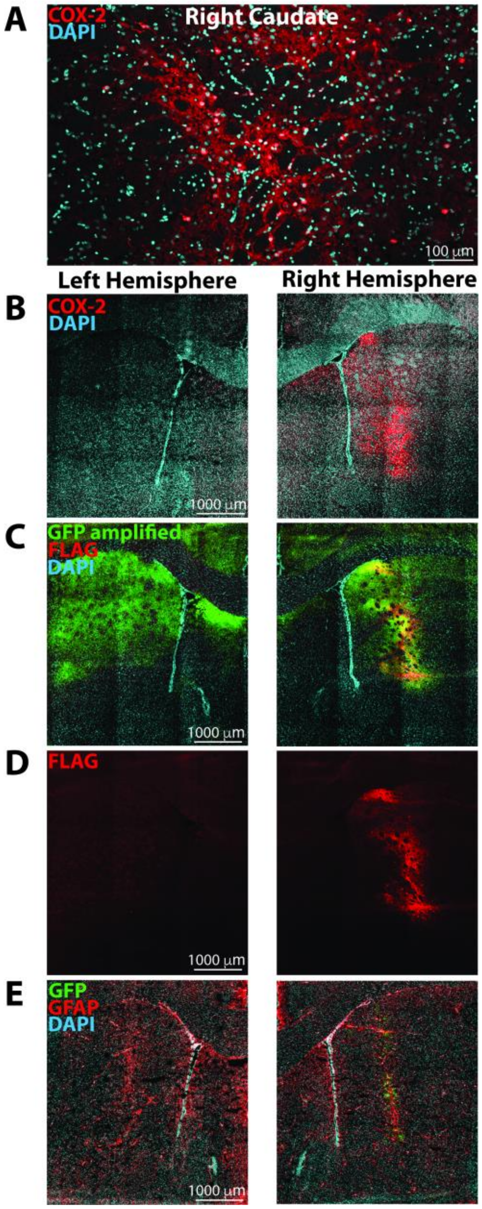

SI Figure 7. Expression of COX-2 in the rat brain following intracerebral injection of the AAV2-GFP-hCOX2 (6.53E+12gc/ml) across 3 depths in the right caudate and AAV2-GFP across 3 depths in the left caudate as control. (**A**) Fluorescence photomicrographs showing COX-2 staining in the right caudate of a rat following injection of the AAV2-GFP-hCOX2 construct (6.53E+12gc/ml) into the right caudate and the AAV2-GFP construct (>3E+12vg/ml) into the left caudate. (**B,C,D,E**) Lower magnification images showing sections of the left and right hemispheres of the brain from the same animal stained with COX-2 (**B**); GFP (amplified) and FLAG (**C**); FLAG (**D**); GFP and GFAP (**E**).

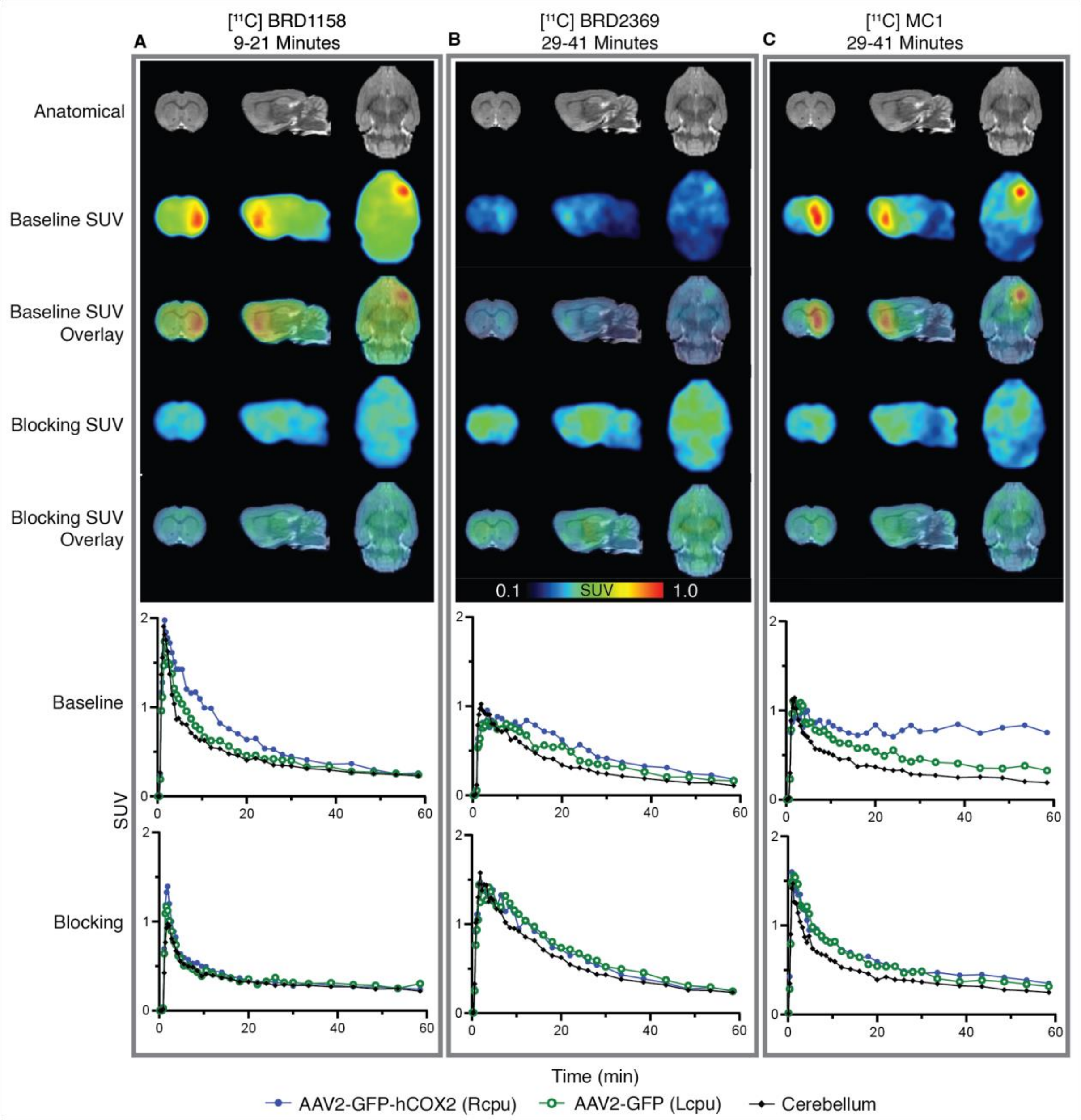

SI Figure 8. Technical replicate of Figure 2, brain PET intrasubject comparison study of localized COX-2 overexpression (intrastriatal AAV2-GFP-hCOX2) with three different COX-2 radiotracers in one rat (animal id: SD2006072) across three imaging sessions. As described in Figure 2, a second rat received an ICV injection of AAV2-GFP-hCOX2 (6.53E+12gc/ml) to right caudate (in blue), to induce overexpression of COX-2 and AAV2-GFP to left caudate (in green), as control. Animal also received IP mannitol (10 mL/kg dose) to enhance transgene expression and increase vector spread. (**A-C**) Time average SUV images (above) and regional time activity curves (below) in one rat using tracer [^{11}C]BRD1158 at 57 days post injection (left, **A**), [^{11}C]BRD2369 at 61 days post injection (middle, **B**) and [^{11}C]MC1 at 75 days post injection (right, **C**), at baseline and celecoxib (1mg/kg) blocked conditions. Time averages shown are from the optimal portion of the scan; 9-21 minutes for [^{11}C]BRD1158, and 29-41 minutes for [^{11}C]BRD2369 and [^{11}C]MC1. Representative MRs are shown.

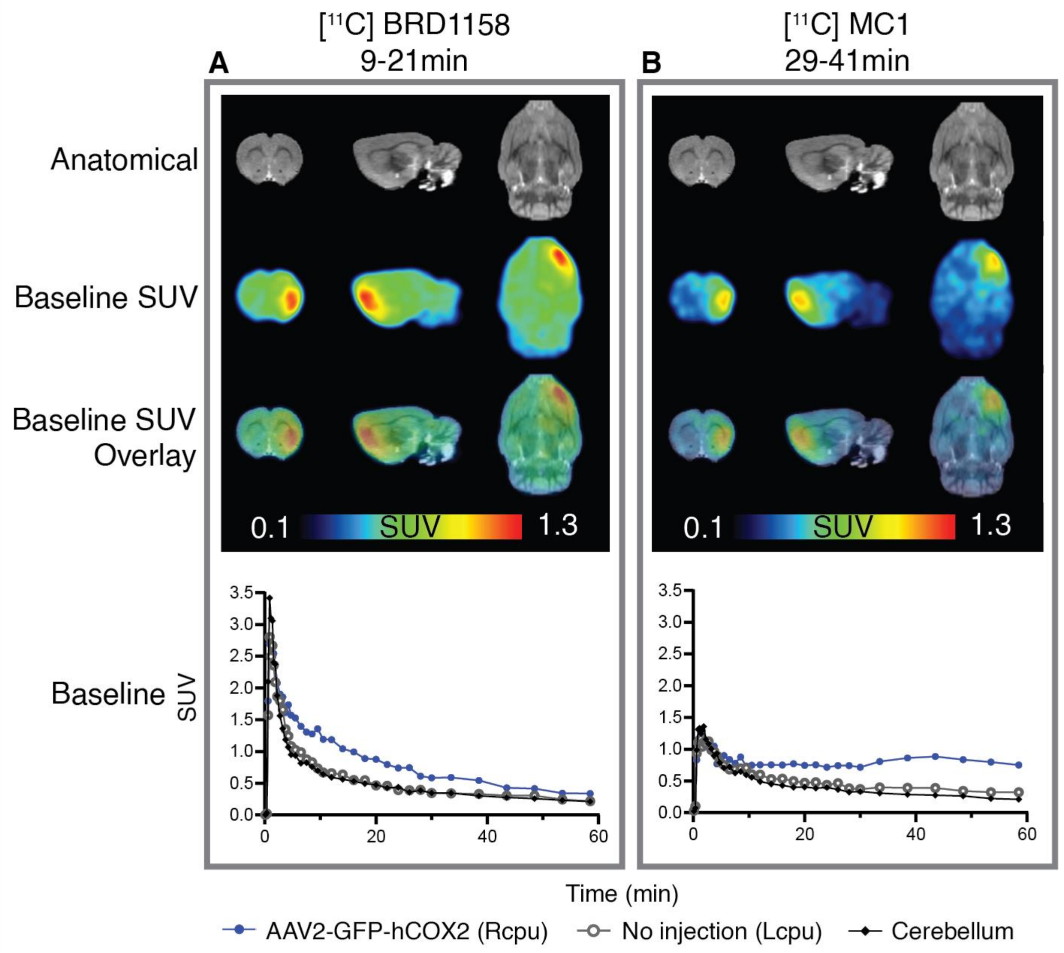

SI Figure 9. Alternative experimental setup for brain PET intrasubject comparison study of localized COX-2 overexpression (intrastriatal AAV2-GFP-hCOX2) with two different COX-2 radiotracers in one rat (animal id: SD2006080) across two imaging sessions. Animal received an ICV injection of AAV-GFP-COX-2 (6.53E+12gc/ml) to right caudate, to induce overexpression of COX-2, but differently from setup previously described in Figure 4 and SI Figure 8, there was no sham injection for the left caudate. Animal was also given IP mannitol (10 mL/kg dose) to enhance transgene expression and increase vector spread. (**A,B**) Baseline time average SUV images (above) and regional time activity curves (below) in one rat using tracer [^{11}C]BRD1158 (left, **A**) and [^{11}C]MC1 (right, **B**), at 36 days post injection. Time averages shown are from the optimal portion of the scan; 9-21 minutes for [^{11}C]BRD1158 and 29-41 minutes for [^{11}C]MC1. Representative MRs are shown.

SI Table 1. SUV time averages from early portion of scan (9-21 minutes) for AAV2-COX2 rats (animal ID: SD2006071 and SD2007072) for tracers [^{11}C]BRD1158, [^{11}C]BRD2369 and [^{11}C]MC1.

	No Injection (Cerebellum)	AAV2-GFP (Left Caudate Putamen)	AAV2-GFP-hCOX2 (Right Caudate Putamen)
animal ID: SD2006071, Figure 2			
**[^{11}C]BRD1158**			
Baseline	0.4003	0.4779	0.7406
Block	0.3177	0.3392	0.3838
**[^{11}C]BRD2369**			
Baseline	0.7971	1.0541	1.2220
Block	0.5736	0.6829	0.6088
**[^{11}C]MC1**			
Baseline	0.2658	0.3477	0.4582
Block	0.2313	0.2751	0.2792
animal ID: SD2006072, SI Figure 8			
**[^{11}C]BRD1158**			
Baseline	0.5268	0.5976	0.8567
Block	0.3683	0.3842	0.4222
**[^{11}C]BRD2369**			
Baseline	0.4886	0.6165	0.7512
Block	0.7903	0.9568	0.9102
**[^{11}C]MC1**			
Baseline	0.4364	0.6242	0.7913
Block	0.5126	0.6802	0.7070

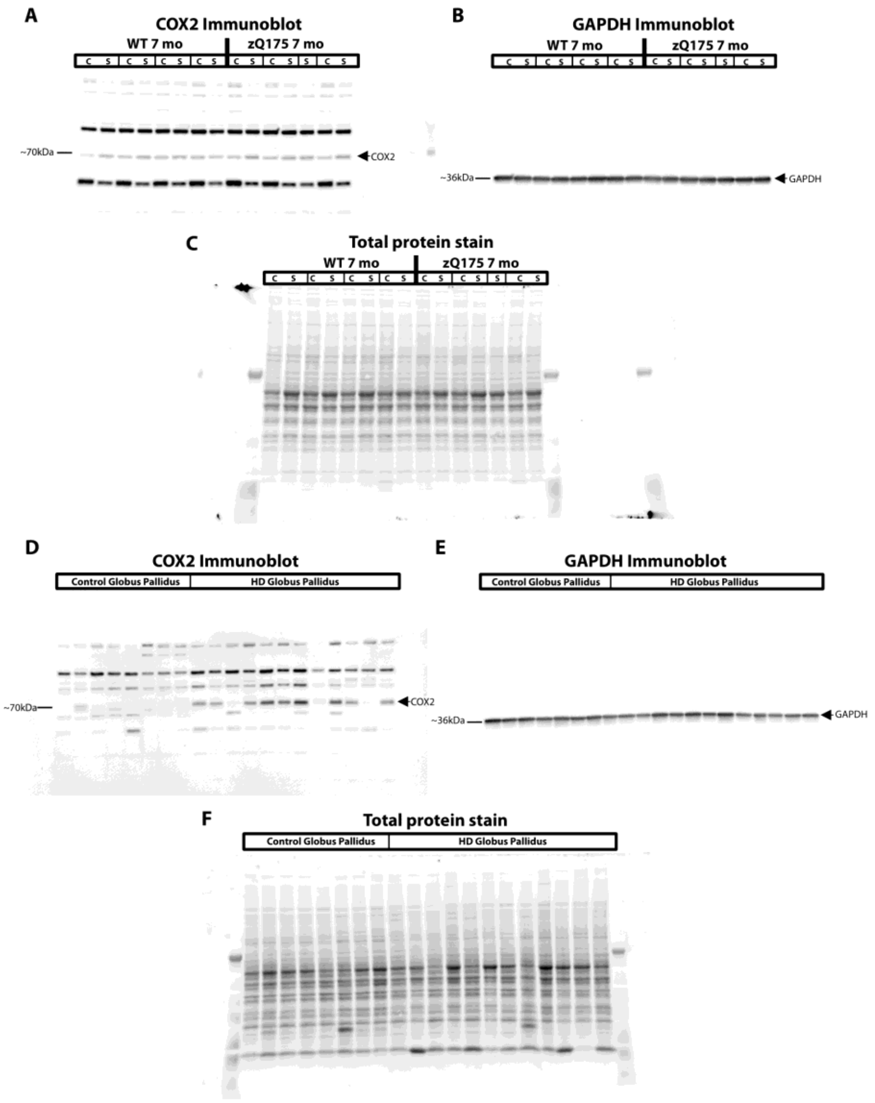

SI Figure 10. (**A**) Full length immunoblot showing staining for COX-2 in striatal (S) and cerebellar (C) extracts from 7 mo zQ175 mice and WT littermates; arrow indicates the position of the COX-2 band. (**B**) Full length immunoblot showing staining for GAPDH in striatal (S) and cerebellar (C) extracts from 7 mo zQ175 mice and WT littermates; arrow indicates the position of the GAPDH band. (**C**) Full length immunoblot showing total protein stain for striatal (S) and cerebellar (C) extracts from 7 mo zQ175 mice and WT littermates. (**D**) Full length immunoblot showing staining for COX-2 in postmortem extracts of globus pallidus tissue from HD patients and age matched control individuals (see methods and supplemental table 1 for clinical information); arrow indicates the position of the COX-2 band. (**E**) Full length immunoblot showing staining for GAPDH in postmortem extracts of globus pallidus tissue from HD patients and age matched control individuals; arrow indicates the position of the GAPDH band. (**F**) Full length immunoblot showing total protein stain for postmortem extracts of globus pallidus tissue from HD patients and age matched control individuals.

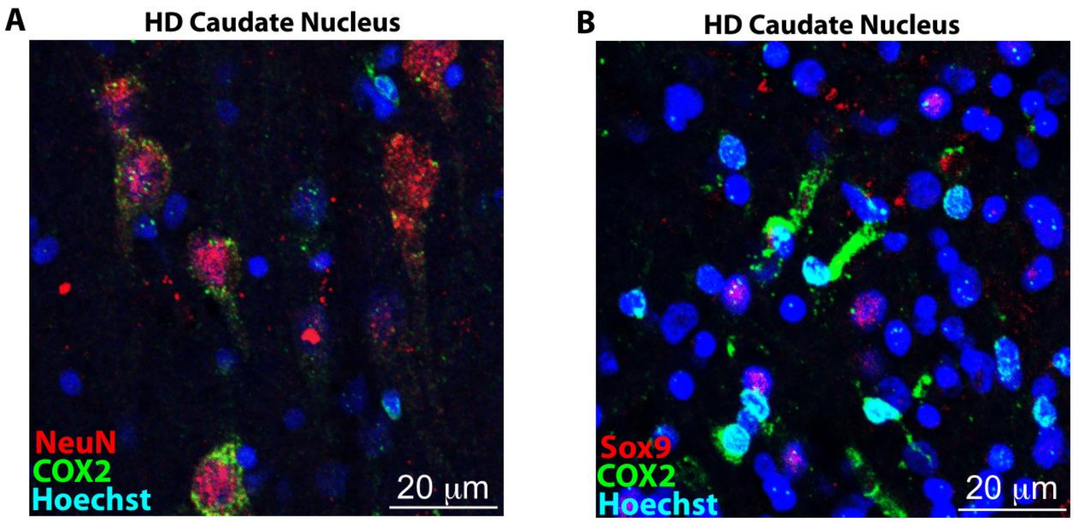

SI Figure 11. (**A**) Representative IHC image of the caudate nucleus of a HD patient (Vonsattel grade 2) showing COX-2 staining in NeuN positive neurons. Scale bar = 20 mm. (**B**) Representative IHC image of the caudate nucleus of a HD patient (Vonsattel grade 2) showing minimal COX-2 staining in sox9 positive astrocytes. Scale bar = 20 μm. Note the relatively minimal staining in this cell type.

EXPERIMENTAL METHODS

Safety Statement
No unexpected or serious safety concerns were encountered in these experimental procedures.

General Chemistry Methods
All dry reactions were carried out in oven-dried glassware. Reactions were carried out under an atmosphere of nitrogen or argon unless stated otherwise. Solvents were dried and distilled using classical procedures and stored on molecular sieves 4 Å (MilliporeSigma). Unless stated otherwise, solvents were removed by rotary evaporation under reduced pressure at 40°C. All other chemicals (Acros, Sigma-Aldrich, TCI, Carbosynth, Merck, Combi-blocks) were used as received. Nuclear magnetic resonance (1H NMR) spectra were recorded at room temperature, in $CDCl_3$, MeOD, DMSO-d_6 or D_2O (300 or 400 MHz). Chemical shifts (δ) are indicated in ppm and coupling constants (J) in Hz. ESI-MS were recorded on a LC/MSD trap. HRMS were performed on an orbitrap mass spectrometer using electrospray ionization. Thin-layer chromatography (TLC) was carried out using plates of silica gel 60 with fluorescent indicator and revealed with UV light (254 nm) when possible and Von's reagent. Flash chromatography was performed using silica gel 230-400 mesh.

Synthesis of COX-2 Analogues 1, 2, 4, 6 and 7
The synthesis of compounds **BRD2369**, **BRD1158**, **3**, **5**, and **8-12** are all reported in our previously disclosed patent[1].

Synthesis of **1**

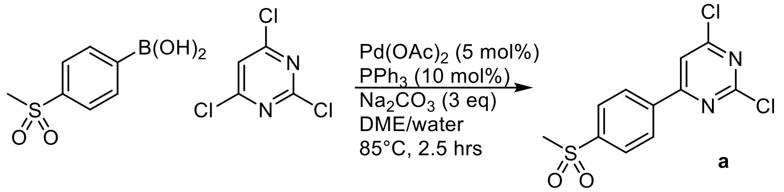

A suspension of (4-(methylsulfonyl)phenyl)boronic acid (600 mg, 2.99 mmol, 1.0 eq.), 2,4,6-trichloropyrimidine (550 mg, 2.99 mmol, 1.0 eq.), sodium carbonate (1.00 g, 9.43 mmol, 3.154 eq.), palladium (II) acetate (35 mg, 0.1558 mmol, 0.052 eq.), and triphenylphosphine (80 mg, 0.305 mmol, 0.102 eq.) in degassed 4:1 DME/water (15 mL) was heated at 85°C for 2.5 hrs. The reaction was cooled to room temperature and poured into water (60 mL), which was extracted with EtOAc (3 x 20 mL), and the combined organic extracts were dried over $MgSO_4$, then evaporated. The residue was purified by flash column chromatography (0-100% EtOAc/hex, eluting at ~ 60%) to provide 2,6-dichloro-4-(4-(methylsulfonyl)phenyl)pyrimidine (**a**) (453.1 mg, 50% yield) as a white solid.

2,6-dichloro-4-(4-(methylsulfonyl)phenyl)pyrimidine (**a**): ^{1}H NMR (300 MHz, $CDCl_3$): δ 8.27 (d, *J* = 8.5 Hz, 2H), 8.10 (d, *J* = 8.6 Hz, 2H), 7.75 (s, 1H), 3.11 (s, 3H). MS-ESI calc'd for $C_{11}H_8Cl_2N_2O_2S$ $[M+H]^+$ 302.96, found 302.98.

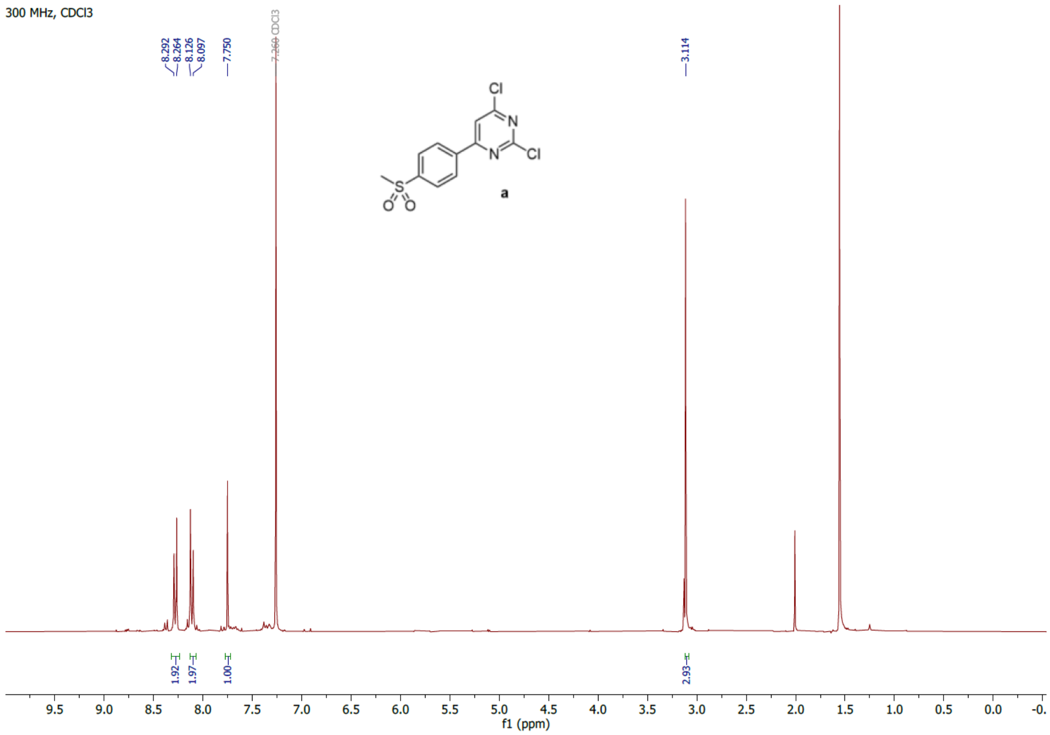

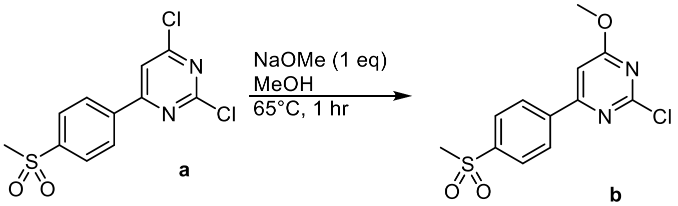

To a suspension of **a** (260 mg, 0.8576 mmol, 1 eq.) in methanol (8 mL) was added sodium methoxide (165 µL, 0.891 mmol, 1.039 eq.). The mixture was heated at 65°C for 1 hr, during which the mixture became a yellow solution. The reaction was cooled to room temperature and evaporated. The solids were suspended in 1:1 DCM/Et_2O and filtered through a plug of celite to remove salts. The ratio of desired product **b** to the 2,6-dimethoxy byproduct was ~ 3:1 and was taken on crude for the next step.

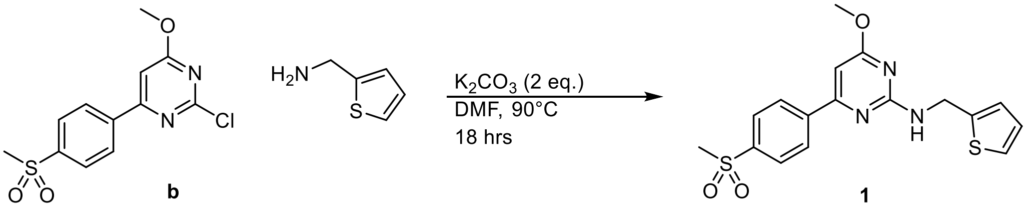

To a stirred solution of the crude mixture of **b** (0.857 mmol) in DMF (4 mL) was added potassium carbonate (240 mg, 1.73 mmol, 2.0 eq.) and 2-thiophenmethylamine (96.9 mg, 0.8569, 1.0 eq.). The reaction was stirred at room temperature for 3 hours, with minimal progress observed. The reaction was then heated to 90°C for 18 hours, then cooled to room temperature, and the DMF was evaporated. The residue was partitioned between water/EtOAc and separated. The aqueous layer was extracted with EtOAc, and the combined organics were dried over $MgSO_4$, then evaporated. The residue was purified by flash column chromatography (0-10% MeOH/CH_2Cl_2) to provide **1** (85.0 mg, 25.9% yield) as a white solid.

Synthesis of **2**

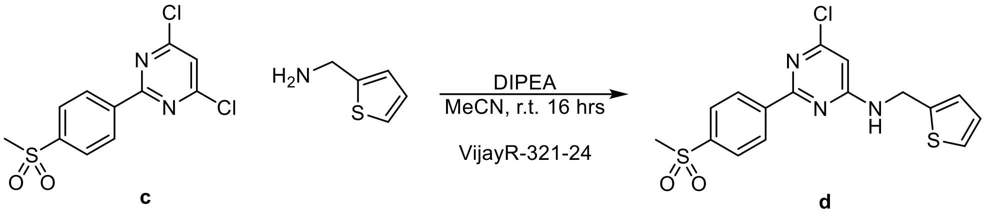

To a solution of 4,6-dichloro-2-(4-(methylsulfonyl)phenyl)pyrimidine (**c**)[2] (0.1 g, 0.3298 mmol, 1 eq.) in MeCN (3.5 mL) was added DIPEA (171 µL, 0.9894 mmol, 3.0 eq.) and thiophen-2-ylmethanamine (37.3 mg, 0.3298 mmol, 1 eq.) and the reaction was stirred at room temperature for 16 hrs. After which, the reaction was concentrated, then suspended in water (30 mL), followed by extraction with EtOAc (3 x 20 mL). The combined organics were dried over Na_2SO_4 and evaporated. The residue was purified via Biotage (5:1 hex/EtOAc); 12S column) to provide impure product, which was further purified by prep HPLC (30-50% MeCN in water, 0.1% HCO_2H) to provide the title compound 6-chloro-2-(4-(methylsulfonyl)phenyl)-N-(thiophen-2-ylmethyl)amine (**d**) (0.051 g, 40.7% yield) as an of white solid. 6-chloro-2-(4-(methylsulfonyl)phenyl)-N-(thiophen-2-ylmethyl)amine (**d**): ^{1}H NMR (400 MHz, DMSO-d_6): δ 8.55 (d, *J* = 8.2 Hz, 2H), 8.50 (m, 1H), 8.05 (d, *J* = 8.5 Hz, 2H), 7.39 (d, *J* = 5.1 Hz, 1H), 7.12 (d, *J* = 3.5 Hz, 1H), 6.97 (dd, *J* = 5.1, 3.4 Hz, 1H0, 6.58 (br s, 1H), 4.87 (d, *J* = 5.7 Hz, 2H), 3.27 (s, 3H). MS-ESI calc'd for $C_{16}H_{14}ClN_3O_2S_2$ $[M+H]^+$ 380.02, found 380.20.

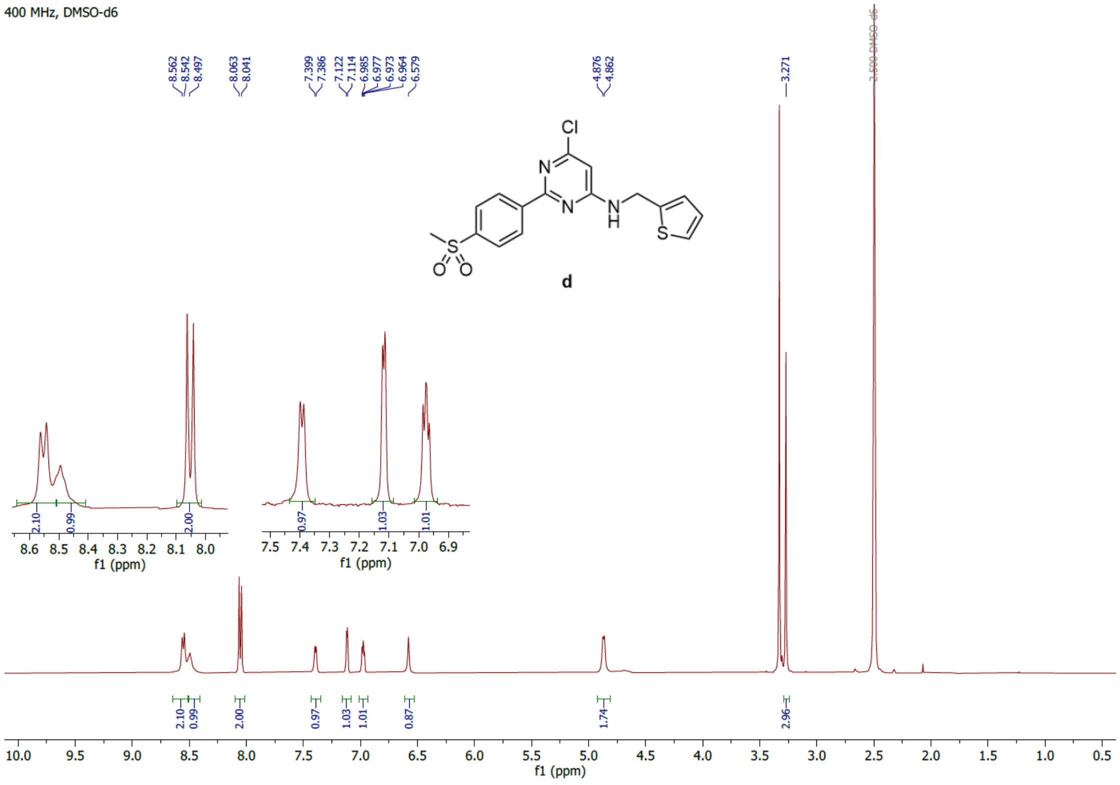

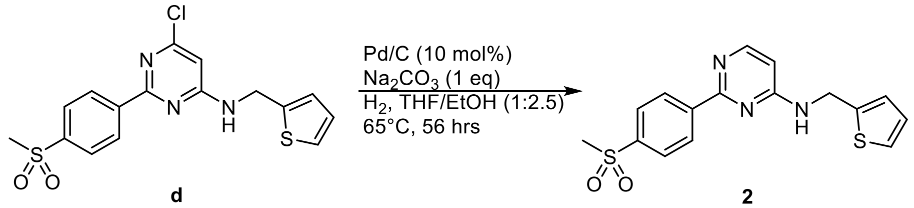

A mixture of **d** (100 mg, 0.2632 mmol, 1.0 eq.), 10% palladium on carbon (28 mg, 0.02631, 0.1 eq.), and sodium carbonate (30 mg, 0.2830 mmol, 1.075 eq.) in ethanol (2.5 mL) was stirred at room temperature under a hydrogen atmosphere for 90 minutes, during which minimal conversion occurred. THF (1 mL) was added, as solubility in ethanol appears to be limited, and stirring was continued for 2 hrs, with no further progress. The reaction was then heated to 65°C for 56 hrs, periodically replenishing the hydrogen atmosphere. After 48 hours, 9:1 product to starting material was noticed, and after 56 hours the reaction was cooled to room temperature, filtered through celite, and washed with 1:1 CH_2Cl_2/EtOH. The solvent was evaporated to afford **2** (48 mg, 49.6 % yield).

<u>Synthesis of **4**</u>

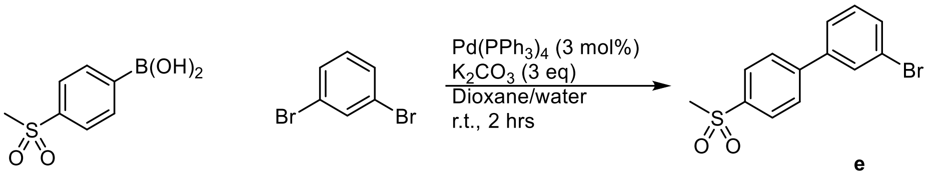

In a flask under nitrogen with 1,3-dibromobenzene (519 µL, 4.32 mmol, 2 eq.), boronic acid (422 mg, 2.11 mmol, 1 eq.), and potassium carbonate (876 mg, 6.34 mmol, 3 eq.), palladium tetrakis was added (73.3 mg, 0.0634 mmol, 3 mol%). The mixture was degassed for ten minutes, then diluted with water (8 mL) and 1,4-dioxane (32 mL), and then stirred at room temperature for 2 hours. After completion, the solvent was evaporated and the aqueous layer was extracted with EtOAc (3 x 10 mL), and the combined organics were dried over $MgSO_4$, then concentrated. The title compound was then purified using flash column chromatography (0-100% EtOAc/hex) to afford **e** (410 mg, 62.5% yield).
3-bromo-4'-(methylsulfonyl)-1,1'-biphenyl (**e**): ^{1}H NMR (400 MHz, $CDCl_3$) δ 8.02 (dt, *J* = 12.8, 1.7 Hz, 2H), 7.77 – 7.72 (m, 3H), 7.55 (dddd, *J* = 14.0, 7.8, 1.9, 1.1 Hz, 2H), 7.36 (t, *J* = 7.9 Hz, 1H), 3.10 (s, 3H).
^{1}H NMR

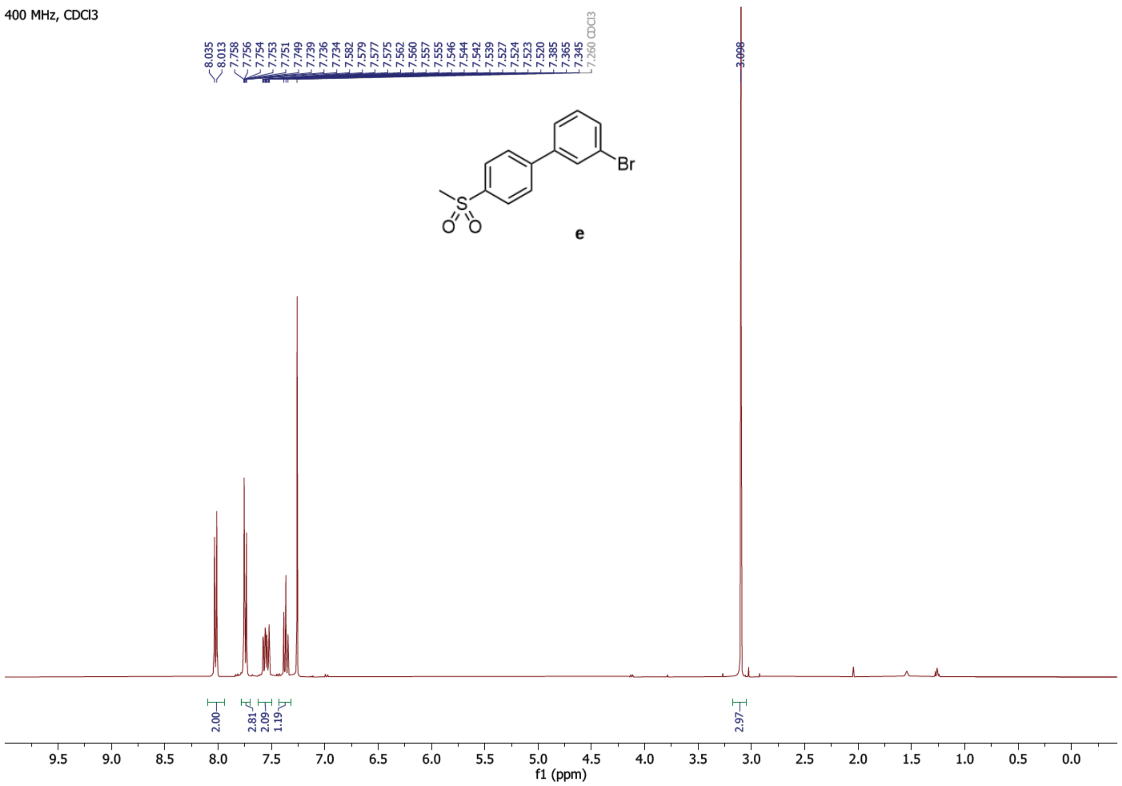

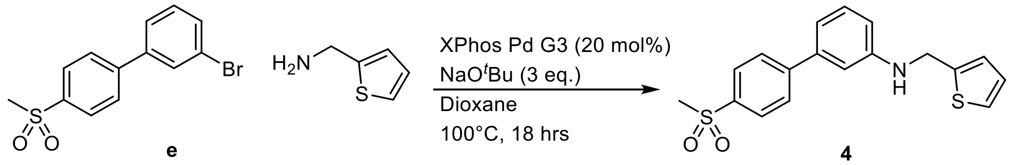

In a flask under nitrogen flow with **e** (100 mg, 0.3213 mmol, 1 eq.), 2-thiophen-yl-methylamine (130 µL, 1.28 mmol, 4 eq.), NaO^{t}Bu (92.6 mg, 0.9639 mmol, 3 eq.) was added Xphos Pd G3 (54.3 mg, 0.06426 mmol, 20 mol%). The flask was degassed for 10 minutes, then suspended in 1,4-dioxane (1 mL). the dark mixture was stirred overnight at 100°C. After completion, the solvent was evaporated, then suspended in water (5 mL) and extracted with EtOAc (3 x 10 mL). The combined extracts were dried over $MgSO_4$, and concentrated. The crude mixture was purified using flash column chromatography (0-100% EtOAc/hex), and then again with 0-100% MeOH/DCM after impurities remained. A pure fraction was collected, affording **4** (5.1 mg, 4.6%).

Synthesis of **6**

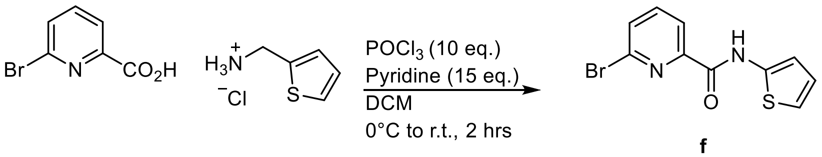

To a stirred suspension of 6-bromopicolinic acid (0.3 g, 1.48 mmol, 1 eq.), thiophen-2-ylmethylamine hydrochloride (200 mg, 1.48 mmol, 1 eq.), and pyridine (1.78 mL, 22.2 mmol, 15 eq.) in dichloromethane (5 mL) was added dropwise phosphorus oxychloride (1.37 mL, 14.8 mmol, 10 eq.) at 0°C. After addition,

the reaction was warmed to room temperature and stirred for 2 hours, after which the reaction was quenched with saturated sodium bicarbonate (150 mL) and extracted with dichloromethane (3 x 25 mL). The combined organics were washed with 10% citric acid (2 x 10 mL), dried over Na_2SO_4, and concentrated. The residue was purified via biotage (2:1 Hex/EtOAc; 12S column) to provide 6-bromo-*N*-thiophen-2-yl)pyridine-2-carboxamide (f, 0.09 g, 18.4% yield) as a light-yellow solid. After confirming the molecular weight of the product via LCMS, the compound was used in the next step without further characterization.

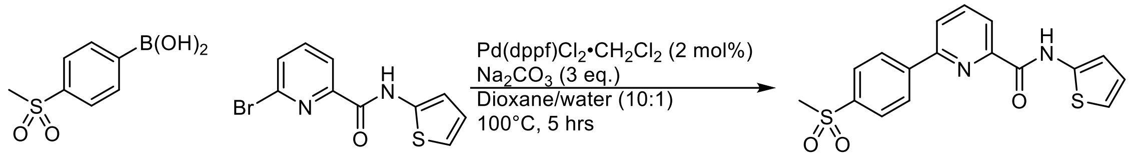

f **6**

A stirred suspension of **f** (0.09 g, 0.3178 mmol, 1 eq.), 4-(methylsulfonyl)phenylboronic acid (76.2 mg, 0.3813 mmol, 1.2 eq.), and sodium carbonate (101 mg, 0.9534 mmol, 3.0 eq.) in dioxane (3 mL) was degassed with nitrogen for 15 min. After which, $Pd(dppf)Cl_2{\cdot}CH_2Cl_2$ (5.19 mg, 0.006356 mmol, 2 mol%) was added, followed by water (0.3 mL) and the reaction was heated to 100°C for 5 hr. After completion, the reaction was cooled to room temperature and diluted with water (60 mL) and extracted with EtOAc (2 x 20 mL). The combined organic extracts were dried over Na_2SO_4 and evaporated. The residue was purified via Biotage (2:1 Hex/EtOAc; 12S column) to provide **6** (0.07 g, 61.5% yield) as an off-white solid.

<u>Synthesis of **7**</u>

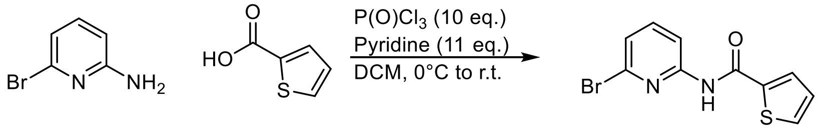

g

N-(6-bromo-pyridin-2-yl)-thiophen-2-carboxamide **g** was synthesized according to the same procedure as **f** from 0.1 g of 6-bromo-2-aminopyridine, 0.13 g of product was formed as a light-yellow solid, 75.4% yield. After confirming the expected mass via LCMS, the compound was used in the final step without further characterization.

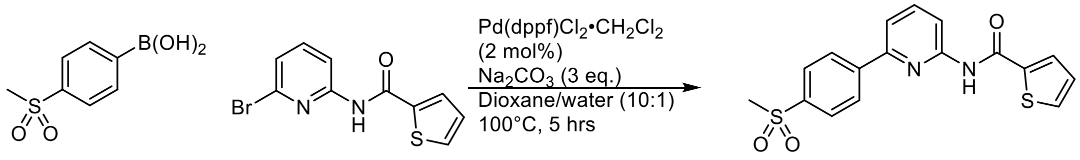

g **7**

Synthesis of **7**: Following the same Suzuki conditions for **6**, compound **7** was synthesized from *N*-(6-bromo-pyridin-2-yl)thiophen-2-carboxamide **g** (0.13 g, 0.4592 mmol, 1 eq.) and 4-(methylsulfonyl)phenylboronic acid to obtain 0.12 g (73.1% yield) as an off-white solid.

Analytical Data

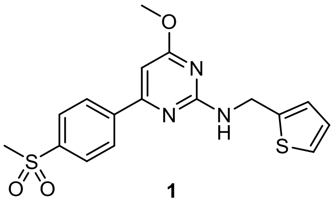

1

4-methoxy-6-(4-(methylsulfonyl)phenyl)-*N*-(thiophen-2-ylmethyl)-pyrimidin-2-amine (**1**): ^{1}H NMR (400 MHz, $CDCl_3$): δ 8.18 (d, *J* = 8.1 Hz, 2H), 8.01 (d, *J* = 8.1 Hz, 2H), 7.20 (d, *J* = 5.0 Hz, 1H), 7.03 (d, *J* = 3.4 Hz, 1H), 6.96 (dd, *J* = 5.1, 3.4 Hz, 1H), 6.51 (s, 1H), 5.56 (br s, 1H), 4.87 (d, *J* = 5.5 Hz, 2H), 3.95 (s, 3H), 3.06 (s, 3H). MS-ESI calc'd for $C_{17}H_{17}N_3O_3S_2$ $[M+H]^+$ 376.08, found 376.05.

^{1}H NMR:

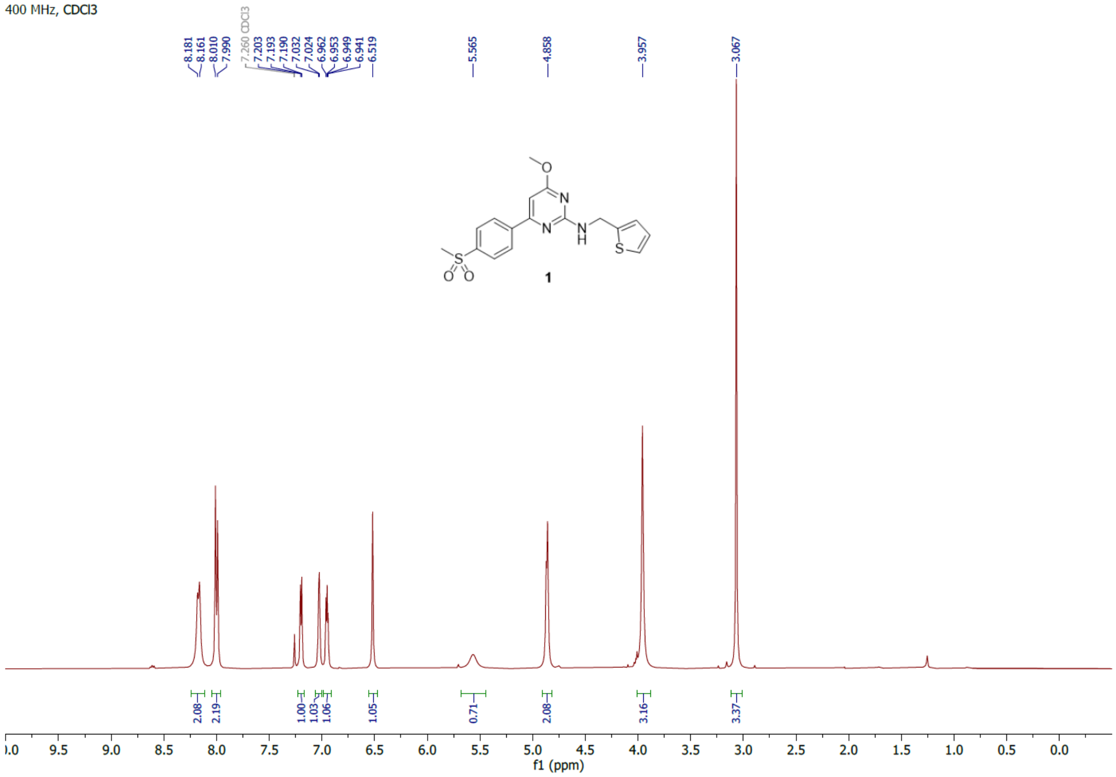

UPLC

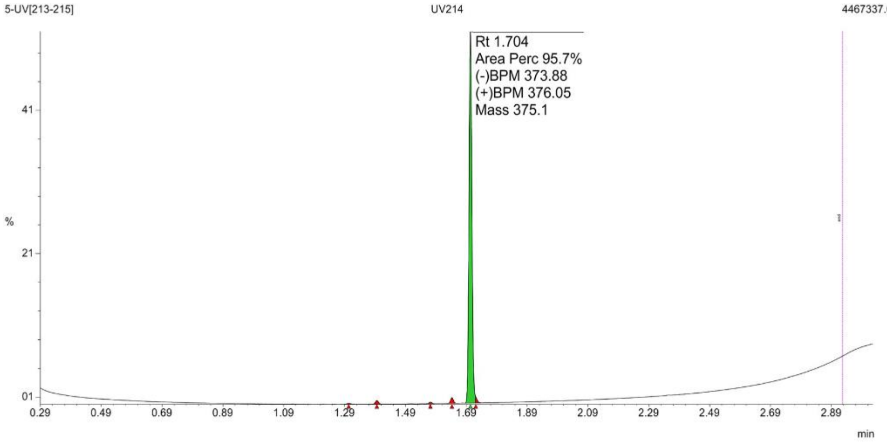

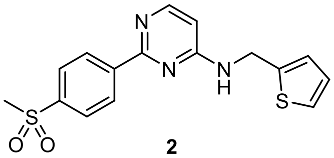

2

2-(4-(methylsulfonyl)phenyl)-*N*-(thiophen-2-ylmethyl)pyrimidin-4-amine (**2**): ^{1}HNMR (300 MHz, $CDCl_3$): δ 8.61 (d, *J* = 8.6 Hz, 2H), 8.31 (d, *J* = 5.9 Hz, 1H), 8.01 (d, *J* = 8.6 Hz, 2H), 7.23 (dd, *J* = 5.0, 1.3 Hz, 1H), 7.05 (d, *J* = 3.3 Hz, 1H), 6.97 (dd, *J* = 5.1, 3.4 Hz, 1H), 6.35 (d, *J* = 5.9 Hz, 1H), 5.46 (br s, 1H), 4.86 (d, *J* = 5.8 Hz, 2H), 3.08 (s, 3H). MS-ESI calc'd for $C_{16}H_{15}N_3O_2S_2$ $[M+H]^+$ 346.06, found 346.14.

^{1}H NMR:

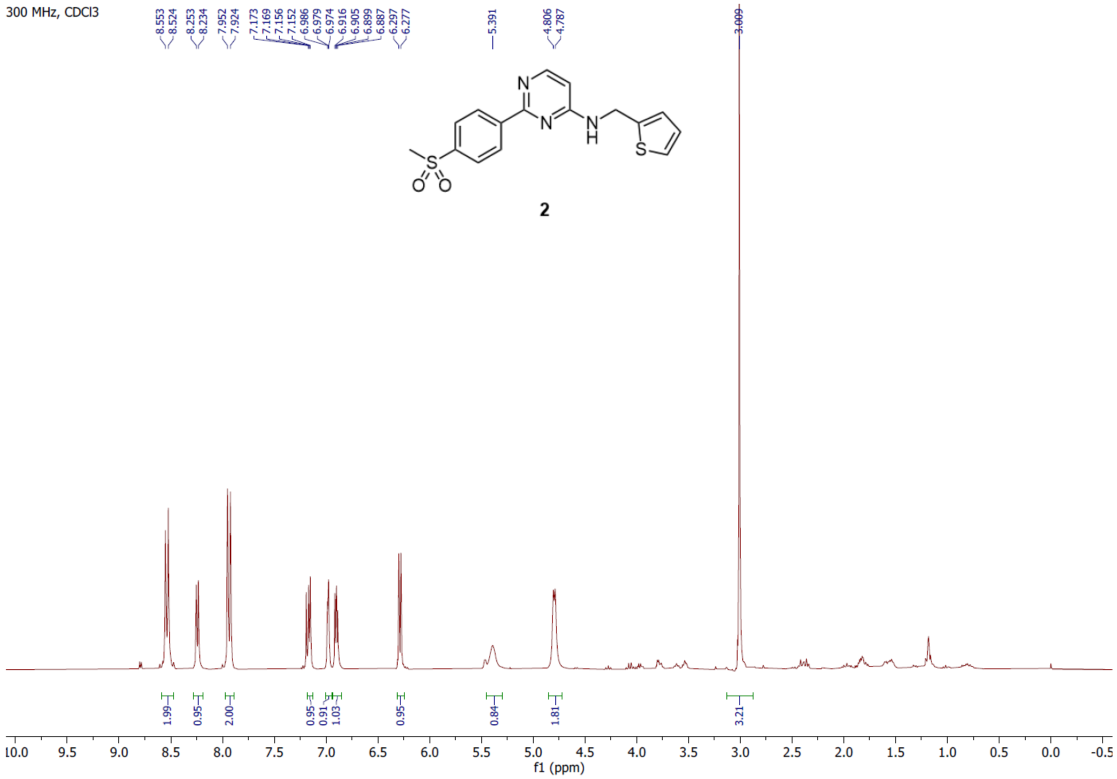

UPLC:

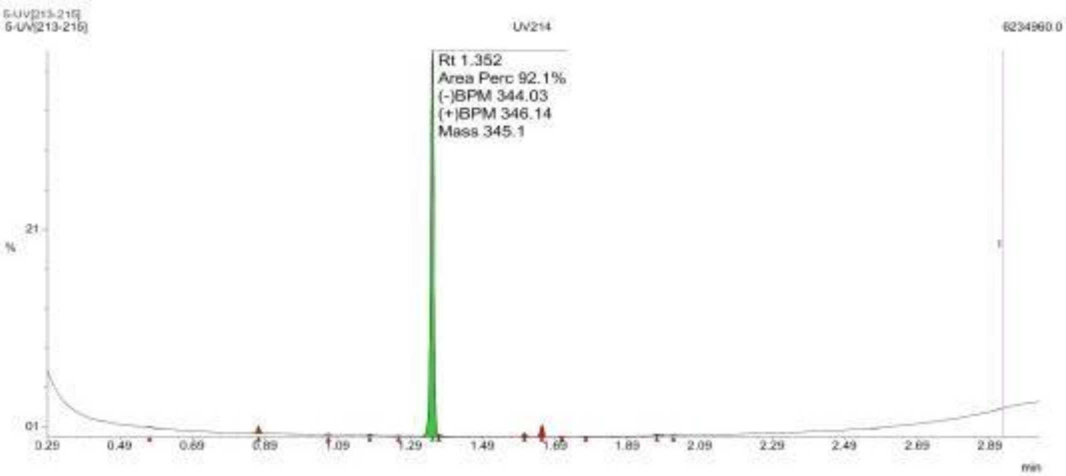

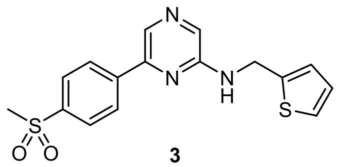

3

6-(4-methylsulfonyl)phenyl)-*N*-(thiophen-2-ylmethyl)pyrazin-2-amine (**3**): ^{1}H NMR (300 MHz, $CDCl_3$): δ 8.39 (s, 1H), 8.22 (dt, *J* = 8.7, 1.8 Hz, 2H), 8.04 (dt, *J* = 8.8, 1.9 Hz, 2H), 7.95 (s, 1H), 7.24 (dd, *J* = 5.1, 1.3 Hz, 1H), 7.07 (dq, *J* = 3.5, 1.0 Hz, 1H), 6.98 (dd, *J* = 5.1, 3.5 Hz, 1H), 5.1 (t, *J* = 5.6 Hz, 1H), 4.88 (d, *J* = 5.9 Hz, 2H), 3.10 (s, 3H).). MS-ESI calc'd for $C_{16}H_{15}N_3O_2S_2$ $[M+H]^+$ 346.06, found 345.99.

^{1}H NMR:

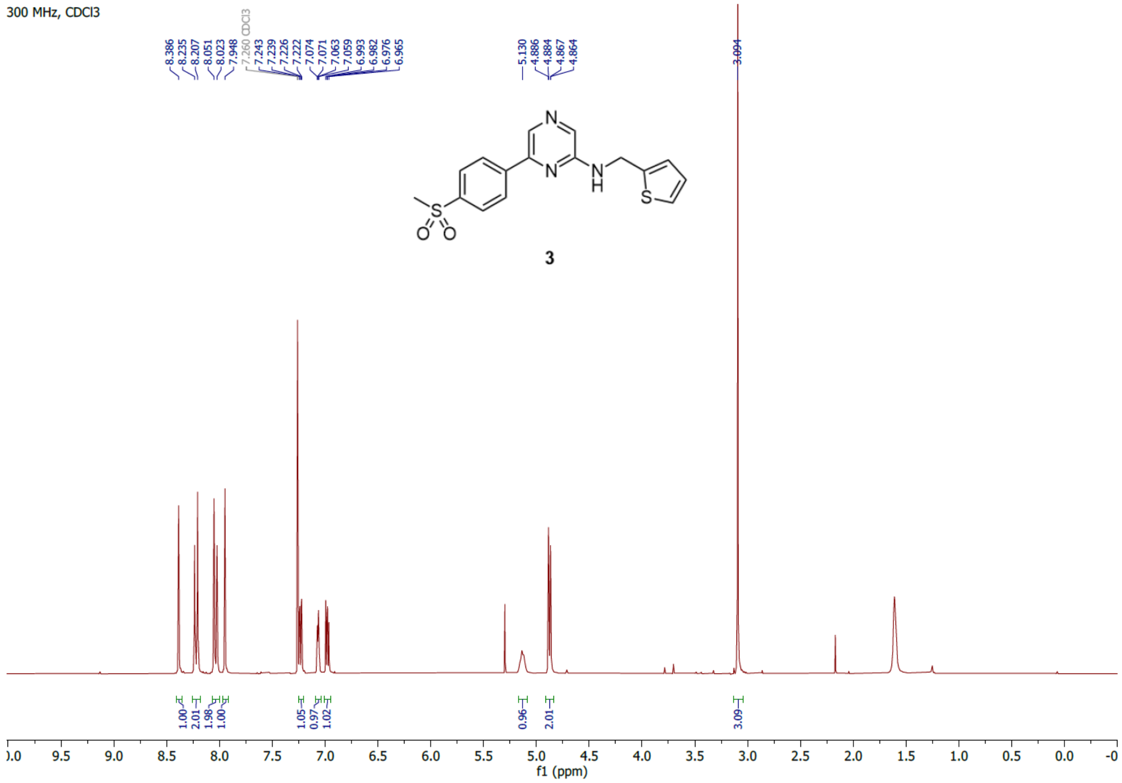

UPLC

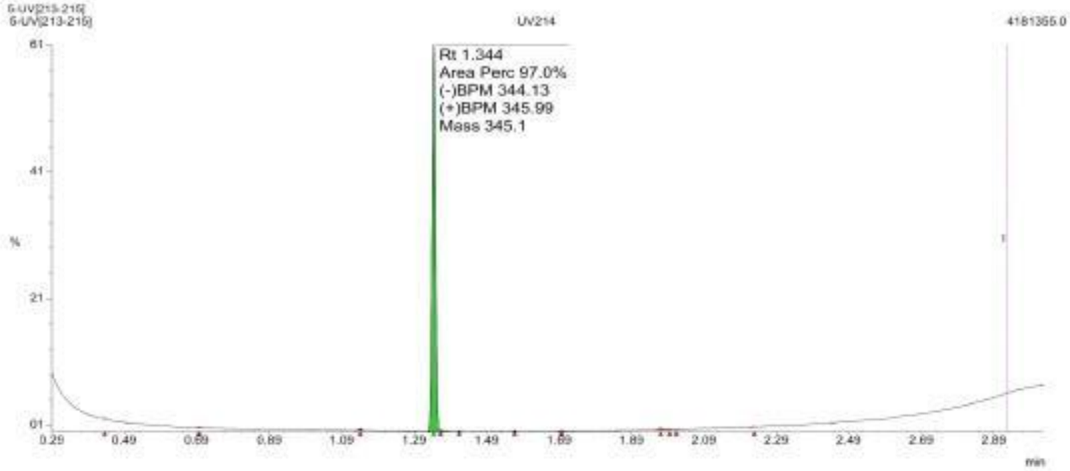

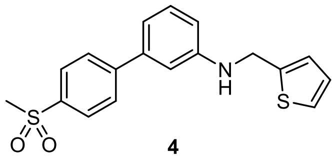

4

4'-(methylsufonyl)-*N*-(thiophen-2-ylmethyl)-[1,1'-biphenyl]-3-amine (**4**): ^{1}H NMR (400 MHz, $CDCl_3$): δ 7.90 (d, *J* = 8.4 Hz, 2H), 7.65 (d, *J* = 8.4 Hz, 2H), 7.23 (t, *J* = 7.9 Hz, 1H), 7.17 (dd, *J* = 5.2, 1.2 Hz, 1H), 6.98 (dd, *J* = 3.4, 1.1 Hz, 1H), 6.93-6.89 (m, 2H), 6.81 (t, *J* = 2.0 Hz, 1H), 6.68 (m, 1H), 4.52 (d, *J* = 1.0, 2H), 4.34 (br s, 1H), 3.01 (s, 3H). MS-ESI calc'd for $C_{18}H_{17}NO_2S_2$ $[M+H]^+$ 344.07, found 344.25.

^{1}H NMR

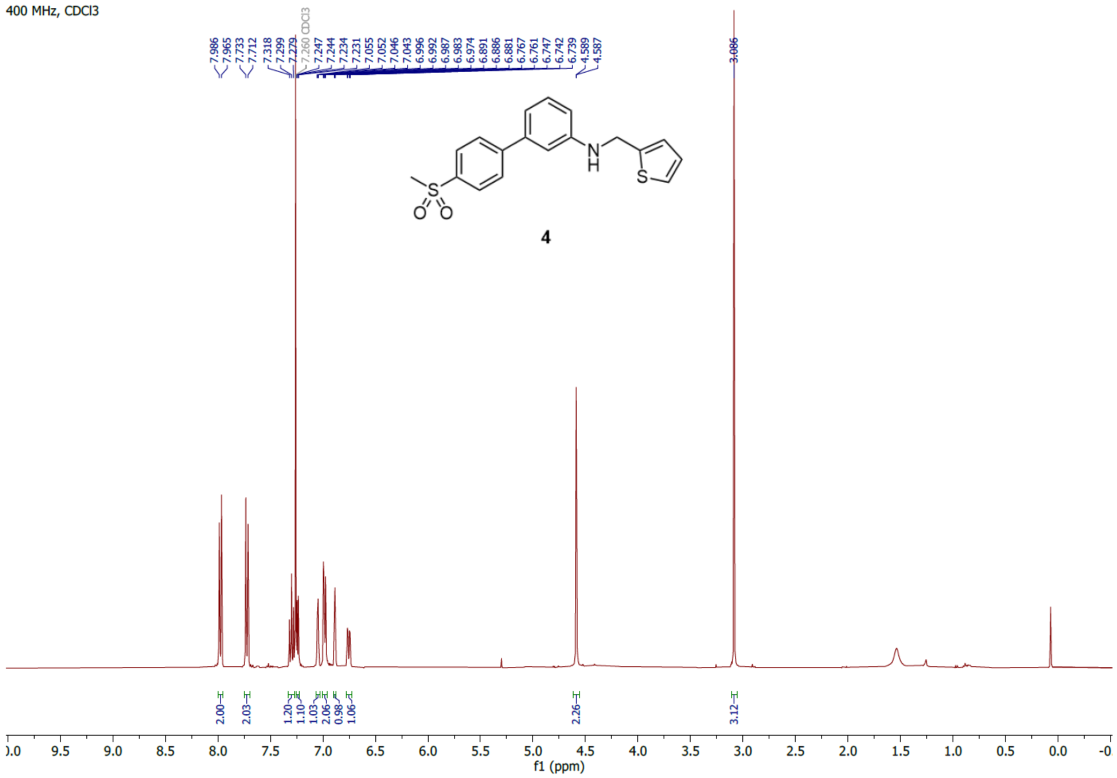

UPLC

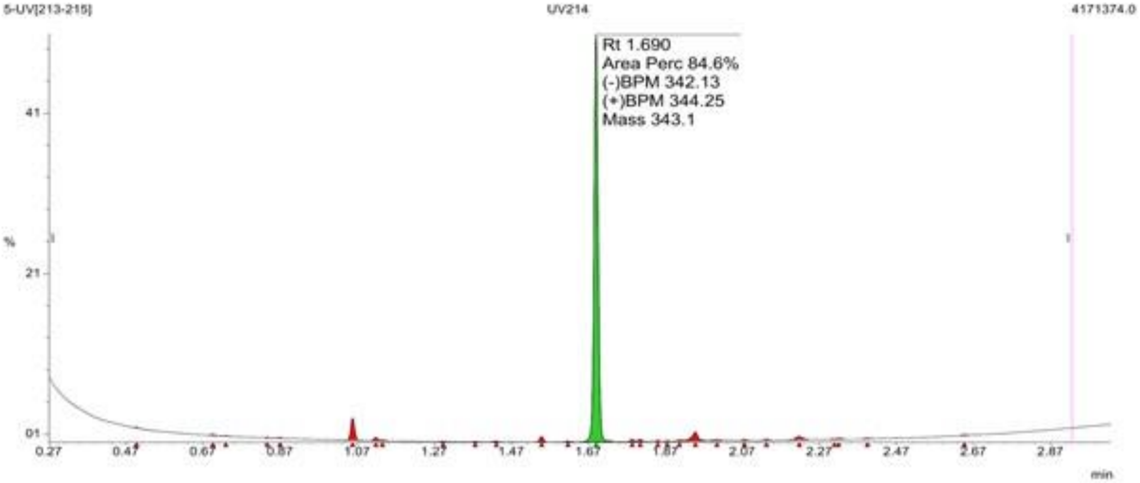

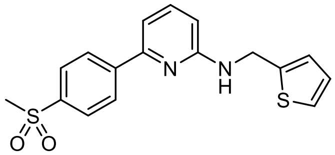

5

6-(4-methylsulfonyl)phenyl)-*N*-(thiophen-2-yl)pyridin-2-amine (**5**): ^{1}H NMR (300 MHz, CDCl$_3$): δ 8.11 (d, *J* = 8.5 Hz, 2H), 7.94 (d, *J* = 8.5 Hz, 2H), 7.88 (dd, *J* = 9.1, 7.4 Hz, 1H), 7.28 (dd, *J* = 5.1, 1.3 Hz, 1H), 7.09 (dd, *J* = 3.5, 1.0 Hz, 1H), 7.00 (m, 2H), 6.90 (dd, *J* = 9.1, 0.9 Hz, 1H), 4.79 (d, *J* = 1.0 Hz, 2H), 3.12 (s, 3H). MS-ESI calc'd for $C_{17}H_{16}N_2O_2S_2$ $[M+H]^+$ 345.07, found 344.89.

^{1}H NMR

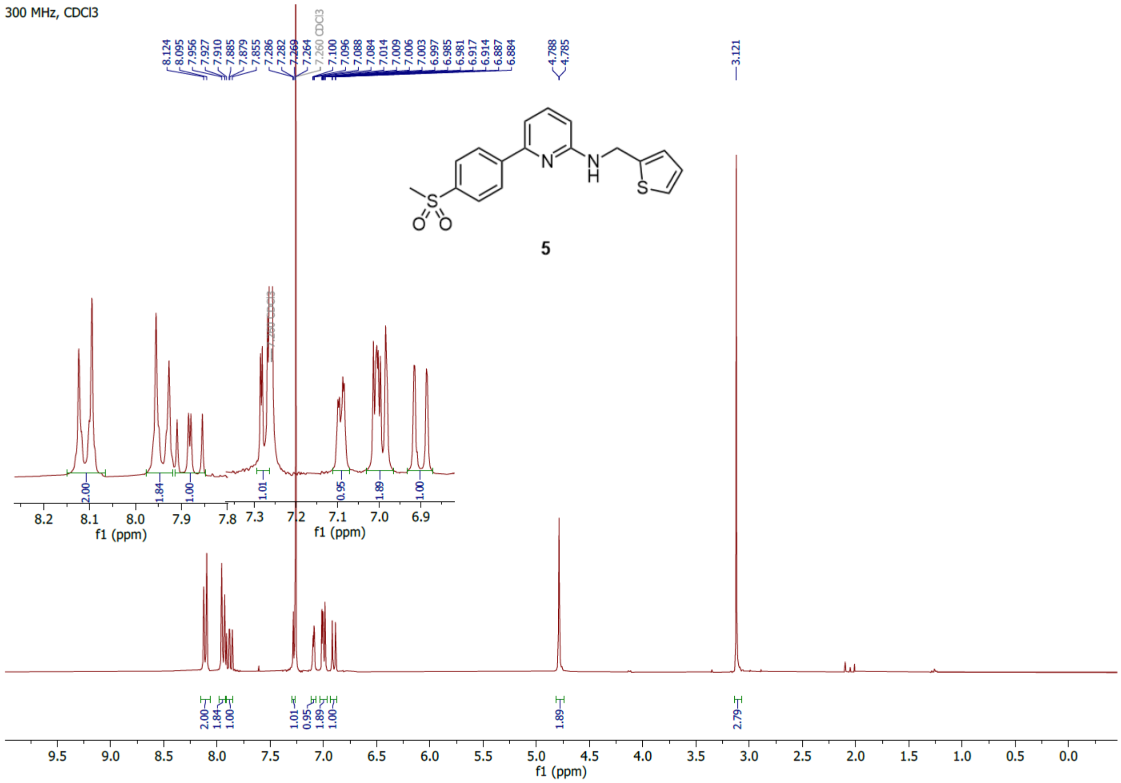

UPLC

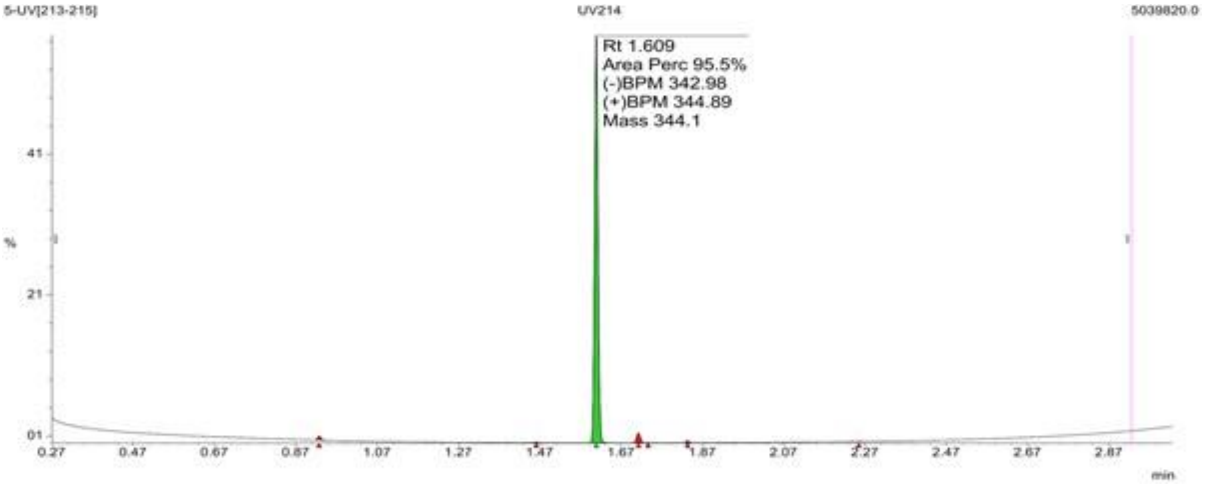

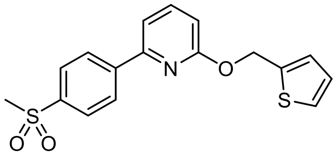

BRD2369

2-(4-(methylsulfonyl)phenyl)-6-(thiophen-2-ylmethoxy)pyridine (**BRD2369**): ^{1}H NMR (400 MHz, DMSO-d_6): δ 8.44 (d, *J* = 8.3 Hz, 2H), 8.06 (d, *J* = 8.3 Hz, 2H), 7.90 (t, *J* = 7.8 Hz, 1H), 7.77 (d, *J* = 7.4 Hz, 1H), 7.53 (dd, *J* = 5.0, 1.3 Hz, 1H), 7.29 (d, *J* = 3.4 Hz, 1H), 7.04 (dd, *J* = 5.1, 3.5 Hz, 2H), 6.92 (d, *J* = 8.2 Hz, 1H), 5.71 (s, 2H), 3.30 (s, 3H). MS-ESI calc'd for $C_{17}H_{15}NO_3S_2$ $[M+H]^+$ 346.05, found 346.45.

^{1}H NMR

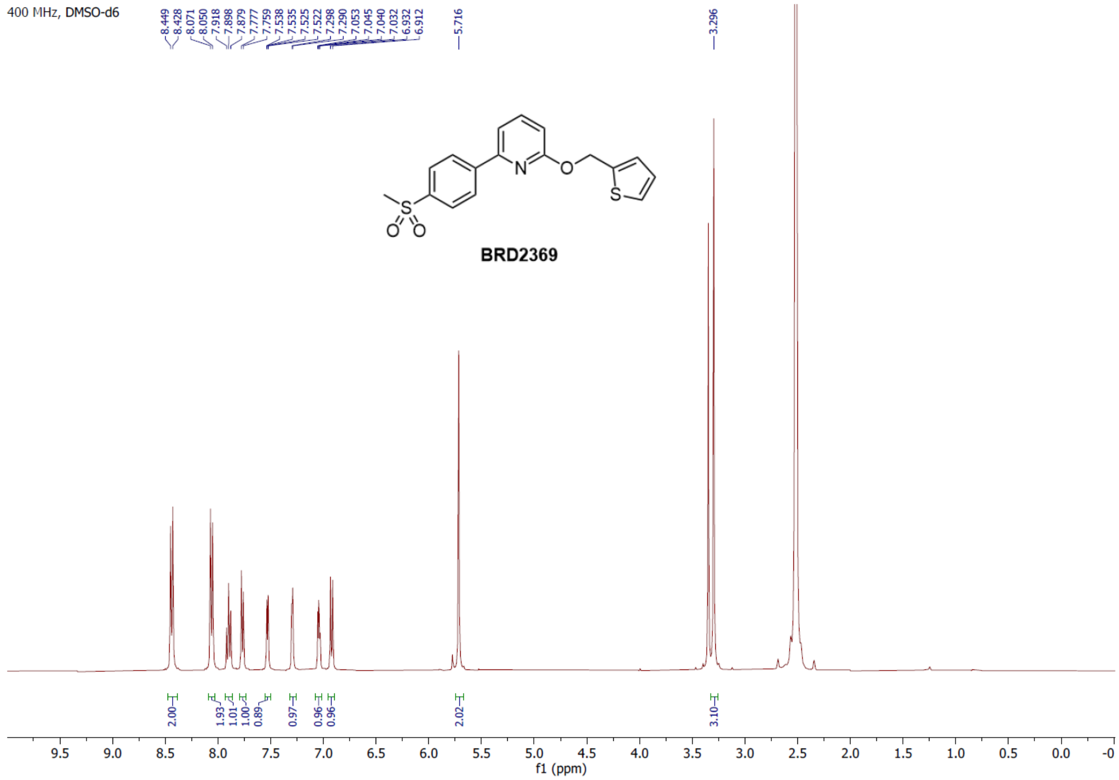

UPLC

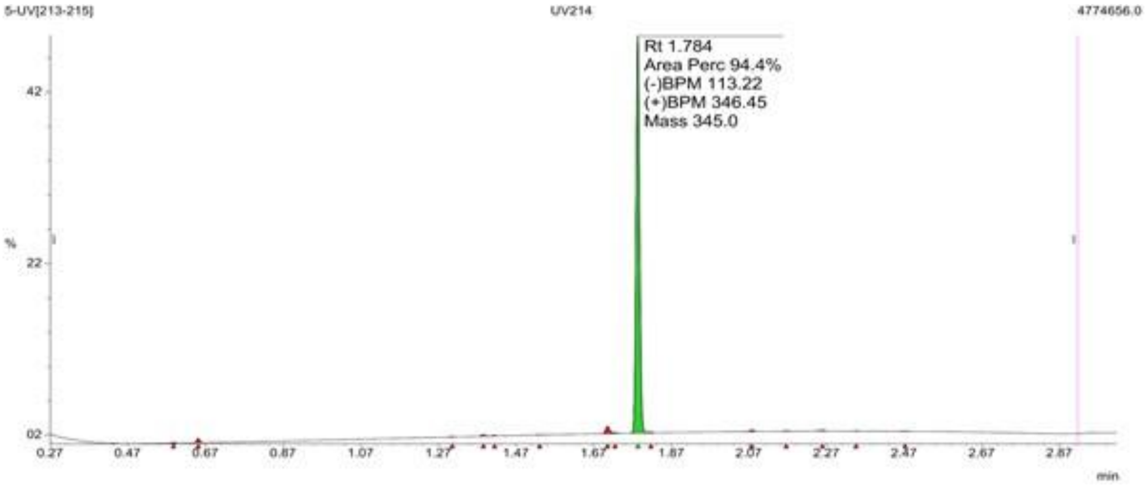

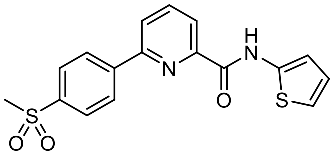

6

6-(4-(methylsulfonyl)phenyl)-*N*-(thiophen-2-yl)picolinamide (**6**): ^{1}H NMR (400 MHz, DMSO-d$_6$): δ 11.71 (s, 1H), 8.65 (d, *J* = 8.4 Hz, 2H), 8.37 (d, *J* = 7.6 Hz, 1H), 8.24-8.16 (m, 2H), 8.10 (d, *J* = 8.2 Hz, 2H), 7.20 (dd, *J* = 3.8, 2.5 Hz, 1H), 7.10 (d, *J* = 5.4 Hz, 1H), 6.97 (dd, *J* = 5.5, 3.9 Hz, 1H), 3.31 (s, 3H). MS-ESI calc'd for $C_{17}H_{14}N_2O_3S_2$ $[M+H]^+$ 359.05, found 359.06.

^{1}H NMR

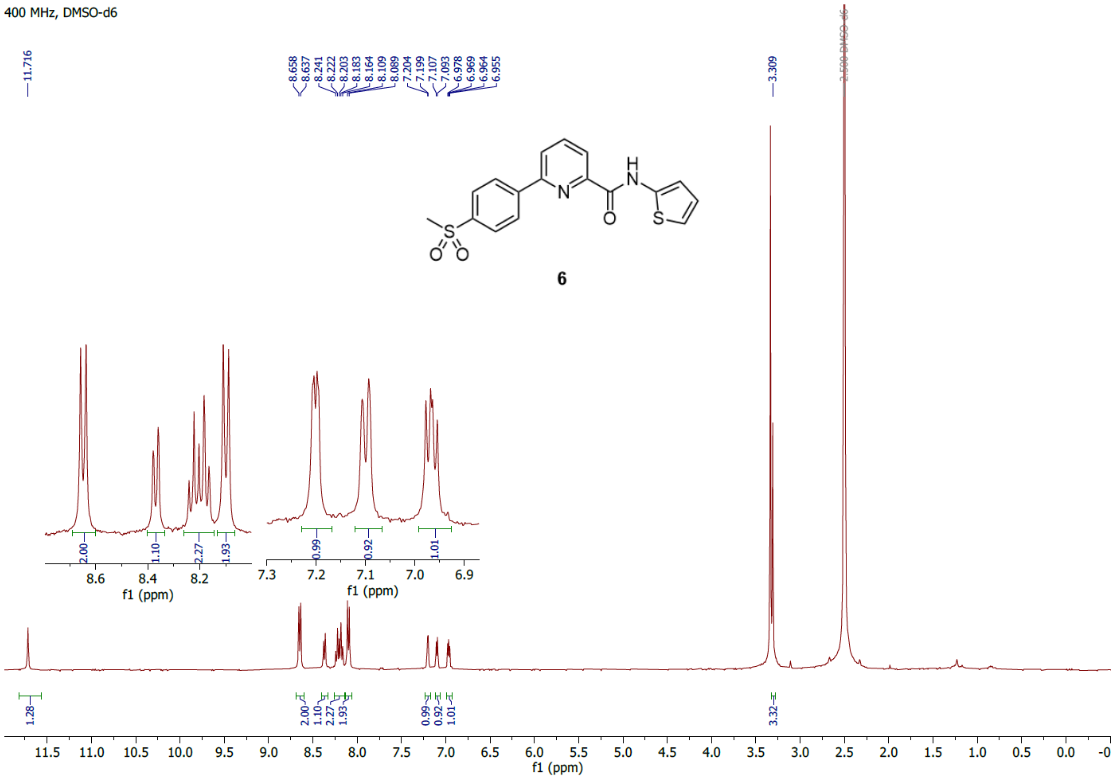

UPLC

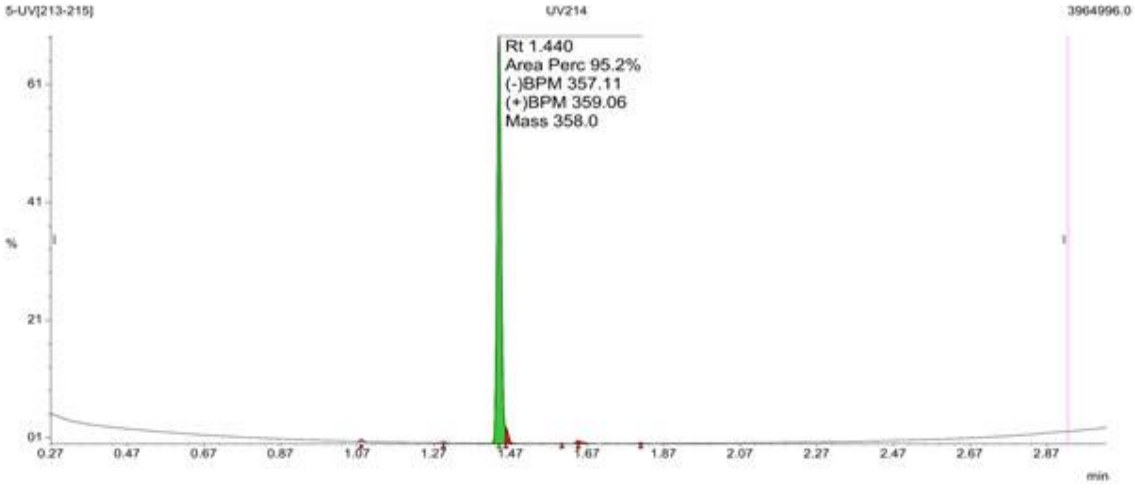

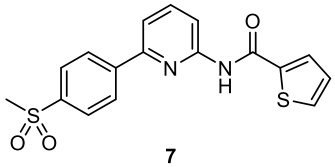

7

N-(6-(4-methylsulfonyl)phenyl)pyridine-2-yl)thiophen-2-carboxamide (**7**): ^{1}H NMR (400 MHz, DMSO-d_6): δ 10.91 (s, 1H), 8.40 (d, *J* = 8.2 Hz, 2H), 8.30 (d, *J* = 3.8 Hz, 1H), 8.15 (d, *J* = 8.2 Hz, 1H), 8.06 (d, *J* = 8.1 Hz, 2H), 8.00 (t, *J* = 7.9 Hz, 1H), 7.92 (d, *J* = 4.9 Hz, 1H), 7.87 (d, *J* = 7.6 Hz, 1H), 7.25 (t, *J* = 3.6 Hz, 1H), 3.28 (s, 3H). MS-ESI calc'd for $C_{17}H_{14}N_2O_3S_2$ $[M+H]^+$ 359.05, found 359.11.

^{1}H NMR

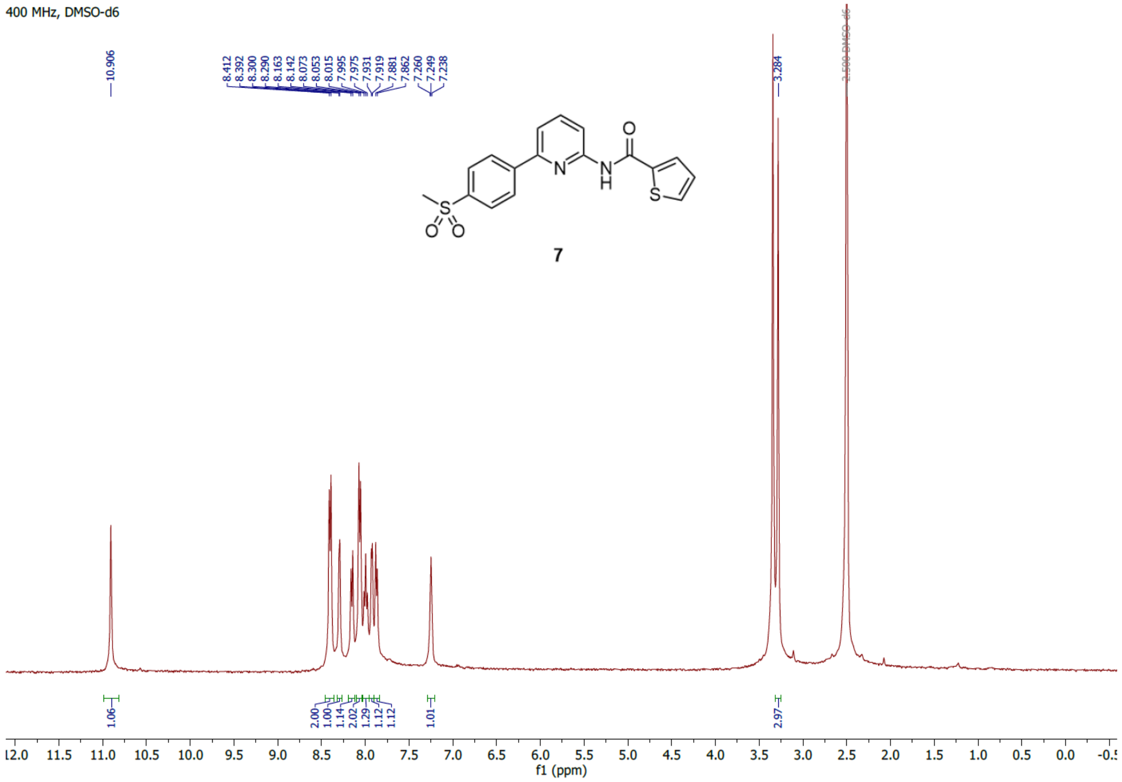

UPLC

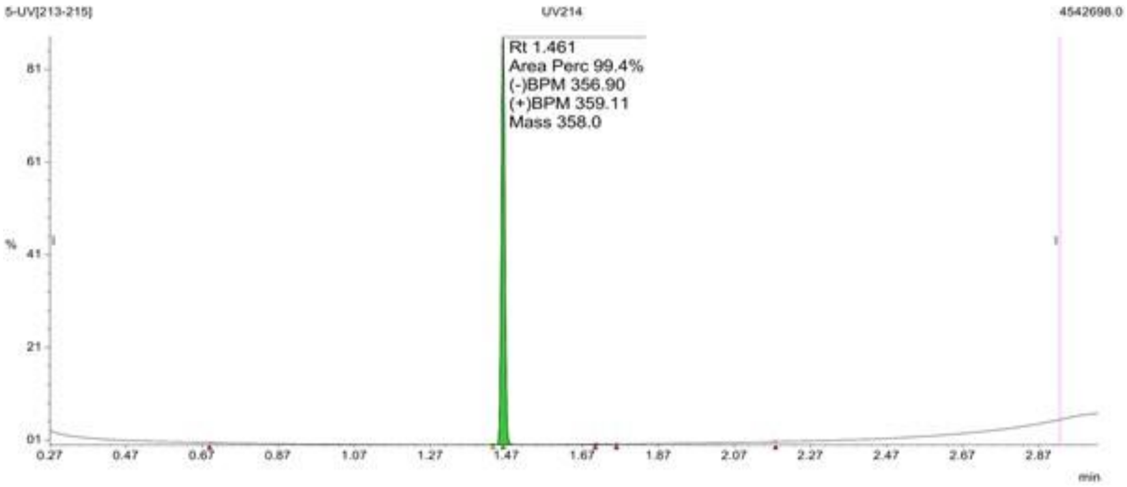

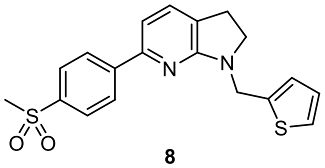

8

6-(4-(methylsulfonyl)phenyl)-1-(thiophen-2-ylmethyl-(2,3-dihydro-1*H*-pyrrolo[2,3-*b*]pyridine (**8**): ^{1}H NMR (400 MHz, DMSO-d$_6$): δ 8.32 (d, *J* = 8.5 Hz, 2H), 7.98 (d, *J* = 8.5 Hz, 2H), 7.43-7.37 (m, 2H), 7.23 (d, *J* = 7.4 Hz, 1H), 7.09 (d, *J* = 3.5 Hz, 1H), 6.98 (dd, *J* = 5.1, 3.4 Hz, 1H), 4.81 (s, 2H), 3.47 (t, *J* = 8.3 Hz, 2H), 3.24 (s, 3H), 2.98 (t, *J* = 8.3 Hz, 2H). MS-ESI calc'd for $C_{19}H_{18}N_2O_2S_2$ [M+H]$^+$ 371.08, found 371.50.

^{1}H NMR

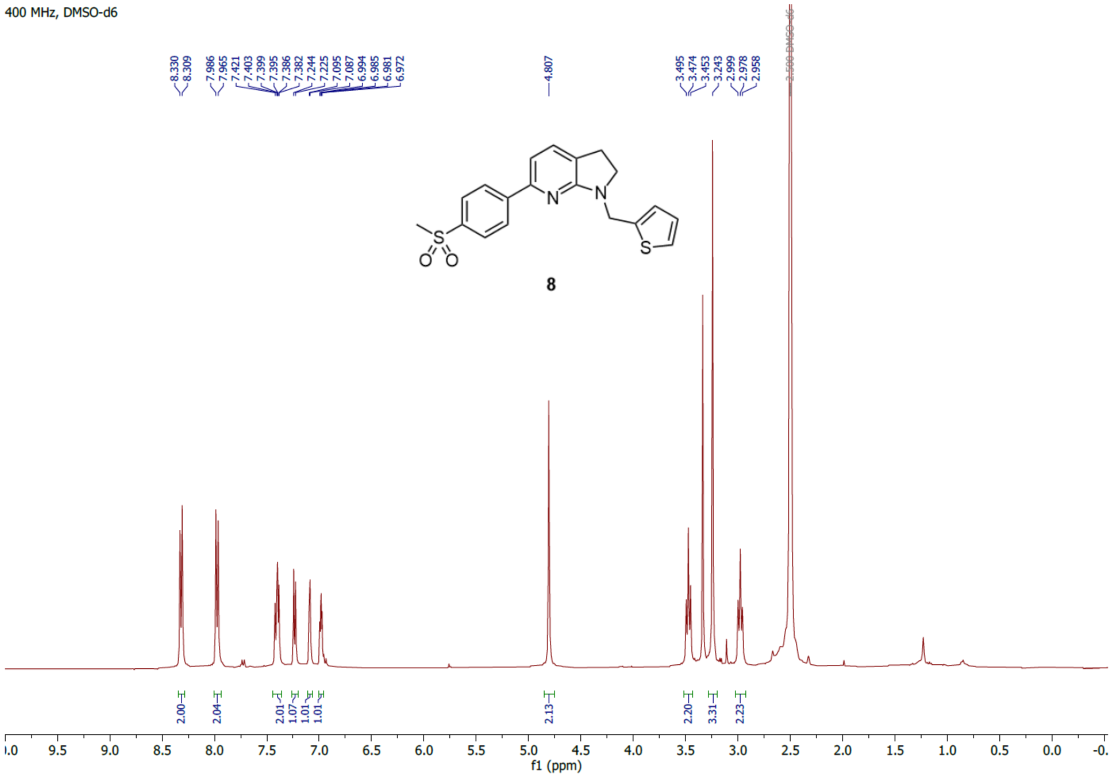

UPLC

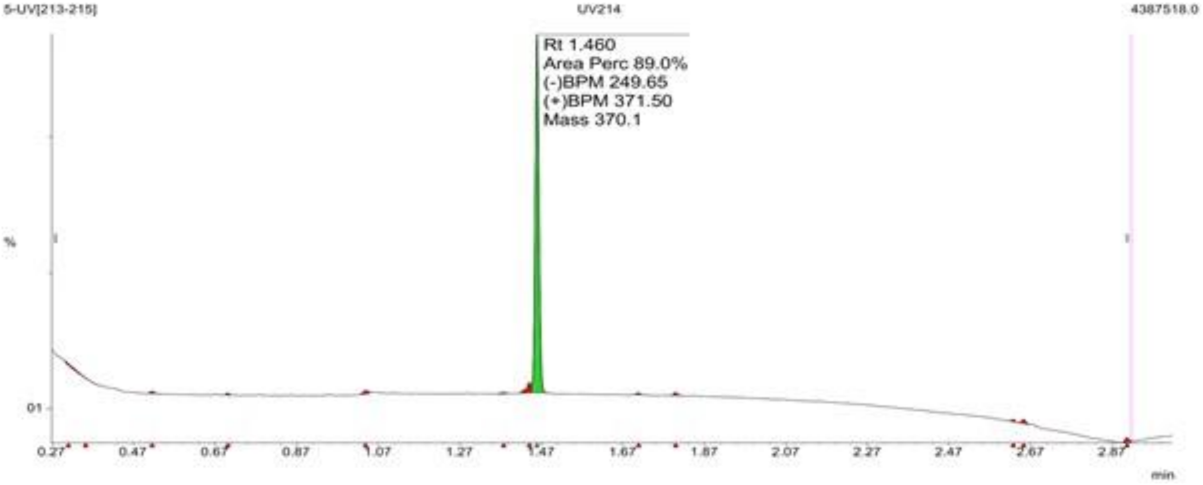

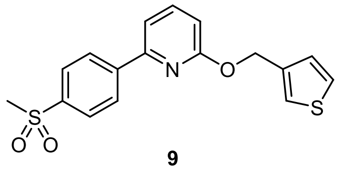

9

2-(4-(methylsulfonyl)phenyl)-6-(thiophen-3-ylmethoxy)pyridine (**9**): ^{1}H NMR (400 MHz, $CDCl_3$): δ 8.25 (d, *J* = 8.6 Hz, 2H), 8.06 (d, *J* = 8.6 Hz, 2H), 7.73 (t, *J* = 7.8 Hz, 1H), 7.45 (d, *J* = 7.4 Hz, 1H), 7.41 (d, *J* = 2.1 Hz, 1H), 7.36 (dd, *J* = 5.0, 3.0 Hz, 1H), 7.23 (dd, *J* = 4.9, 1.3 Hz, 1H), 6.86 (dd, *J* = 8.4 Hz, 1H), 5.55 (s, 2H), 3.12 (s, 3H). MS-ESI calc'd for $C_{17}H_{15}NO_3S_2$ $[M+H]^+$ 346.05, found 346.00.

^{1}H NMR

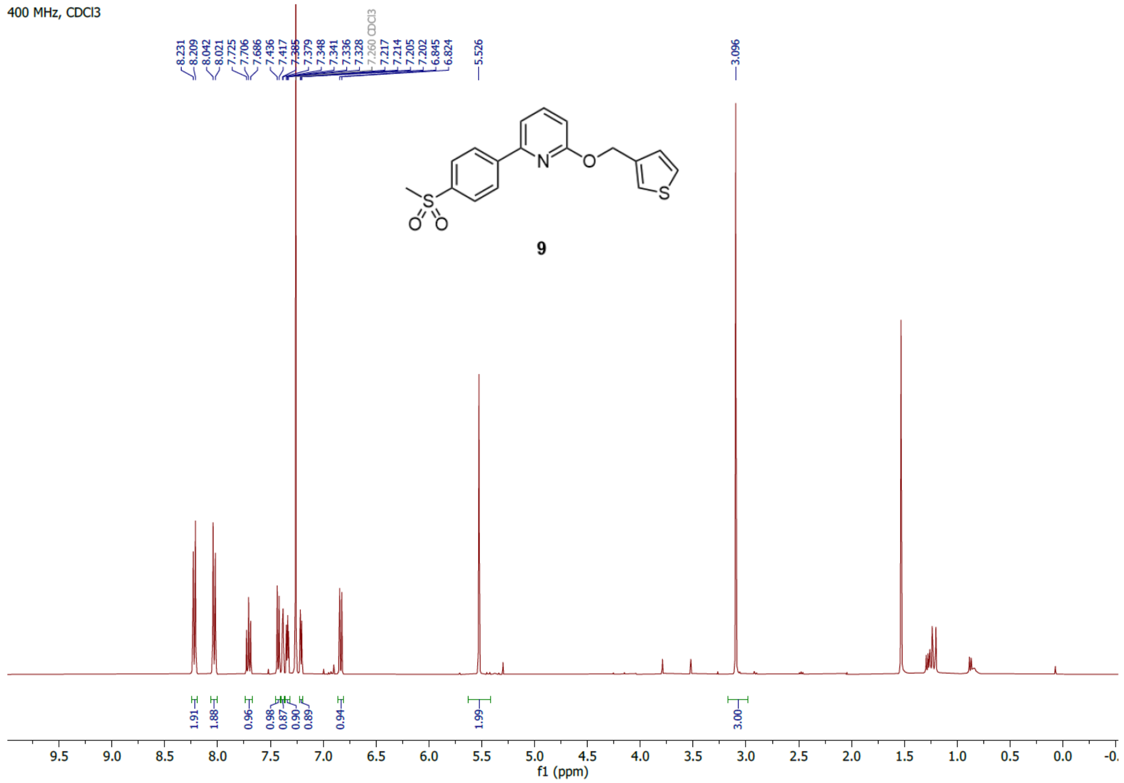

UPLC

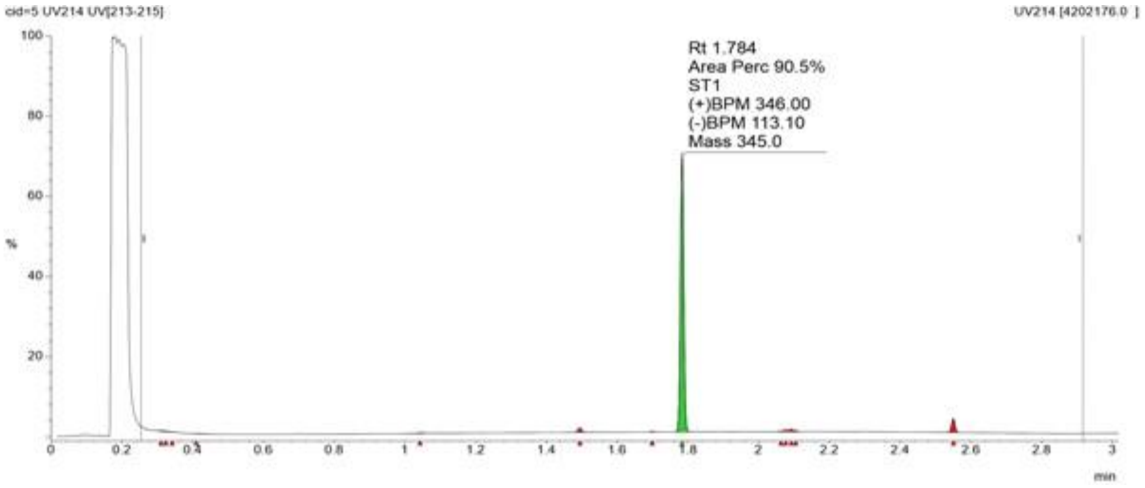

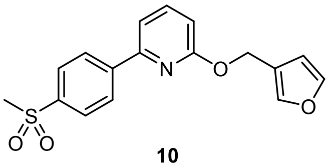

10

2-(furan-3-ylmethoxy)-6-(4-(methylsulfonyl)phenyl)pyridine (**10**): ^{1}H NMR (300 MHz, $CDCl_3$): δ 8.23 (dt, *J* = 12.5, 2.1 Hz, 2H), 8.03 (dt, *J* = 12.3, 2.0 Hz, 2H), 7.70 (dd, *J* = 8.2, 7.5 Hz, 1H), 7.55 (dd, *J* = 1.5, 0.8 Hz, 1H), 7.42 (m, 2H), 6.81 (dd, *J* = 8.2, 0.5 Hz, 1 Hz, 1H), 6.53 (dd, *J* = 2.6, 1.0 Hz, 1H), 5.39 (s, 2H), 3.09 (s, 3H). MS-ESI calc'd for $C_{17}H_{15}NO_4S$ $[M+H]^+$ 330.07, found 330.20.

^{1}H NMR

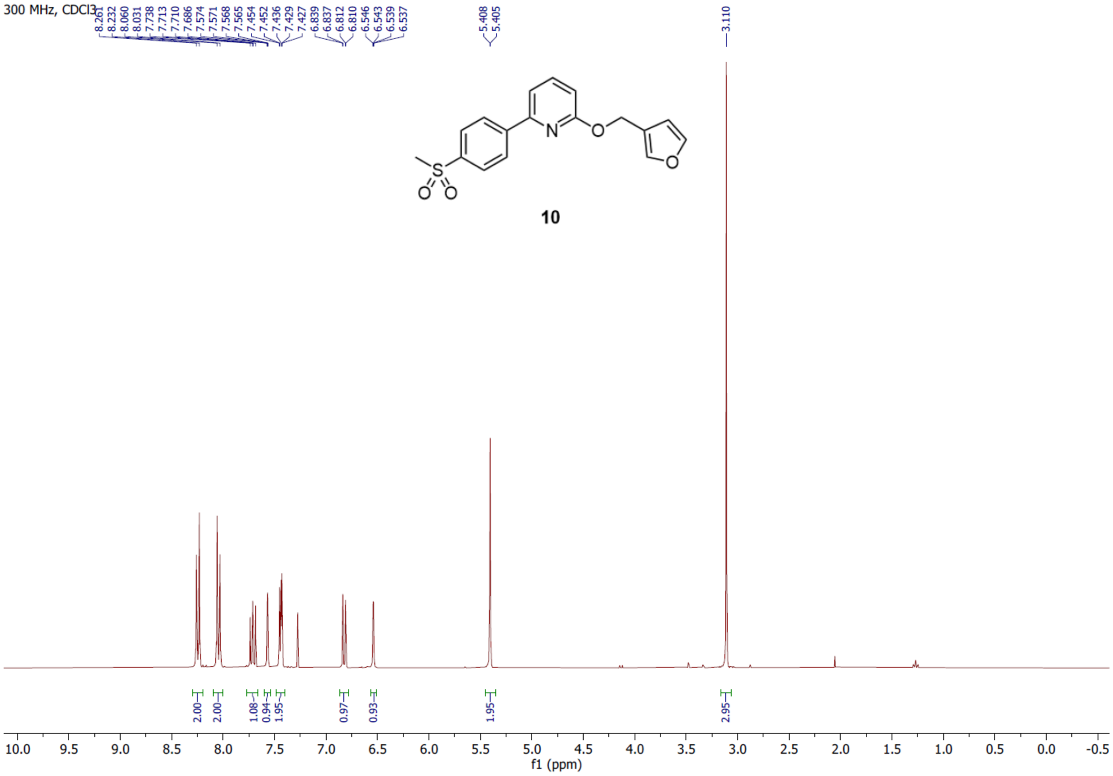

UPLC

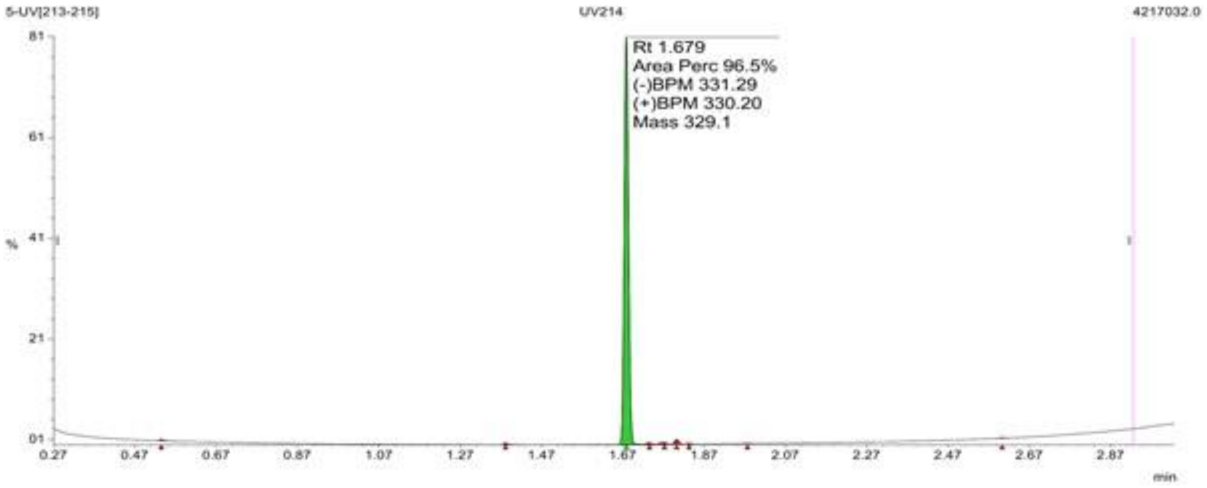

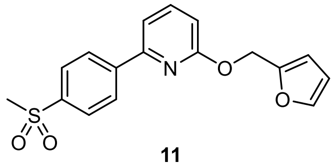

11

2-(furan-2-ylmethoxy)-6-(4-(methylsulfonyl)phenyl)pyridine (**11**): ^{1}H NMR (300 MHz, $CDCl_3$): δ 8.26 (d, *J* = 8.6 Hz, 2H), 8.05 (d, *J* = 8.6 Hz, 2H), 7.72 (t, *J* = 7.8 Hz, 1H), 7.47 (m, 2H), 6.84 (d, *J* = 8.3 Hz, 1H), 6.49 (m, 1H), 6.40 (dd, *J* = 3.3, 1.9 Hz, 1H), 5.49 (s, 2H), 3.12 (s, 3H). MS-ESI calc'd for $C_{17}H_{15}NO_4S$ $[M+Na]^+$ 352.06, found 352.11.

^{1}H NMR

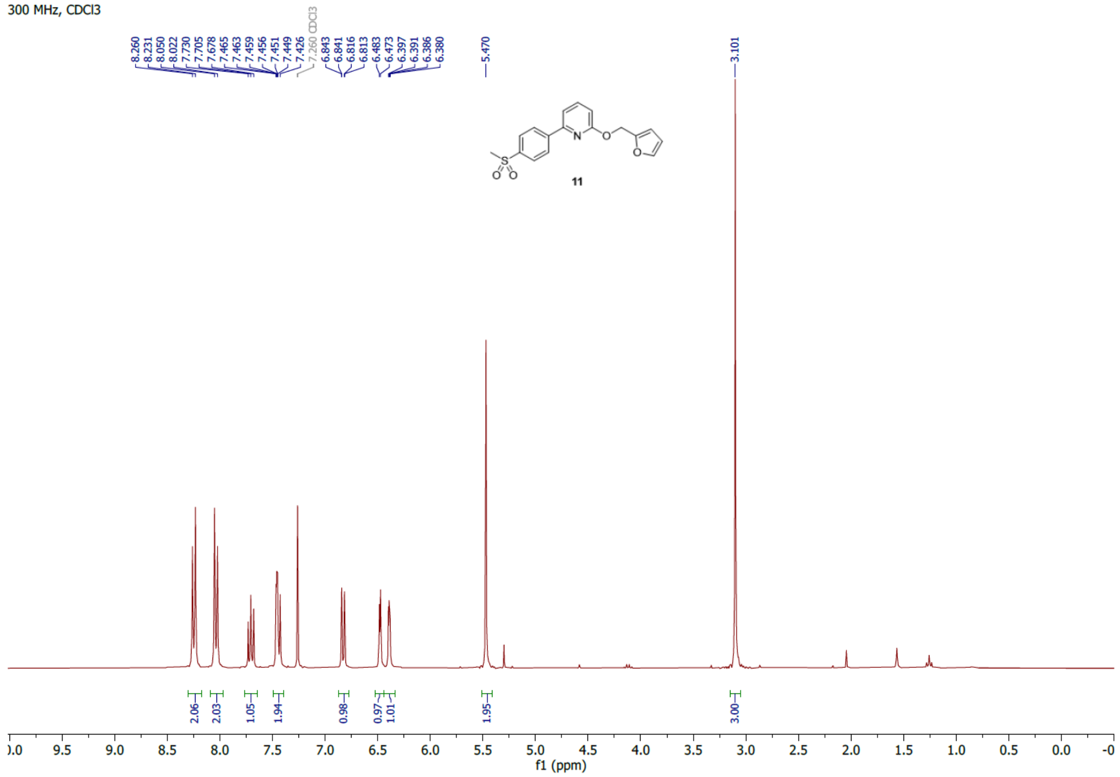

UPLC

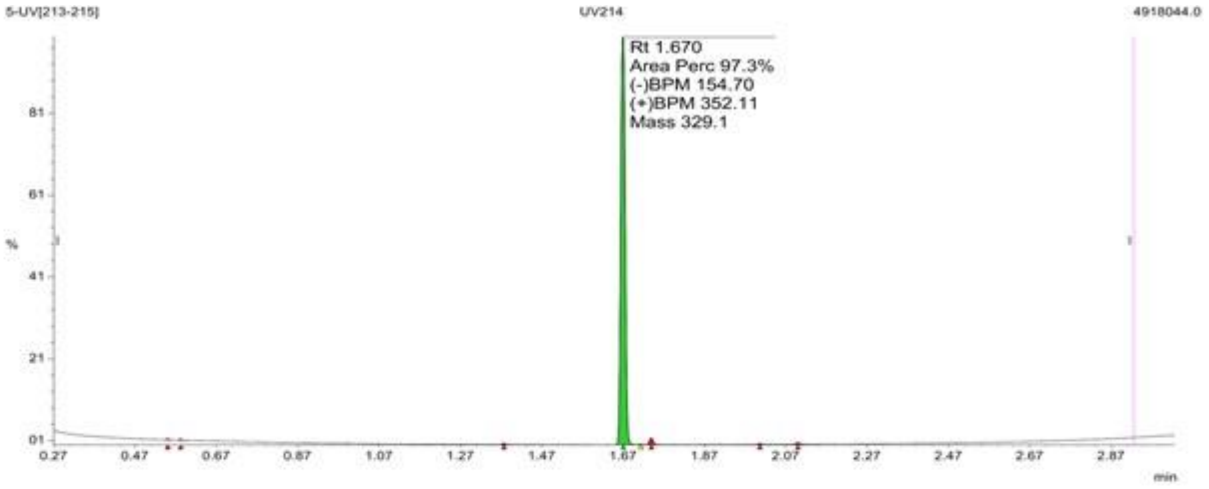

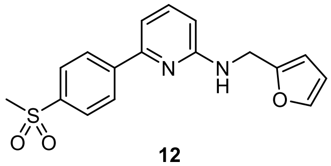

12

N-(furan-2-ylmethyl)-6-(4-(methylsulfonyl)phenyl)pyridin-2-amine (**12**): ^{1}H NMR (300 MHz, $CDCl_3$): δ 8.18 (d, *J* = 8.8 Hz, 2H), 7.99 (d, *J* = 8.7 Hz, 2H), 7.53 (dd, *J* = 8.3, 7.5 Hz, 1H), 7.37 (dd, *J* = 1.9, 0.9 Hz, 1H), 7.13 (dd, *J* = 7.5, 0.7 Hz, 1H), 6.48 (dd, *J* = 8.3, 0.7 Hz, 1H), 6.33 (dd, *J* = 3.2, 1.8 Hz, 1H), 6.27 (dd, *J* = 3.2, 0.9 Hz, 1H), 4.93 (t, *J* = 5.9 Hz, 1H), 4.63 (dd, *J* = 5.8, 0.8 Hz, 2H), 3.07 (s, 3H). MS-ESI calc'd for $C_{17}H_{16}N_2O_3S$ $[M+H]^+$ 329.10, found 329.04.

^{1}H NMR

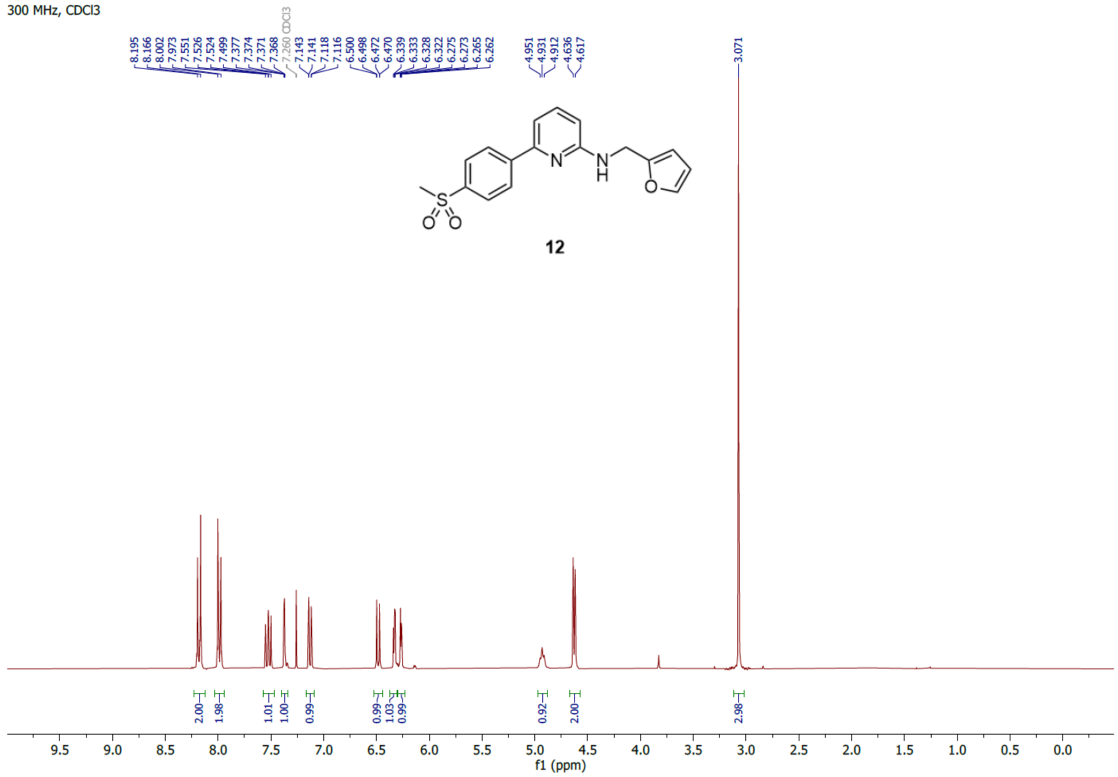

UPLC

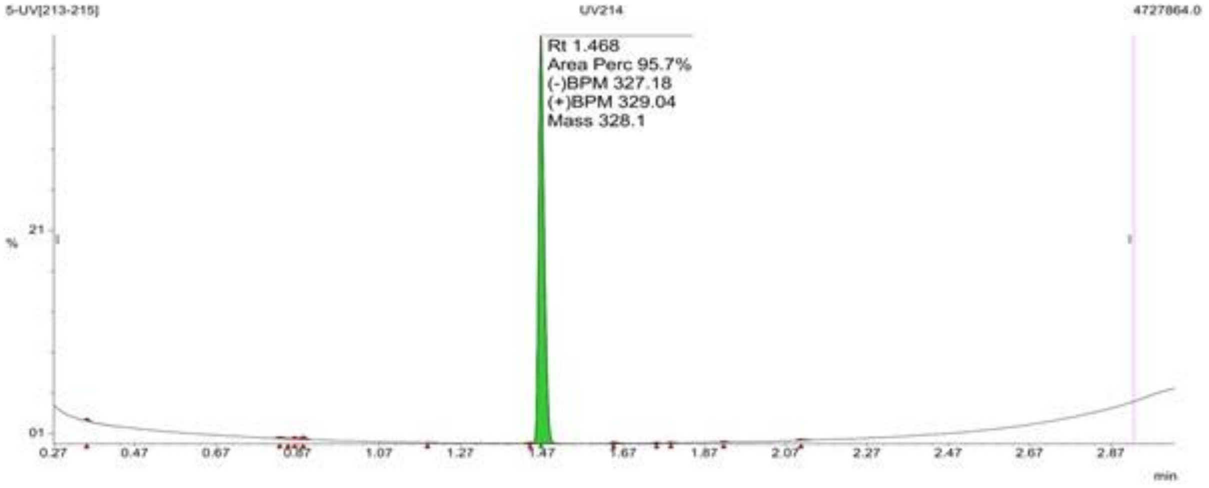

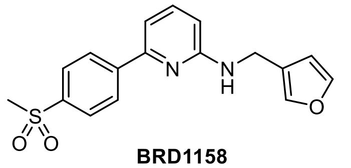

BRD1158

N-(furan-3-ylmethyl)-6-(4-(methylsulfonyl)phenyl)prydin-2-amine (**BRD1158**): ^{1}H NMR (400 MHz, $CDCl_3$): δ 8.18 (d, *J* = 8.7 Hz, 2H), 7.99 (d, *J* = 8.7 Hz, 2H), 7.54 (dd, *J* = 8.3, 7.5 Hz, 1H), 7.44 (dd, *J* = 1.6, 0.9 Hz, 1H), 7.40 (t, *J* = 1.7 Hz, 1H), 7.13 (dd, *J* = 7.5, 0.7 Hz, 1H), 6.46 (dd, *J* = 8.3, 0.7 Hz, 1H), 6.44 (dd, *J* = 1.9, 0.9 Hz), 4.78 (t, *J* = 5.7 Hz, 1H), 4.47 (d, *J* = 5.5 Hz, 2H), 3.07 (s, 3H). MS-ESI calc'd for $C_{17}H_{16}N_2O_3S$ $[M+H]^+$ 329.10, found 329.20.

^{1}H NMR

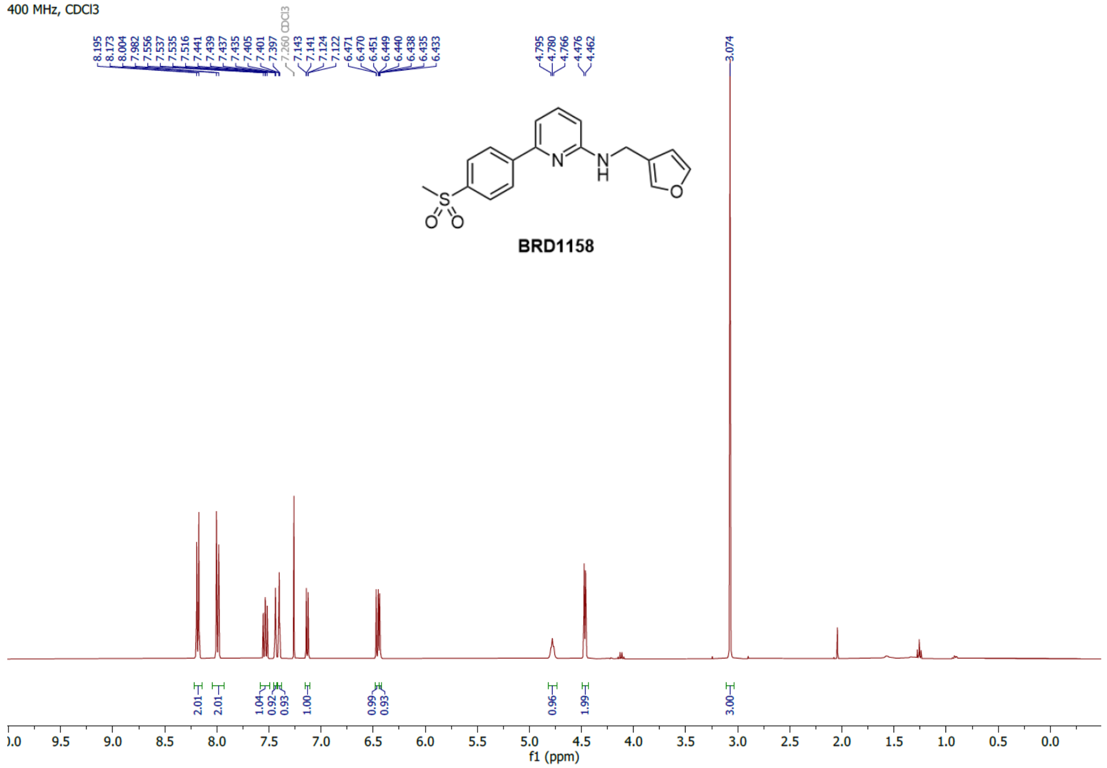

UPLC

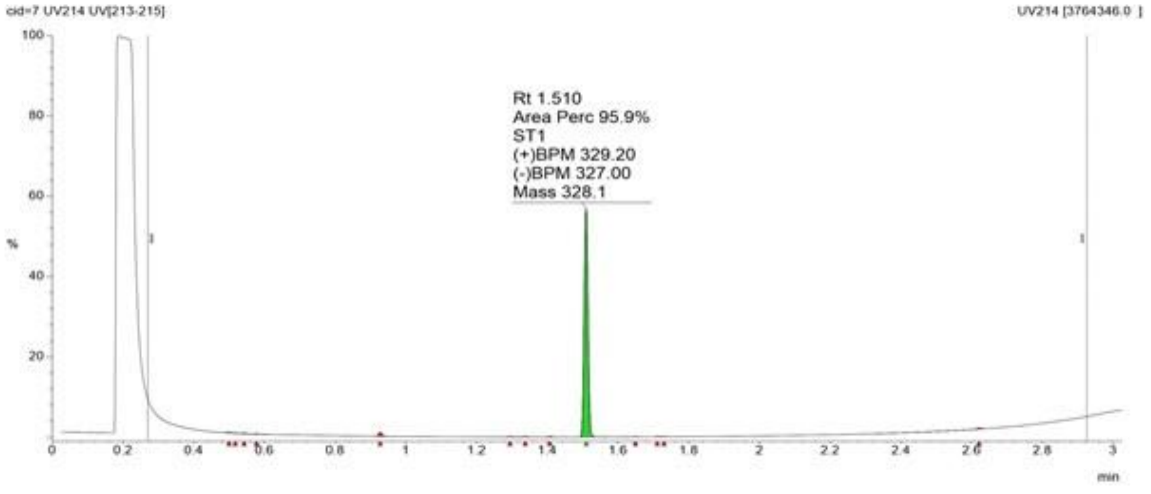

Synthesis of precursors and [^{11}C] radiolabeled MC1, BRD2369, and BRD1158

[^{11}C]MC1 was synthesized according to previously reported methods[3].

Synthesis of radiolabel precursors.

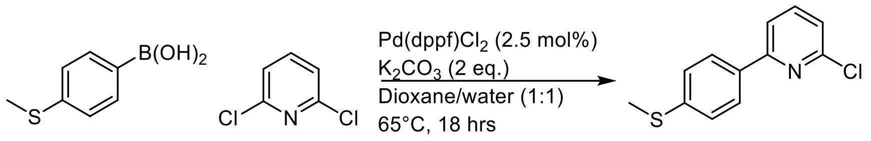

h

In a flask under nitrogen were dissolved 2,6-dichloropyridine (2.0 g, 13.5 mmol, 1.0 eq.), (4-(methylsulfanyl)phenyl)boronic acid (2.26 g, 13.5 mmol, 1.0 eq.), potassium carbonate (3.73 g, 27.0 mmol, 2.0 eq.) and Pd(dppf)Cl_2 (275 mg, 0.338 mmol, 2.5 mol%) in degassed 1,4-dioxane/water (1:1, 50 mL). The mixture was stirred for 18 hrs at 65°C, then brought back to room temperature. Water (65 mL) was added and the aqueous layer was extracted with DCM (3x100 mL). The combined organic layers were dried over $MgSO_4$, filtered and evaporated under reduced pressure. The crude material was purified by normal phase chromatography (EtOAc/hex, 0-15%) to afford 2-chloro-6-(4-(methylsulfanyl)phenyl)pyridine **h** (1.8328 g, 50.0 % yield) as a white solid.

2-chloro-6-(4-(methylthio)phenyl)pyridine (**h**): ^{1}H NMR (400 MHz, $CDCl_3$): δ 7.93 (d, *J* = 8.5 Hz, 2H), 7.68 (t, *J* = 7.7 Hz, 1H), 7.61 (dd, *J* = 7.8, 0.9 Hz, 1H), 7.32 (d, *J* = 8.5 Hz, 2H), 7.23 (dd, *J* = 7.7, 0.9 Hz, 1H), 2.53 (s, 3H). MS-ESI calc'd for $C_{12}H_{10}ClNS$ $[M+H]^+$ 236.03, found 236.10.

^{1}H NMR

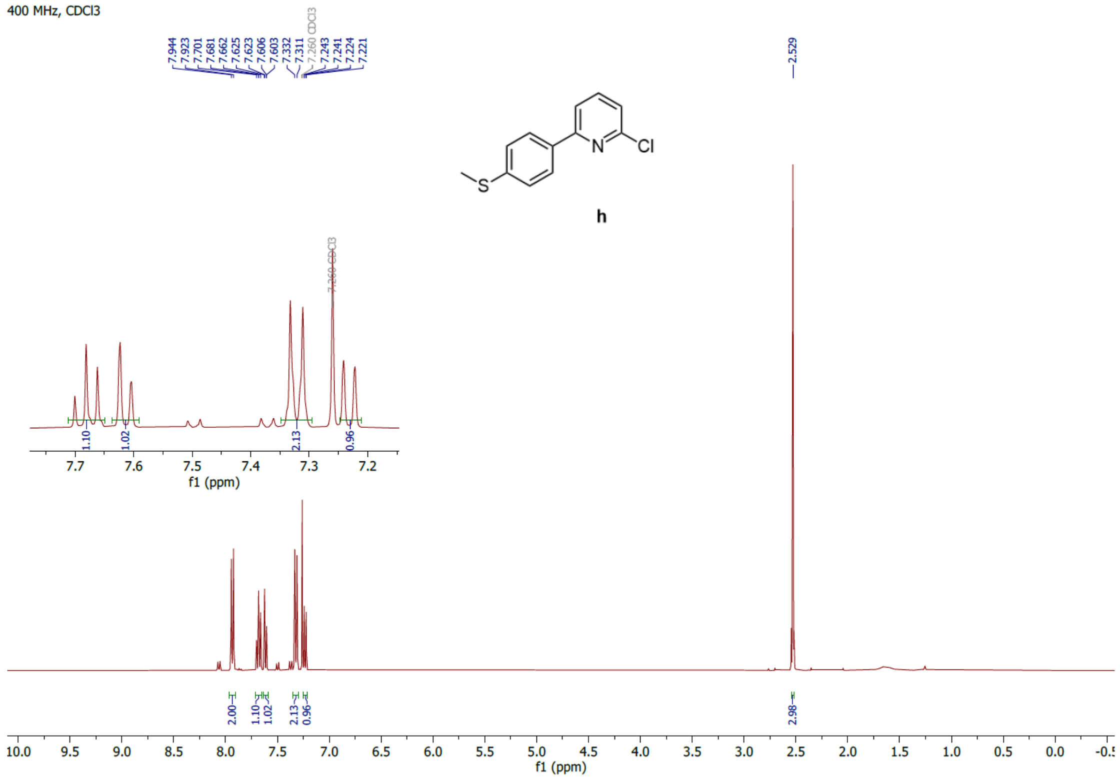

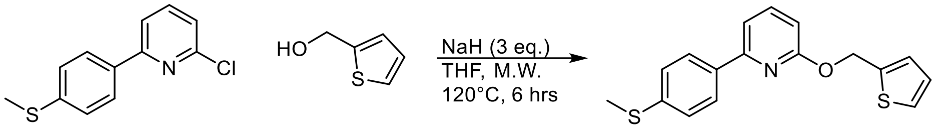

h **i**

In a flame-dried sealed tube under nitrogen, sodium hydride (152 mg, 60% in mineral oil, 3.81 mmol, 3.0 eq.) was dispersed in anhydrous THF (4.5 mL). (thiophen-2-yl)methanol (360 µL, 3.81 mmol, 3.0 eq.) was added dropwise and the solution was stirred at room temperature for 10 min. A solution of **h** (300 mg, 1.27 mmol, 1.0 eq.) in anhydrous THF (4.5 mL) was added and the solution was heated by microwave at 120 °C for 6 h. Water was added and extracted with DCM. The combined organic layers were dried over $MgSO_4$, filtered and evaporated. The crude material was purified by normal phase chromatography (hex/EtOAc, 0-5 %) to afford 2-(4-(methylthio)phenyl)-6-((thiophen-2-yl)methoxy)pyridine **I** (318.1 mg, 72.6 % yield) as a white powder.

2-(4-(methylthio)phenyl)-6-((thiophen-2-yl)methoxy)pyridine (**i**): ^1H NMR (400 MHz, $CDCl_3$) δ 8.02 (d, *J* = 8.5 Hz, 2H), 7.63 (t, *J* = 7.8 Hz, 1H), 7.36 – 7.33 (m, 3H), 7.29 (dd, *J* = 5.1, 1.2 Hz, 1H), 7.18 (d, *J* = 3.5 Hz, 1H), 6.99 (dd, *J* = 5.1, 3.5 Hz, 1H), 6.70 (d, *J* = 8.2 Hz, 1H), 5.68 (s, 2H), 2.54 (s, 3H). MS-ESI calc'd for $C_{17}H_{15}NOS_2$ $[M+H]^+$ 314.06, found 314.23.

^1H NMR

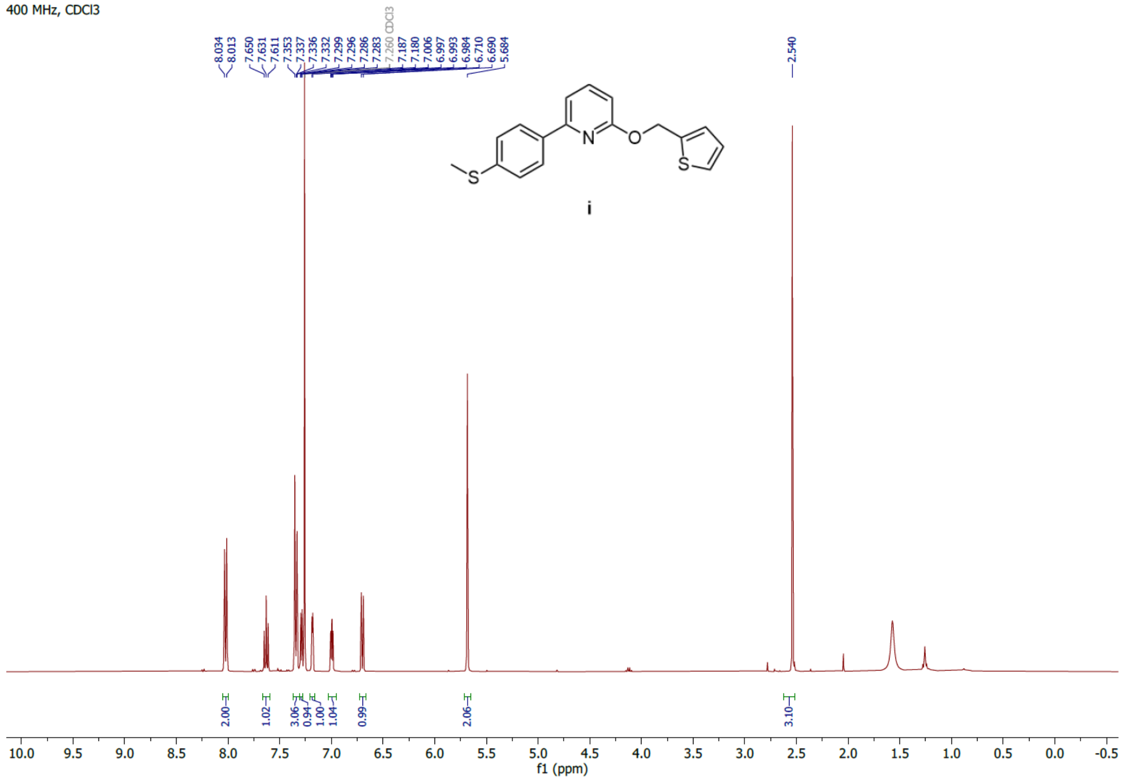

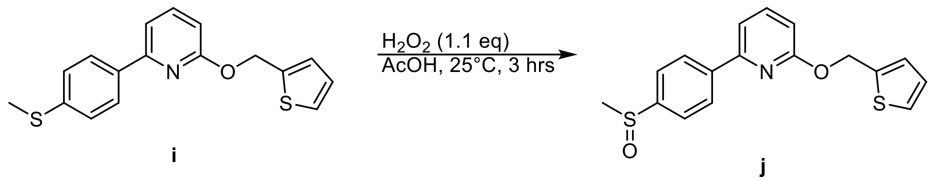

To a suspension of **i** (89 mg, 0.2839 mmol, 1.0 eq.) in acetic acid (1.1 mL) was added hydrogen peroxide (30%, 31.8 µL, 0.3122 mmol, 1.1 eq.). The mixture was stirred at room temperature for 3 h and the reaction mixture was poured into saturated aqueous $Na_2S_2O_3$, then neutralized by addition of saturated aqueous $NaHCO_3$. The aqueous suspension was extracted with CH_2Cl_2, and the combined organic layers were dried over Na_2CO_3, filtered, and evaporated. The crude mixture was washed a second time with saturated aqueous $NaHCO_3$ and extracted with DCM, dried over $MgSO_4$, filtered and evaporated to afford 2-(4-(methylsulfinyl)phenyl)-6-((thiophen-2-yl)methoxy)pyridine **j** (77.4 mg, 77.6 % yield).

2-(4-(methylsulfinyl)phenyl)-6-((thiophen-2-yl)methoxy)pyridine (**j**): ^{1}H NMR (400 MHz, $CDCl_3$) δ 8.24 (d, *J* = 8.4 Hz, 2H), 7.75 (d, *J* = 8.4 Hz, 2H), 7.69 (dd, *J* = 8.2, 7.5 Hz, 1H), 7.42 (d, *J* = 7.2 Hz, 1H), 7.30 (dd, *J* = 5.1, 1.2 Hz, 1H), 7.19 (dd, *J* = 3.5, 1.1 Hz, 1H), 7.00 (dd, *J* = 5.1, 3.5 Hz, 1H), 6.78 (d, *J* = 8.1 Hz, 1H), 5.69 (s, 2H), 2.78 (s, 3H). MS-ESI calc'd for $C_{17}H_{15}NO_2S_2$ $[M+H]^+$ 330.06, found 330.21.

^{1}H NMR

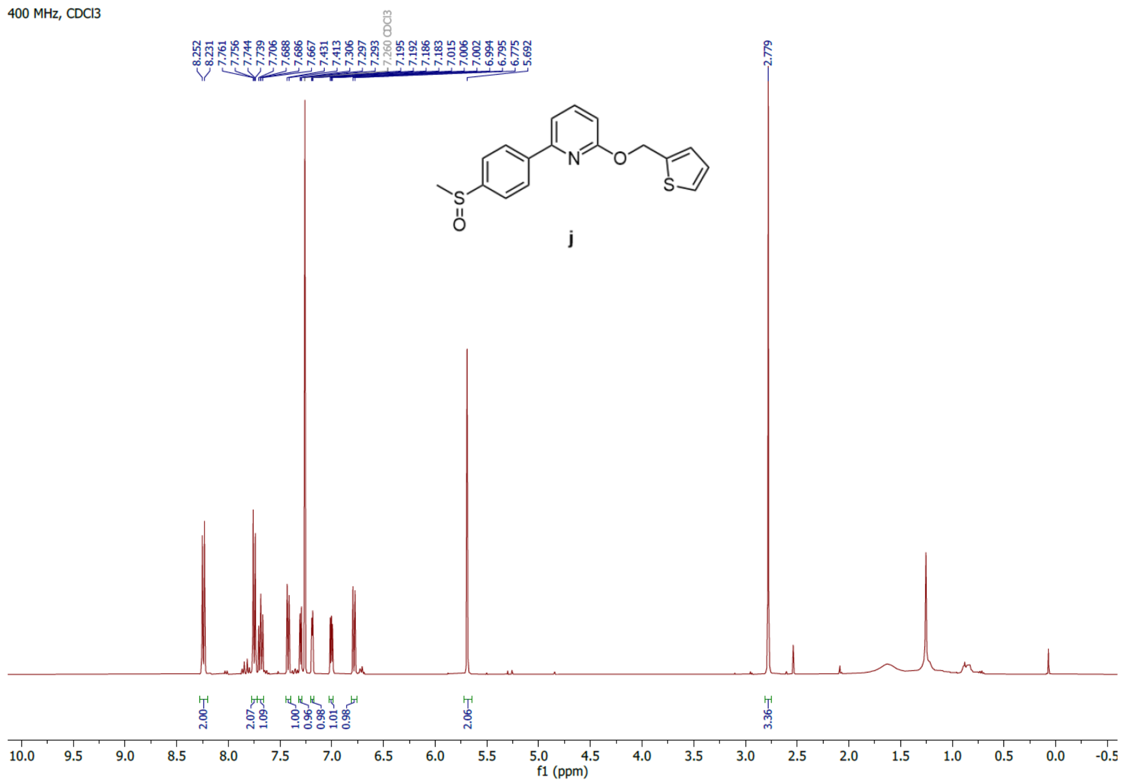

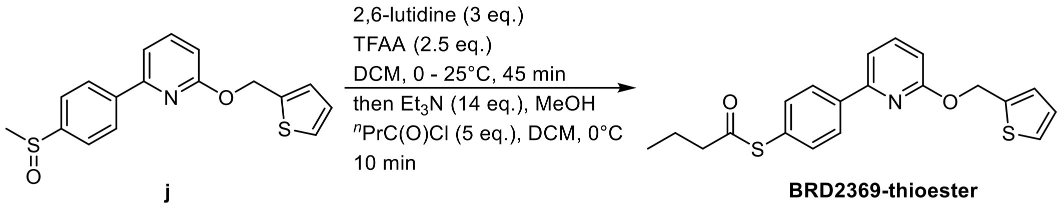

In a flask under nitrogen, to a solution of **j** (50 mg, 0.1517 mmol, 1 eq.) and 2,6-lutidine (52.6 µL, 0.4551 mmol, 3 eq.) in DCM (1 mL) at 0°C was added trifluoroacetic anhydride (52.6 µL, 0.3792 mmol, 2.5 eq.). The reaction mixture was allowed to warm to room temperature and stirred 45 min. The volatiles were then evaporated. After evaporation, the crude material was put under nitrogen and cooled down to 0°C and a mixture of triethylamine (150 µL, 1.07 mmol, 7.05 eq.) and methanol (20 µL, 0.4931 mmol, 3.25 eq.) was added. Stirring was continued for 5 min at 0°C. Additional triethylamine (150 µL, 1.07 mmol, 7.05 eq.) was added and the mixture was diluted with DCM (1 mL). After dropwise addition of butyryl chloride (78.7 µL, 0.7585 mmol, 5 eq.), the reaction was continued at 0°C for 3 min. Water was added and extracted with DCM. The combined organic layers were dried over $MgSO_4$, filtered and evaporated. The crude material was purified by normal phase chromatography (hex/EtOAc, 0-10 %) to afford *S*-(4-(6-(thiophen-2-ylmethoxy)pyridine-2-yl)phenyl)butanethioate **BRD2369-thioester** (36.6 mg, 65.3 % yield).

S-(4-(6-(thiophen-2-ylmethoxy)pyridine-2-yl)phenyl)butanethioate (**BRD2369-thioester**): ^{1}H NMR (400 MHz, $CDCl_3$) δ 8.12 (d, *J* = 8.3 Hz, 2H), 7.66 (t, *J* = 7.8 Hz, 1H), 7.51 (d, *J* = 8.3 Hz, 2H), 7.39 (d, *J* = 7.4 Hz, 1H), 7.29 (dd, *J* = 5.1, 1.2 Hz, 1H), 7.18 (d, *J* = 3.5 Hz, 1H), 7.00 (dd, *J* = 5.0, 3.7 Hz, 1H), 6.75 (d, *J* = 8.2 Hz, 1H), 5.68 (s, 2H), 2.67 (t, *J* = 7.4 Hz, 2H), 1.77 (h, *J* = 7.4 Hz, 2H), 1.02 (t, *J* = 7.4 Hz, 3H). MS-ESI calc'd for $C_{20}H_{19}NO_2S_2$ $[M+H]^+$ 370.09, found 370.31.

^{1}H NMR

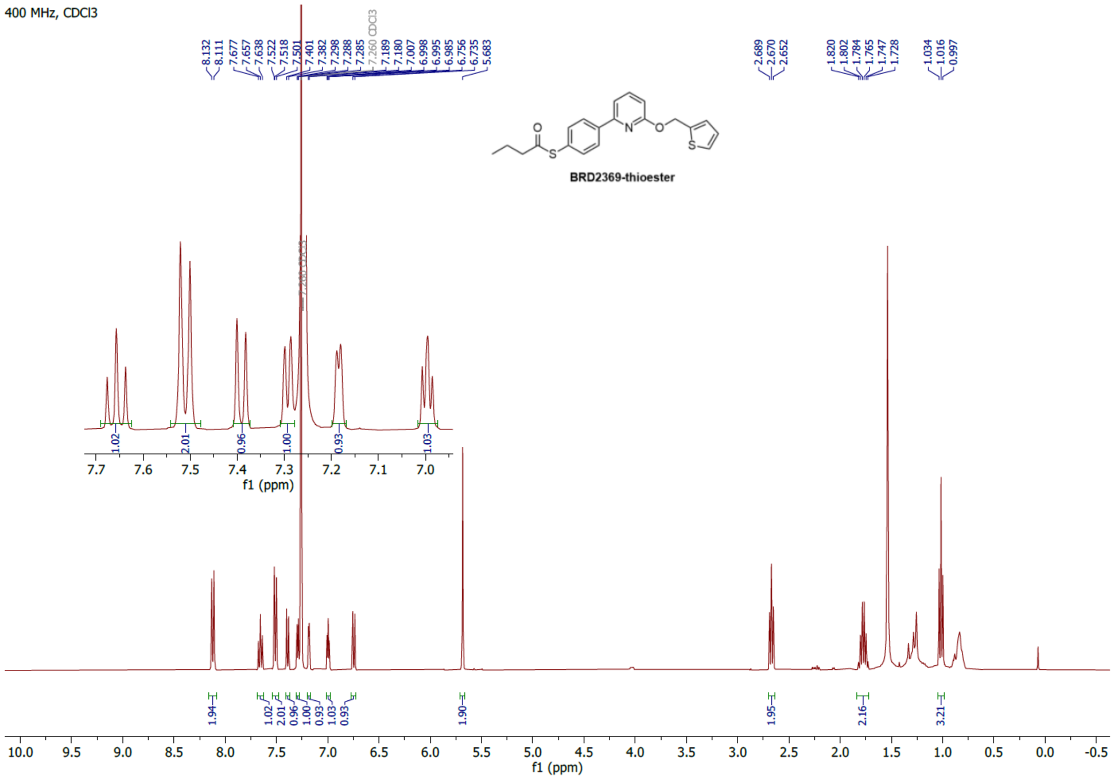

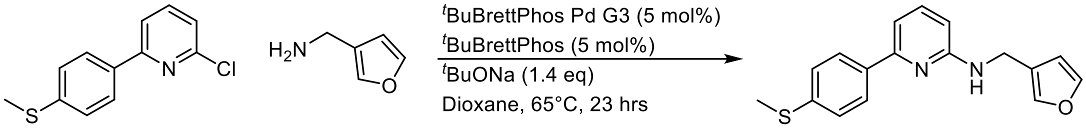

h **k**

In a flask under argon were added **h** (200 mg, 0.8484 mmol, 1 eq.), furan-3-yl-methanamine (82.3 mg, 0.8484 mmol, 1 eq.), tBuBrettPhos Pd G3 (36.2 mg, 0.04242 mmol, 0.05 eq.), ^{t}BuBrettPhos (20.5 mg, 0.04242 mmol, 0.05 eq.) and sodium ^{t}butoxide (113 mg, 1.18 mmol, 1.4 eq.) in dioxane (10 mL). The mixture was stirred at 60°C for 23hrs. The solvent was evaporated and the crude was purified by normal phase flash chromatography (Hex/EtOAc, 0-100%) to give *N*-((furan-3-yl)methyl)-6-(4-(methylthio)phenyl)pyridin-2-amine **k** (157.1 mg, 62.5 % yield) as a colorless oil.

N-((furan-3-yl)methyl)-6-(4-(methylthio)phenyl)pyridin-2-amine (**k**): ^{1}H NMR (400 MHz, $CDCl_3$) δ 7.93 (d, *J* = 8.5 Hz, 2H), 7.48 (t, *J* = 7.9 Hz, 1H), 7.43 (d, *J* = 0.7 Hz, 1H), 7.39 (t, *J* = 1.7 Hz, 1H), 7.31 (d, *J* = 8.5 Hz, 2H), 7.05 (d, *J* = 7.4 Hz, 1H), 6.44 (d, *J* = 2.2 Hz, 1H), 6.36 (d, *J* = 8.2 Hz, 1H), 4.70 (s, 1H), 4.44 (d, *J* = 5.3 Hz, 2H), 2.52 (s, 3H). MS-ESI calc'd for $C_{17}H_{16}N_2OS$ $[M+H]^+$ 297.10, found 297.63.

^{1}H NMR

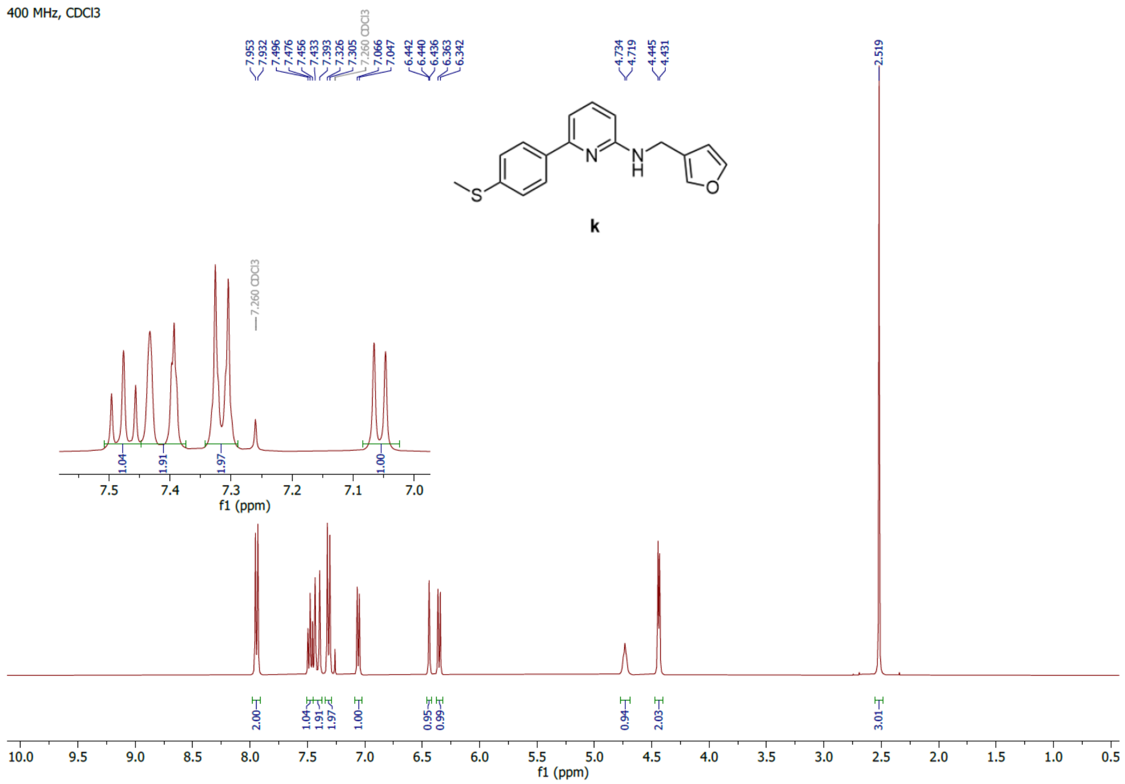

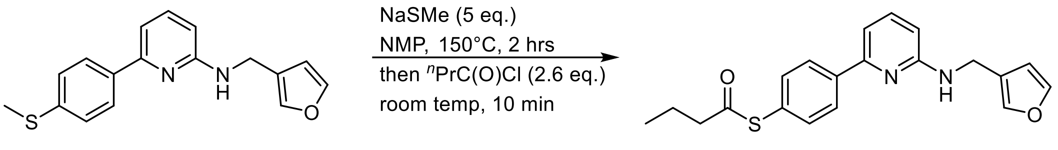

In a vial under nitrogen were dissolved **k** (95 mg, 0.3205 mmol, 1.0 eq.) and NaSMe (112 mg, 1.60 mmol, 5.0 eq.) in NMP (3 mL). The solution was stirred at 150 °C for 2hrs, then brought back to room temperature. Butanoyl chloride (66.4 µL, 0.641 mmol, 2.0 eq.) was added and the solution was stirred for 10 min. The reaction was quenched with a 5% solution of aqueous LiCl, which was then extracted with EtOAc. The combined organic layers were washed with 5% aqueous LiCl, dried over MgSO4, filtered and evaporated. The crude material was purified by normal phase chromatography (hex/EtOAc, 0-10%) to afford a colorless oil. Water was added and the solution was frozen and lyophilized to remove the remaining NMP. *S*-(4-(6-((furan-3-ylmethyl)amino)pyridine-2-yl)phenyl)butanethioate **BRD1158-thioester** (47.7 mg, 42.5% yield) was thus afforded as a white solid.

S-(4-(6-((furan-3-ylmethyl)amino)pyridine-2-yl)phenyl)butanethioate (**BRD1158-thioester**): ^{1}H NMR (400 MHz, $CDCl_3$) δ 8.03 (d, J = 8.4 Hz, 2H), 7.51 (d, *J* = 7.6 Hz, 1H), 7.48 (d, *J* = 8.4 Hz, 2H), 7.43 (dt, *J* = 1.7, 0.9 Hz, 1H), 7.39 (t, *J* = 1.7 Hz, 1H), 7.09 (dd, *J* = 7.5, 0.7 Hz, 1H), 6.44 (dd, *J* = 1.8, 0.9 Hz, 1H), 6.40 (d, *J* = 8.0 Hz, 1H), 4.73 (br s, 1H), 4.48 (d, *J* = 4.8 Hz, 2H), 2.65 (t, *J* = 7.4 Hz, 2H), 1.76 (q, *J* = 7.4 Hz, 2H), 1.01 (t, *J* = 7.4 Hz, 3H). MS-ESI calc'd for $C_{20}H_{20}N_2O_2S$ $[M+H]^+$ 353.13, found 353.14.

^{1}H NMR

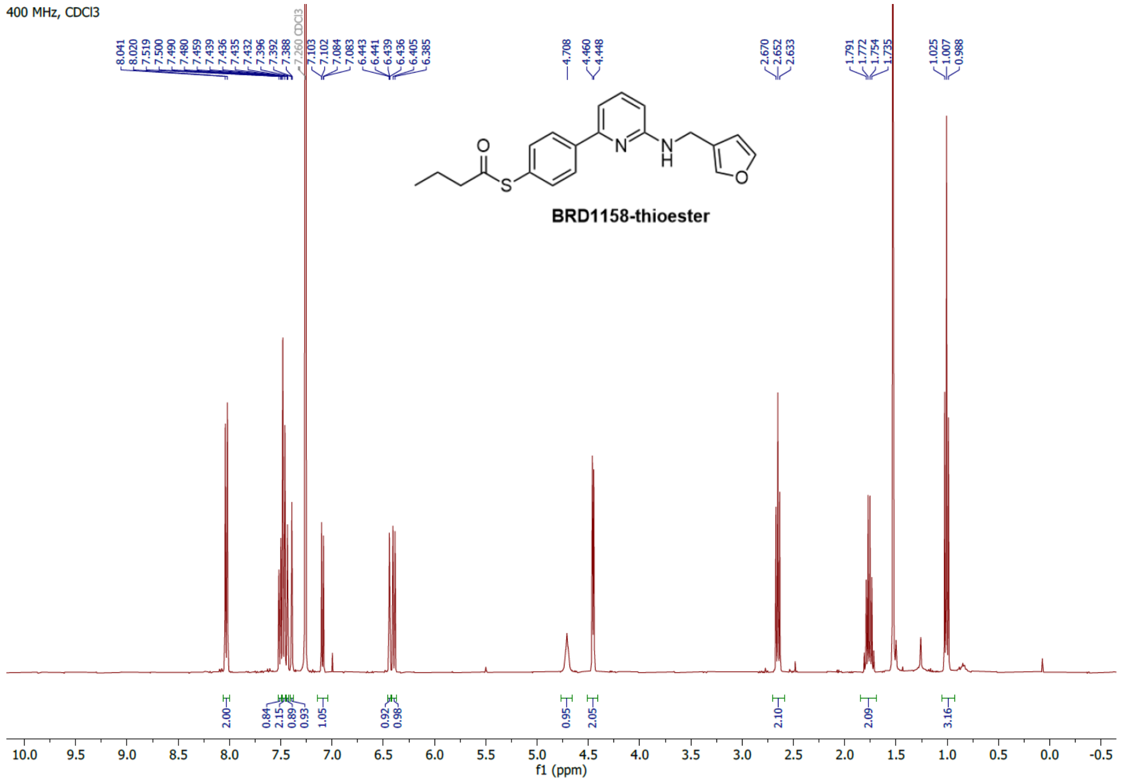

General synthesis for radiolabeling of thioester precursors.

To a solution of thioester butyrate precursor (5.41 µmol) in anhydrous DMF (0.3 mL) was added 0.1 mL of a 10.8 mM solution of pyrrolidine in DMF (1.08 µmol of pyrrolidine; 0.2 eq.). The mixture was vortexed for 3 min at ambient temperature. To this mixture, $[^{11}C]CH_3I$ carried by Helium gas was bubbled into the solution until radioactivity peaked (approximately 3 min). The mixture was stirred at room temperature for 1 min before the addition of 0.5 mL of a 108 mM oxone suspension (1:2 MeOH:Water). The mixture was stirred at 50°C for 3 min. 0.8mL of water was added and the mixture was purified by semi-preparative HPLC (9.4 x 250 mm C-18, Agilent eclipse plus xdb, 5 µm). The collected fraction from HPLC was diluted with 20mL sterile water, passed through C18 sep pak plus. The sep pak was washed with 5mL sterile water. The product was eluted with 1mL ethanol then 9 mL 0.9% saline and passed through a sterile filter (0.22 µm) into a sterile vial ready for injection.

Synthesis of **[^{11}C]BRD2369**

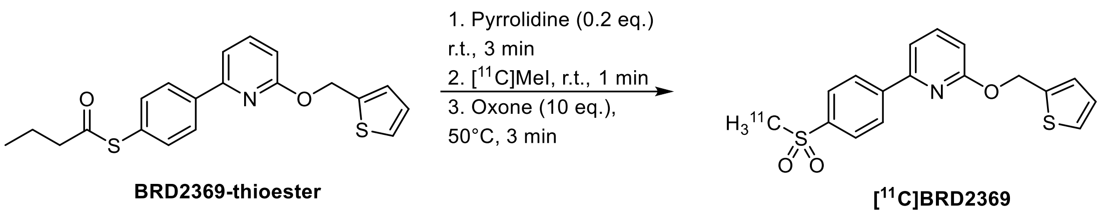

Semi-preparative HPLC: Isocratic 0-12 min at 50% MeCN / 49.9% H_2O / 0.1% formic acid at 5mL/min. Linear Gradient from 12-17min (50%MeCN/49.9% H_2O/0.1%FA to 95% MeCN/4.9%H_2O/0.1%FA). The product eluted at 16 min.

Decay Corrected Radiochemical Yield from [^{11}C]CH_3I trapping in reaction vial to final sterile filtered product: 22%

Semi-preparative HPLC Radioactivity Chromatogram:

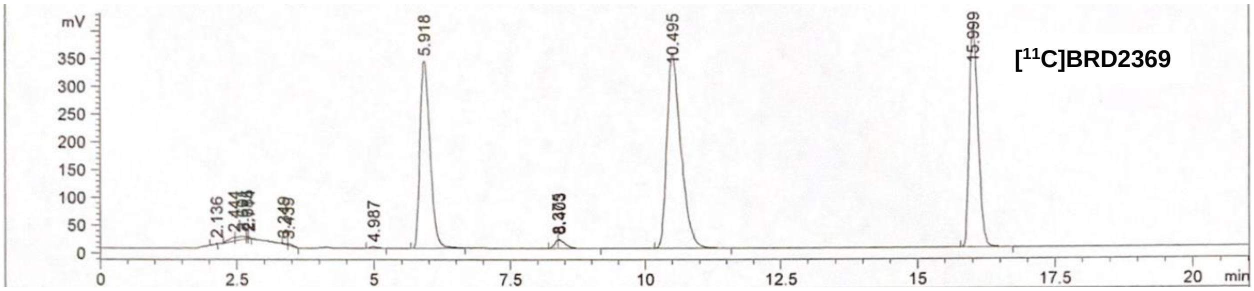

Semi-preparative HPLC UV (254 nm) Chromatogram:

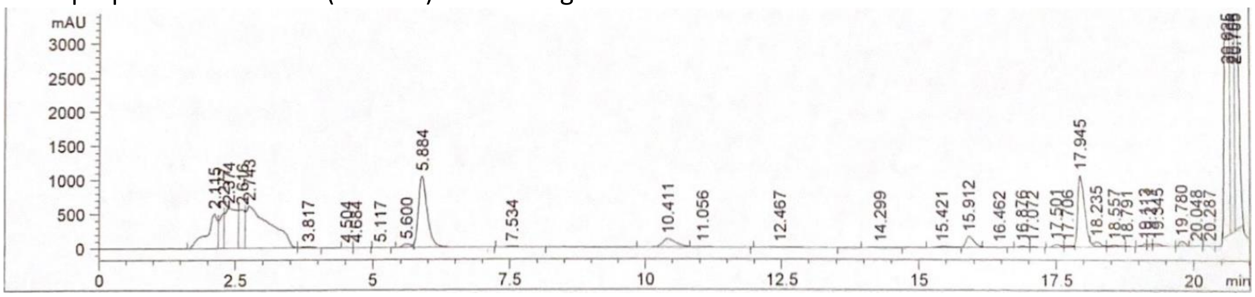

Synthesis of **[^{11}C]BRD1158**

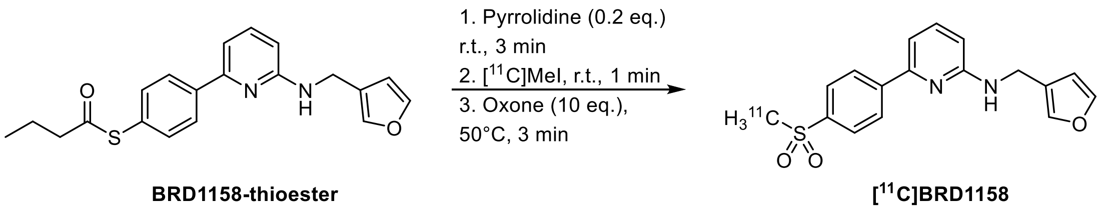

Semi-preparative HPLC: Isocratic, 35% MeCN / 64.9% H_2O / 0.1% formic acid, 5mL/min. The product eluted at 8.3 min.

Decay Corrected Radiochemical Yield from [^{11}C]CH_3I trapping in reaction vial to final sterile filtered product: 53%

Semi-preparative HPLC Radioactivity Chromatogram:

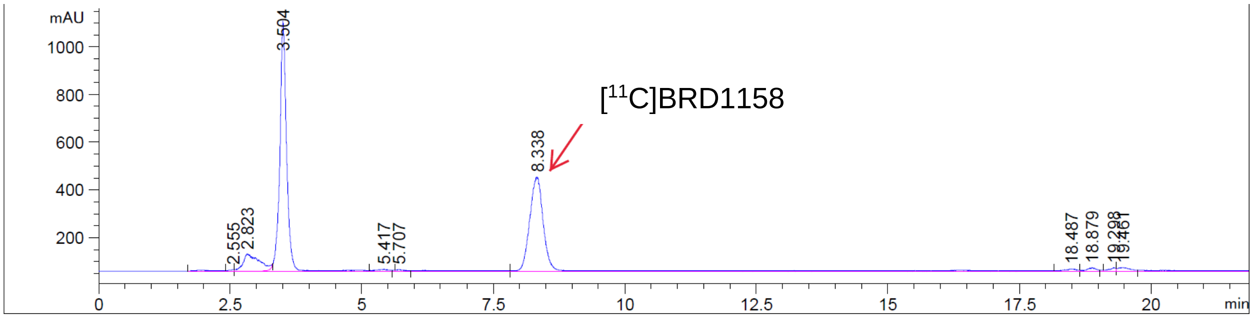

Semi-preparative HPLC UV (254 nm) Chromatogram

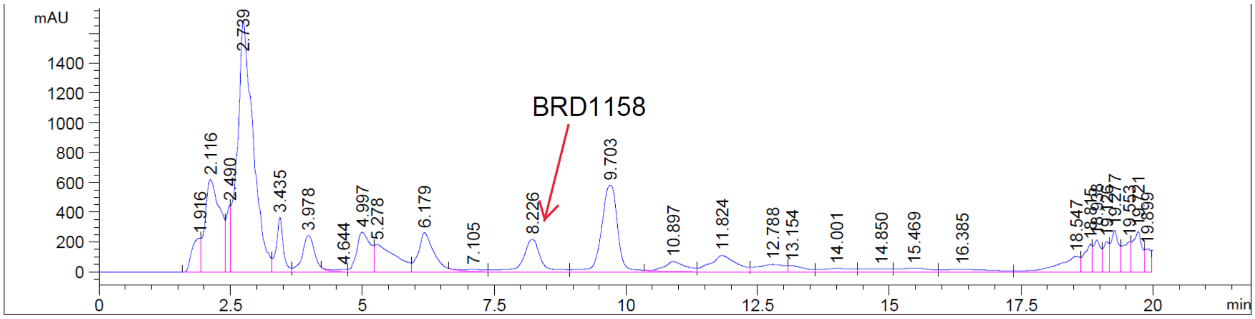

Animals

In accordance with Massachusetts General Hospital Institutional Animal Care and Use Committee (IACUC) protocols, Sprague Dawley rats (300-500g, Charles River Laboratories), wildtype mice (Strain C57BL/6J), and human Thy-1-COX-2 transgenic line 303 mice (Strain 010703, Jackson Laboratories) were used in these studies. All animals were housed in a standard animal facility with access to conventional rodent food and water ad libitum and a 12-hour day/night schedule. All animals were allowed to acclimate before any experimental procedures. Only male animals were used for imaging experiments.

Animal Preparation

Animals were induced under anesthesia using 1-3% isoflurane in a carrier of 1.0-1.5 L/min oxygen. Maintenance of isoflurane depended on procedure and species, rats 2-3% and mice 1-2%. Rats undergoing surgeries had higher isoflurane maintenance at 3-4%. After induction, the animals' body temperatures were regulated using a support heating pad or fan throughout all experiments. Respiratory rates and body temperature were monitored through all imaging and surgical procedures, generally using a respiratory pad and rectal probe. Injections were made through the lateral tail vein. Rats were cannulated with an IV extension using a 60 in, 0.3 mL volume IV extender (Smith Medical) and a 24 ga x 0.75 in IV catheter (McKesson). Mice were cannulated with Intramedic PE10 tubing (BD Biosciences).

AAV Rat Experimental Groups, Drug Formulation and Surgical Procedures

Pairs of male rats were placed into experimental groups to receive unilateral intracerebroventricular (ICV) injections of AAV2-GFP-hCOX2 (6.53E+12gc/mL) as we developed an injection paradigm that achieved robust, localized, unilateral overexpression of human COX-2. Group 1: Animals received ICV injections of 3µL of AAV2-GFP-hCOX2 to the right caudate putamen (Rcpu) at 3 depths (9µL total). The contralateral site, left caudate putamen (Lcpu), received 3µL at 3 depths (9µL total) of AAV2-GFP as a control. COX-2 expression in Group 1 was confirmed with IHC to give confidence that our experimental paradigm was resulting in COX-2 overexpression (SI Figure 7). Group 2: Animals received one intraperitoneal (IP) injection of mannitol (10 mL/kg dose) fifteen minutes prior to AAV injection to increase vector spread and gene expression as described in Mastakov et al. 2001[4]. Group 2 animals then received one 4µL ICV injection of AAV2-GFP-hCOX2 to the Rcpu and one 4µL ICV injection of AAV2-GFP to the Lcpu, an injection paradigm that successfully led to robust, localized, unilateral COX-2 expression that we could use to compare radiotracers (Figure 2, SI Figure 8). Another rat (animal ID SD2006080) received one 4µL ICV injection of AAV2-GFP-hCOX2 to the Rcpu and no left caudate sham injection (SI Figure 9). All surgical procedures were performed aseptically. Once rats were prepared and secured with a stereotaxic device (Stoelting Co, Wood Dale, IL), bregma was located. Using a sterile ruler coordinates ML 2.5 mm; AP 0.5 mm was measured and marked respective to bregma. A sterile drill was then used to make a small hole at these coordinates to expose the brain cavity. A 33-gauge neuro syringe (Hamilton, Reno, NV) was inserted around DV 3.5 mm for one injection. Injections were given slowly and the needle was kept in for 15 min to allow for adequate perfusion. For Group 1 rats who received doses across three depths, the deepest one would be injected and allowed to perfuse for 15 min first; then the next

deepest would be injected and allowed to perfuse for 15 min until all three injections were completed. In some experimental groups, mannitol was injected intraperitoneally (IP) or co-administered with the virus to allow for better perfusion, as suggested in previous literature[4]. After the surgical procedure was completed, animals were given enough time to recover (generally 28 days post-surgery) to facilitate cellular transduction with the AAV as well as subsequent expression of COX-2 and GFP, before subsequent imaging or IHC interrogation was conducted.

Rodent PET/MRI Acquisition

All PET and MRI experimental procedures were acquired on a 4.7 Tesla PET-MRI scanner using a B-GA20S HP gradient coil with a PET Insert Si 198 (Bruker Biospin, Billerica, MA). Custom 3D-printed cradles were used to secure anesthetized animals in the scanner. Rat and mice experimental groups were imaged separately. For rats, one animal was placed head-prone, and the other was placed tail-prone, aligning their heads in the scanner. For rat blocking experiments celecoxib was formulated in 20% ethanol, 30% polyethylene glycol and 50% saline and diluted appropriately to administer 1 mL/kg. For mice, up to six mice were inserted, two head prone and four tail prone. ParaVision 6 (Bruker) was used to acquire MR images, and ParaVision 360 (Bruker) was used to obtain PET images. A T2-weighted TurboRare pulse sequence was acquired with the following parameters: TR=8000 ms, TE=11 ms, RARE factor of 8, Spatial Resolution= 0.25 x 0.25 µm, Slice Thickness= 1 µm. Data from a 61 minute dynamic PET imaging scan acquired in list-mode and were reconstructed using maximum likelihood expectation maximization (MLEM) algorithm with 12 iterations, 0.75 mm isotropic voxels, and binned using time frames with durations of 8 x 15s, 6 x 30s, 6 x 60s, 10 x 120s, 6 x 300s.

Rodent Image Analysis

After co-alignment, PET and MR images were exported to DICOM files from ParaVision 360. Using PMOD software (Version 3.9, PMOD Technologies, Zurich, Switzerland), T2 Images were manually aligned with the appropriate rodent brain template, Schiffer rat brain (Px Rat) or Mouse Ma Benveniste Mirrone mouse brain using PMOD. The PET images were then aligned to the new T2 images using PMOD, creating an aligned data series for each animal. Representative MR images from PMOD templates were used in figures. For naïve rats, SUV average images were created for frames 1-9, minutes 0-3 to assess brain uptake, and for frames 17-22, minutes 10-22 to estimate endogenous COX-2 expression. For the AAV2-GFP-hCOX2 rats, three manual VOIs were placed using the Rcpu hotspot and the anatomical MR as visual aids. The first VOI was the Rcpu/AAV2-GFP-hCOX2 injection site which was manually placed to the hotspot in baseline scans, this was mirrored to the contralateral hemisphere for the Lcpu/AAV2-GFP sham injection site VOI. The cerebellum/reference region VOI was manually placed based on the anatomical MR. The same VOI sets (size and locations of VOIs) were used across baseline and block scans for the same animal. The same VOI sizes were used across experimental groups. SUV average PET images were created in PMOD and average time ranges for each tracer were based on the optimal portions of scans; for [^{11}C]BRD1158 SUV averages were created for frames 19-25/minutes 9-21 (12 minutes total), for [^{11}C]MC1 and [^{11}C]BRD2369 SUV averages were created for frames 30-32/minutes 29-41 (12 minutes total). For mouse scans, SUV average images were created for frames 25-36, minutes 20-

60. Skull stripping was performed on MR and PET images using the appropriate rodent template from PMOD. Spatial smoothing with a Gaussian kernel was performed on all rat images (1.2 x 1.2 x 1.2 full width at half maximum (FWHM)), and on all mouse images (1.1 x 1.1 x 1.1 FWHM). SUV data was generated for all relevant regions, in one of two ways depending on the parent PMOD file format, with same outcome values: Activity data, in kBq/cc, was imported from PMOD non-SUV files to Excel and converted into SUV data using a dose calibration calculation in Excel, or in PMOD, kBq/cc was converted into SUV and then exported to Excel. Both [^{11}C]MC1 and [^{11}C]BRD2369 were especially lipophilic and stuck to the injection lines and filters. To account for this loss in the injected dose, we performed a test to determine the volume of tracer lost to lines/filters during injection and then performed a line dose correction calculation on all [^{11}C]MC1 and [^{11}C]BRD2369 data. Time activity curves of SUV data and additional plots were generated using GraphPad Prism 9 (Dotmatics, Boston, MA).

Immunoblotting

Microdissected mouse and human brain tissue was flash frozen on dry ice. Frozen tissue was then transferred into a hepes based lysis buffer (25 mM hepes, 0.1 M NaCl, 1% Triton X100) containing complete protease inhibitor cocktail (Millipore Sigma) and a 5 mm stainless steel bead was subsequently used to homogenize the tissue using the TissueLyser II system (Qiagen) for 2x 5 min at 20 Hz. Lysates were then spun for 10 min at 15,000 g in a benchtop centrifuge to pellet insoluble material before the supernatant was removed to a new tube and protein concentration assayed using the BCA protein assay kit (Pierce) according to the manufacturer's instructions. Protein samples (20 µg) were then mixed with 2x Laemmli buffer (Bio-Rad) before being loaded onto 4-15% Criterion TGX Stain-Free Gels (Bio-Rad) and separated for 1 h at 150 V by SDS-PAGE. Separated proteins were transferred onto Immobilon-P PVDF membranes (Millipore IPVH00010) which were subsequently blocked in 5% milk PBS for 1 h at room temperature before being probed with antibodies to COX2 (Abcam ab15191 used @ 1/500) or GAPDH (Abcam ab181603 used @ 1/1000) O/N at 4°C in 5% milk PBS. Membranes were then washed for 3x 5 min in PBS before being incubated with relevant secondary antibodies (Promega W4021 and W4011) diluted 1/10000 in 5% milk PBS for 1 h at room temperature. After washing again for 3x 5 min in PBS bands were visualized with luminol-based enhanced chemiluminescence HRP substrate (Super signal west dura, Thermo Fisher Scientific) and the ImageQuant LAS 4000 system (GE healthcare) avoiding saturation of any pixels. To strip membranes and re-probe with a different antibody ReBlot Plus strong antibody stripping solution (EMD Millipore) was employed according to the manufacturer's instructions prior to re-blocking in 5% milk and staining. For quantification, the intensity of bands generated by staining was normalized to total protein staining (employed as a loading control) in the same lane. ImageJ software was used to carry out densitometry analysis of all bands.

Immunohistochemistry on rodent tissue sections

Immunohistochemistry was performed on mice and rats as described in Schafer et al., 2012 and Lehrman et al., 2018[5,6]. Briefly, brains were harvested following transcardial perfusion with 15ml PBS and 15ml 4% paraformaldehyde (PFA). Tissue was then postfixed in 4% PFA for 2 h, before being washed in PBS and transferred to a 30% sucrose solution for approximately 24-48hr. Subsequently tissue samples were embedded in a 2:1 mixture of 30% sucrose-PBS: Tissue-Tek O.C.T. Compound (Electron Microscope Sciences) and stored at -80°C. 25 µm cryosections were then cut using a Leica cryostat and affixed to Leica

Surgipath X-tra slides before being processed for immunohistochemistry as follows. Slides were heated for 10 min at 35°C followed by 3 rinses in PBS. They were then blocked with 10% normal goat serum and 0.3% Triton-X 100 solution (Sigma) for 1 h, before being incubated with primary antibodies overnight (O/N) at 4°C in 5% normal goat serum (Sigma) and 0.3% Triton X-100 solution (Sigma). The following antibodies were used at the noted dilutions: COX2 (Abcam ab15191) used @ 1/500; Iba-1 (Wako Chemicals 019-1741) used @ 1/500; Flag (Cell Signaling Technology 14793S) used @ 1/500; GFP (Aves GFP-1020) used @ 1/500. Slides were then washed for 3x 15 min in PBS followed by incubation with appropriate secondary antibodies (Alexa Fluor conjugates, Thermo Scientific used @ 1/500) in 5% normal goat serum for 2 hr at room temperature. Slides were then washed for 3x 15 min in PBS and mounted with Vectashield with DAPI (Vector Laboratories H-1000).

Immunohistochemistry using postmortem human tissue sections

Postmortem human tissue was fixed according to the procedures outlined in Waldvogel et al., 2008[7]. Briefly, the brain was extracted, and the basal and internal carotid arteries identified. The tissue was subsequently perfused by attaching winged infusion needles to these arteries and flowing through a solution of PBS containing 1% sodium nitrite followed by 3 liters of a fixative containing 15% formalin. After perfusion, the brain was post fixed for 12-24hr in the same fixative before being dissected into blocks for sectioning. Tissue was stained by washing 25 µm free floating postmortem human tissue sections for 3x 5 min in PBS and then permeabilizing them in a 0.2% triton X-100 PBS solution for 1 h at room temperature. Sections were subsequently blocked in a 10% BSA, 0.2% triton X-100 PBS solution for 1 h. at room temperature before applying appropriate primary antibodies in a 5% BSA, 0.2 % triton X-100 PBS solution O/N at 4°C. The following antibodies were used at the noted dilutions: COX2 (Abcam ab15191) used @ 1/500; NeuN (EMD Millipore MAB377) used @ 1/500; SOX9 (R&D Systems AF3075-SP); Biotinylated ricinus communis agglutinin (RCA I, RCA120) (Vector Laboratories B-1085) used @ 1/500. Sections were then washed for 3x 5 min in PBS before adding appropriate secondary antibody in 5% BSA PBS for 1 h. at room temperature. After washing for a further 3x 5 min in PBS sections were incubated in a 0.5% Sudan Black solution dissolved in 70% ethanol to reduce autofluorescence from lipofuscin vesicles. Sections were then washed a further 7x in PBS to remove excess Sudan Black before being spread onto slides and left to dry. Coverslips were mounted in 90% glycerol PBS containing Hoechst (diluted 1/1000).

SUPPORTING INFORMATION REFERENCES

1. Wagner, F. F. *et al.* Cyclooxygenase-2 inhibitors and uses thereof. U.S. Patent 20220348590, March 11th, 2022.
2. Orjales, A. *et al.* Novel 2-(4-methylsulfonylphenyl)pyrimidine derivatives as highly potent and specific COX-2 inhibitors. *Bioorganic Med. Chem*. 2008;16, 2183–2199. doi:10.1016/j.bmc.2007.11.079.
3. Singh, P. *et al.* 3-Substituted 1,5-Diaryl-1 H-1,2,4-triazoles as Prospective PET Radioligands for Imaging Brain COX-1 in Monkey. Part 1: Synthesis and Pharmacology. *ACS Chem. Neurosci*. 2018;9, 2610–2619. doi:10.1021/acschemneuro.8b00103.
4. Mastakov, M. Y. *et al.* Combined injection of rAAV with mannitol enhances gene expression in the rat brain. *Mol. Ther*. 2001;3, 225–232. doi:10.1006/mthe.2001.0246.
5. Schafer, D. P. *et al.* Microglia Sculpt Postnatal Neural Circuits in an Activity and Complement-Dependent Manner. *Neuron.* 2012;74, 691–705. doi:10.1016/j.neuron.2012.03.026.
6. Lehrman, E. K. *et al.* CD47 Protects Synapses from Excess Microglia-Mediated Pruning during Development. *Neuron.* 100, 2018;120-134.e6. doi:10.1016/j.neuron.2018.09.017.
7. Waldvogel, H. J. *et al.* The collection and processing of human brain tissue for research. *Cell Tissue Bank*. 2008;9, 169–179. doi:10.1007/s10561-008-9068-1.
